# Supplementary material for: Agrobacterium tumefaciens Deploys a Superfamily of Type VI Secretion DNase Effectors as Weapons for Interbacterial Competition In Planta
Source: Cell Host Microbe. 2014 Jul 9;16(1):94–104. doi: 10.1016/j.chom.2014.06.002 (PMC4096383; doi:10.1016/j.chom.2014.06.002)
Supplement: Document S1. Figures S1–S7, Tables S1 and S2, and Supplemental Experimental Procedures [file mmc1.pdf]

Cell Host & Microbe, Volume *16*

Supplemental Information

Agrobacterium tumefaciens Deploys a Superfamily of Type VI Secretion DNase Effectors as Weapons for Interbacterial Competition In Planta

Lay-Sun Ma, Abderrahman Hachani, Jer-Sheng Lin, Alain Filloux, and Erh-Min Lai

Cell Host & Microbe, Volume *16*

Supplemental Information

Agrobacterium tumefaciens Deploys a Superfamily of Type VI Secretion DNase Effectors As Weapons for Interbacterial Competition *In Planta*

Lay-Sun Ma, Abderrahman Hachani, Jer-Sheng Lin, Alain Filloux, and Erh-Min Lai

TABLE S1. Bacterial strains and plasmids, related to Experimental Procedures.

Strain /plasmid	Relevant characteristics	Source/Ref.
A. tumefaciens		
C58	Wild type virulent strain containing nopaline-type Ti plasmid pTiC58	Eugene Nester
ΔT6SS	Deletion of both *t6ss* promoter (Δ*pro*) and *vgrG2* operon	This study
Δ*tssL*	*tssL* deletion mutant	(Ma et al., 2009)
Δ*tae-tai*	Deletion from *atu4346* to *atu4347*	(Lin et al., 2013)
Δ*tde1-tdi1*	Deletion from *atu4350* to *atu4351*	This study
Δ*4349-tde1-tdi1*	Deletion from *atu4349 to atu4351*	This study
Δ*tde2-tdi2*	Deletion from *atu3639 to atu3640*	This study
Δ3TIs	Deletion from *atu3639 to atu3640*, *atu4350* to *atu4351,* and *atu4346* to *atu4347*	This study
Δ*tde1-tdi1* Δ*tde2-tdi2*	Deletion from *atu3639 to atu3640* and from *atu4350* to *atu4351*	This study
P. aeruginosa		
PAK	Wild type *P. aeruginosa*	A. Filloux
Δ*retS*	In-frame deletion of *retS* (PA4856) in PAK	(Goodman et al., 2004)
Δ*retS*ΔH1	H1-T6SS cluster deletion in Δ*retS*	(Hachani et al., 2013)
E. coli		
Top10	Host for DNA cloning	Invitrogen
BL21(DE3)	Host for overexpressing genes driven by the T7 promoter	(Studier et al., 1990)
**Plasmids**		
pRL662	Gm^R, broad host range vector derived from pBBR1MCS-2	(Vergunst et al., 2000)
pET22b(+)	Ap^R, *E. coli* overexpression vector to produce C-terminal His-tagged protein	Novagen
pET28a(+)	Km^R, *E. coli* overexpression vector to produce N or C-terminal His-tagged protein	Novagen
pJQ200KS	Gm^R, suicide plasmid containing Gm^r and *sacB* gene for double crossover event selection	(Quandt and Hynes, 1993)
pTrc200	Sm^R,Sp^R, pVS1 origin *lacI*^{q}, *trc* promoter expression vector	(Schmidt-Eisenlohr et al.,

		1999)
pJN105	Gm^R, arabinose-inducible gene expression vector derived from pBBRMCS-1, *araC*-P_{BAD}	(Newman and Fuqua, 1999)
pJN4347	Gm^R, pJN105 expressing toxin Tae (Atu4347)	This study
pJN4347(ssPelB)	Gm^R, pJN105 expressing toxin Tae (Atu4347) with N-terminal PelB signal peptide (ssPelB)	This study
pJN4350	Gm^R, pJN105 expressing toxin Tde1 (Atu4350)	This study
pJN3640	Gm^R, pJN105 expressing toxin Tde2 (Atu3640)	This study
pTrc4346	Sp^R, pTrc200 expressing immunity protein Tai (Atu4346)	This study
pTrc4351	Sp^R, pTrc200 expressing immunity protein Tdi1 (Atu4351)	This study
pTrc3639	Sp^R, pTrc200 expressing immunity protein Tdi2 (Atu3639)	This study
pTrc4349	Sp^R, pTrc200 expressing Atu4349	This study
pTrc3641	Sp^R, pTrc200 expressing Atu3641	This study
pTrc3640-strep	Sp^R, pTrc200 expressing C-terminal Strep-tagged Tde2 (Atu3640)	This study
pTrc4350-HA	Sp^R, pTrc200 expressing C-terminal HA-tagged Tde1 (Atu4350)	This study
pTrc4349-4352	Sp^R, pTrc200 expressing wild type proteins from Atu4349 to Atu4352	This study
pTrc4349-4352 (H190A D193A)	Sp^R, pTrc200 expressing Atu4349, Tde1 with amino acid substitution (H190A D193A), Tdi1, and Atu4352	This study
pTrc4349-4352 (H190A)	Sp^R, pTrc200 expressing Atu4349, Tde1 with amino acid substitution (H190A), Tdi1, and Atu4352	This study
pTrc4349-4352 (D193A)	Sp^R, pTrc200 expressing Atu4349, Tde1 with amino acid substitution (D193A), Tdi1, and Atu4352	This study
pTssL	Gm^R, pRL662 constitutively expressing TssL (Atu4333)	(Ma et al., 2009)
pRL3639	Gm^R, pRL662 constitutively expressing Tdi2 (Atu3639)	This study
pRL4349	Gm^R, pRL662 constitutively expressing Atu4349	This study
pRL4351-strep	Gm^R, pRL662 constitutively expressing C-terminal Strep-tagged Tdi1 (Atu4351).	This study
pJQ200KS- *pro*	Gm^R, plasmid to generate *t6ss* promoter deletion mutant	(Lin et al., 2013)
pJQ200KS-*vgrG2OP*	Gm^R, plasmid to generate *vgrG2* operon deletion mutant	This study
pJQ200KS-*atu4346-atu4347*	Gm^R, plasmid to generate *atu4346* to *atu4347* deletion mutant	(Lin et al., 2013)
pJQ200KS-*atu3639-atu3640*	Gm^R, plasmid to generate *atu3639* to *atu3640* deletion mutant	This study
pJQ200KS-*atu4350-atu4351*	Gm^R, plasmid to generate *atu4350* to *atu4351* deletion mutant	This study
pJQ200KS-*atu4349-atu4351*	Gm^R, plasmid to generate *atu4349* to *atu4351* deletion mutant	This study

TABLE S2. Primers used in this study, related to Experimental Procedures.

Plasmids	Primer sequence (5 '-3')[a]
pJN4347 or pJN4347(ssPelB)	CATGCCATGGGCCGCGTTAACTTTGACAC
	TAATACGAGCTCTCAGGACCCGCGGCTGG
pJN4350	CATGCCATGGGCAGTGCGACGACAACTGT
	ATCCGAGCTCTCAAGACACCGGGACGTCA
pJN3640	GGATTCCATATGAGTATCCCTCGCGACAA
	CGGGATCCTACCATTGTCATGTTCCTG
pTrc4346	TATAGGTACCGTTTGCAGCTCACGTCGT
	GCTCTAGACCACTAGTTACTTTTCTGCT
pTrc4351	TATAGGTACCACGGCAATCCTGACGT
	GCTCTAGACTAGCTGCCAATAGTACGA
pTrc3639	TATAGGTACCGATCTTCGACTTTGCCC
	GCTCTAGATTACCTCGCCGAACCGATT
pTrc4349	TAATACGAGCTCAGGTGAAAGTGGCTC
	TATAGGTACCTCATGCGGGCGCTCCGGAT
pTrc3641	CATGCCATGGCGACGGATCATTTTCAG
	TATAGGTACCTCATGCTGCTCCCTTG
pTrc3640-strep	CCGCTCGAGGTACCAAACAACGCTTACCCTG
	GCTCTAGATCACTTTTCGAACTGCGGGTGGCTCCATGTTCCTGTTAATGGCT
pTrc4350-HA	CATGCCATGGTGATCGACCACACCGT
	AAACTGCAGAGACACCGGGACGTCA
pTrc4349-4352	TAATACGAGCTCAGGTGAAAGTGGCTC
	GCTCTAGATGCTGGATATCGTCGT
pTrc4349-4352 (H190A D193A)	TAATACGAGCTCAGGTGAAAGTGGCTC
	ACCAAA**G**CCAAGGTA**GC**GGTTGCGGCCAAC
	AACC**GC**TACCTTGG**C**TTTGGTTGCGGG
	GCTCTAGATGCTGGATATCGTCGT
pTrc4349-4352 (H190A)	TAATACGAGCTCAGGTGAAAGTGGCTC
	CAAATCCAAGGTA**GC**GGTTGCGGCCAA
	TTGGCCGCAACC**GC**TACCTTGGATTTG
	GCTCTAGATGCTGGATATCGTCGT
pTrc4349-4352(D193A)	TAATACGAGCTCAGGTGAAAGTGGCTC
	CCCGCAACCAAA**G**CCAAGGTATGGGT
	ACCCATACCTTGG**C**TTTGGTTGCGGG
	GCTCTAGATGCTGGATATCGTCGT

pRL3639	CCGCTCGAGATCTTCGACTTTGCC
	GCTCTAGATTACCTCGCCGAACCGATT
pRL4349	CCGCTCGAGGTGAAAGTGGCTCCT
	GCTCTAGATCATGCGGGCGCTCCGGAT
pRL4351-strep	TTCCGCTCGAGACGGCAATCCTGACGT
	AATGCGGCCGCTACTTTTCGAACTGCGGGTGGCTCCAGCTGCCAATAGTACGAA
pJQ200KS-*vgrG2OP*	1. GCTCTAGATCGCTGAGTGATCGCCATCG
	2. CGGGATCCATTCATCAGGAACCTCGATAGC
	3. CGGGATCCACGAGATGAGCCACGCCTGTG
	4. TCCCCCCGGGGCAGCAACTCGCCATCAGTG
pJQ200KS-*atu3639-atu3640*	1. GCTCTAGACGTTCATATAGATGTCATT
	2. CGGGATCCTAAGGCATGCGCGTACGG
	3. CGGGATCCACTCATGCTGTCTCCCTTG
	4. AAACTGCAGGAACGACTGGACTGGAAG
pJQ200KS-*atu4350-atu4351*	1. GCTCTAGACAATCCTGACAAGGCCACAGC
	2. CGGGATCCACTCATGCGGGCGCTCCGGA
	3. CGGGATCCAGCTAGAGGGATATTTAAATGG
	4. AACTGCAGGGTGCAGGGCTATATTTATGC
pJQ200KS-*atu4349-atu4351*	1. GCTCTAGAGCATCATGAACACGATCATCG
	2. CGGGATCCGTTCATAATCAAATCCTGACAAAC
	3. CGGGATCCAGCTAGAGGGATATTTAAATGG
	4. AACTGCAGGGTGCAGGGCTATATTTATGC

a: Restriction enzyme sites are underlined, and mutated sequences are indicated by bold type.

Supplementary Figure 1

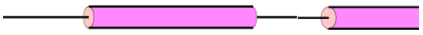

```
                                               CCCCCCCCHHHHHHHHHHHHHHHHC   CCCHHHHHHHH
Atu4350 (15890633)                   94 : -PEGATPKQVEEFRRQLKEQQDEIN---RMEPDDLVRNI-------------------------- : 128
Atu3640 (15891300)                  345 : FMPTKSTIDIDEFKRQMELQEQGLN---NMSPQQMLANQ-------------------------- : 380
Rleg4DRAFT_5191 (393183392)          93 : -PEGSTPEQIEEFKRQLKEQQDAIN---EIPPDTLVKNM-------------------------- : 127
PJE062_674 (211959488)              288 : PDHLDDDE----FKRQLDEQEDEIN---NTSADKLVERR-------------------------- : 319
BUC_4514 (217394038)                307 : NAKGDPK----EYDRQLLEQEKGLN---DLSVKEYLEGR-------------------------- : 338
VCHE48_1081 (445935354)             354 : KNKQSRKDKAAELDRQLADQQNGLN---NMSVDEYLAGRGAFLGRNPCNPDQKVPKVKRDPIIAK : 415
VMA_001767 (262025037)              354 : KNKQSRKDKAAELDRQLADQQNGLN---NMSVDEYLAGRGAFLGRNPCNPDQKVPKVKRDPIIAK : 415
A1S_0551 (193076348)                318 : KTGKSQAELDAEFDRQLKRQEEGLN---RLTVEEYQQNR-------------------------- : 353
F971_00411 (479947885)              401 : NSLAVEEYLNKETDWQLANQQKGLN---EMSVEDYIQGR-------------------------- : 436
ATW7_01792 (119446764)             1053 : NFKGGKQALEKEFYKQLKAQQAGIN---KMSVGEYLQNRN-----------------------LL : 1091
Swoo_2338 (170726686)               503 : CFKKNKKGDAEEYDRQLKGQQNGLN---DMTVQQYLDNR-------------------------- : 538
Pput_0805 (48546051)                300 : YKIGEFKR-------QLNGQEDGLN---WMTVEEFLEK--------------------------- : 327
PSYMO_00285 (330886100)             303 : DRIPEFDR-------QLAGQEKGLN---DLTVDEYLKGR-------------------------- : 331
PSYR_0686 (66043953)                310 : SKVGEFER-------QLKGQEDGLN---RLTVEEYLKN--------------------------- : 337
PSPTO_2457 (28869652)               342 : MSLVERQKYLKTYSAQLRAQQDAIN---NMTAEEFKSARDSYKN--------------------- : 382
D187_004203 (528053360)               1 : ----------------MRRQQDAIN---EMSVDKFESARKSYKDA-------------------- : 26
C800_03411 (507739969)              194 : KFQNNPHY-EKEMRRQLKMQEDGIN---KLTVFEWLTNR-------------------------- : 228
BCERE0025_58760 (228709322)           3 : -FNRNVKHDSEEFARQLKDQEKGMNELTELTVDEYLKNS-------------------------- : 40
CLONEX_01718 (151383)               377 : VFNYKSKFDEKEFARQLENQEKGLN---SLTIAEFVENR-------------------------- : 412
SEVCU071_1534 (365224737)            38 : SFKRNIKHDEKEFYRQLKNQEQGLN---KLTVKKYMDNR-------------------------- : 63
G362_17760 (516967442)               56 : PFKRNRKHDPVEYDRQYNEQMDALQ---QMPLSDWLRNR-------------------------- : 91
OUW_20586 (382939290)               562 : YTDKHAAEHAAEIDRQTQLWEHGFN---TQSVQQVLDNMD------------------------- : 598
Tpau_0235 (296026115)               536 : AAPTNPTHSPYERIRELLTQITWHN---SQTVQEAYDQA-------------------------- : 571
```

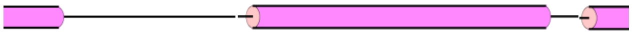

```
                                          HHHHHCCCCCCC          HHHHHHHHHHHHHHHHHHHHHHHHH  HCCCHHHH
Atu4350 (15890633)                  129 : -DKYRQQGRPTDD----------AANRRQSREDYRTDRTRELEEKYL--SKGRNDYK-------- : 172
Atu3640 (15891300)                  381 : -AKYLANPAGMR------------ALSEPLQAKARQEYRNDPRIQKKYVDQYGPIQG-------- : 424
Rleg4DRAFT_5191 (393183392)         128 : -DTYEDLGRGAKD----------AVDRANAREAWIKNRAAEIVKQDP--STTAKAAR-------- : 171
PJE062_674 (211959488)              320 : --QAIRDAGGTKG-----------VRDHKAQQDAREKHLRDRMLELR--EQQGLSRS-------- : 361
BUC_4514 (217394038)                339 : -ARYQEIGRAGTG---------AAQEQARAKYSRELANQFKDALNEQ--GIFGKAAK-------- : 383
VCHE48_1081 (445935354)             416 : KERIKETKRRQNDKIDDYINNQSLLEDKKAQHGLTSEKAVQKKLKEF--GMEAEAKKRGWSKETI : 478
VMA_001767 (262025037)              416 : KERIKETKRRKKNKIYEYINNQSLLEDKKAQYGLTSEKAVQKKLKEL--GMAETAKKEKWNANEI : 478
A1S_0551 (193076348)                354 : -QLYEKYKRAGTG---------TQQQRIREDMQRQLEESEYKKLKTEQPQLSKGQIER------- : 401
F971_00411 (479947885)              437 : -KAFDTEGRGGGAP----------AEQARKKYGADLAEKYEKEYQQQ--NIGAREAK-------- : 480
ATW7_01792 (119446764)             1092 : TELTDKHGHDKAR---------KILTRNGAAQLSARKELEETIFDSVIDSLERKGILG------D : 1141
Swoo_2338 (170726686)               539 : -KAYNAIGRKGTG-----------AAQKEAREKFRDKLISEYKNDLIDSGEYFGQEA-------- : 583
Pput_0805 (48546051)                328 : -----VENPNQRN--------KGLAQRARKKEQEFIKEKIEKELRK---TMGPFEAQ-------- : 368
PSYMO_00285 (330886100)             332 : --EAFKSGEALRD--------PKVAADARKLLGRKMELNIFRELRLG--GMSPEVAE-------- : 376
PSYR_0686 (66043953)                338 : -----IANPVKRD--------AMAAKKARTDLKDTLQERFQREFQK---EMSPLDAE-------- : 378
PSPTO_2457 (28869652)               383 : ------LGRNPAA--------DAMQRRMGKQMEREVAEKIQKSLVK--KGVDVREAI-------- : 423
D187_004203 (528053360)              27 : -LKATGSGRNPDA--------QRAREVERAAFEEKIRDSLMESMKNDNKGLGYRELK-------- : 74
C800_03411 (507739969)              229 : -KTFREKGR---------------LSSQTAQNDARDAYKRRKMFDYMLLSAENFKYD-------- : 269
BCERE0025_58760 (228709322)          41 : -ERYIAQGRA--------------IEGNAAQQAAREEAYVQKVNELQ---REGLTLS-------- : 79
CLONEX_01718 (151383)               413 : -KKYNVAGRN--------------KKANQFQKELRDKFKQDKIEELF---ENGMSYD-------- : 451
SEVCU071_1534 (365224737)            64 : -EKYIKNGID--------------IEAKHYQMLMREKAIKRKFQELL---SSGVSIK-------- : 102
G362_17760 (516967442)               92 : -TEYLQNGRT--------------PDSLRAQENARAAALKAKILELR---EQGQSRS-------- : 130
OUW_20586 (382939290)               599 : -----ATRPPSTG---------LRDELREGLSDYGYEDLRKAGYGPQQAEQLAKTYAN------- : 642
Tpau_0235 (296026115)               572 : ------KNRTYSS-----------AEAEQARKDFRDQLRTSGYEALLEMGYRPDAADS------- : 612
```

Supplementary Figure 1

```
                                   HHHHHHHHHHHHHHHHHCCCCCCCCCCCCCC          CCCCCCCCCCCCCCCHHH
                                                  *  *
Atu4350 (15890633)                173 : ---EQAANDVAEEMKKLAATHTLDLVAGGDGSIS---------GLGDKSINSSLGSQWKGR---- : 221
Atu3640 (15891300)                425 : ------PIKLGEYLDSAAALHNPDMIAGGKYNSVVDQTLPIENRIGGLSENSSMGSQWINPNR-- : 481
Rleg4DRAFT_5191 (393183392)       172 : ---EAAAN----DIKTMDVIHTPDLSAGGTGRLSTEN------GGMGPRSANRSIGSQWSKTGP-- : 222
PJE062_674 (211959488)            362 : ----EAKQIAMKELKKLAATHTLDIIAGGDPSNISG--------MGNRSTNSSLGAQWKGR---- : 410
BUC_4514 (217394038)              384 : ---EQAEAMAADRMKTLAALHNPDMIAGGKDVVT---------MMGDRGVNSSIGSQWKD----- : 431
VCHE48_1081 (445935354)           479 : IRNVTAMADSQHEMRSLAALHNPDMVAGGVDKIT---------GFGDKNTNSMIGASWSSGKE-- : 532
VMA_001767 (262025037)            479 : ALEVTAMVASKHEMRSLAALHNPDMVAGGLDKIT---------GFGDKNINSMIGASWSSGKE-- : 532
A1S_0551 (193076348)              402 : ----MAENNAKKTLEGLDVLHNPDMQLGGFDVKYDPKKPPTLDDFGHSGVNRSIGSQMAAK---- : 458
F971_00411 (479947885)            481 : ---KLAKEKSGRIMNEMAALHNPDQLVGGANKVDTK-------AIGLKNVNSSIGSQWKTR---- : 531
ATW7_01792 (119446764)           1142 : EAIKLANEKVKSQMSDLAALHDPDLIAGGDDKIR---------KLGNKNINSSLGSQWSKS---- : 1193
Swoo_2338 (170726686)             584 : --VEKATNLAMKEMKTLNALHNPDMIAGGKDEVFD---------LGNSSVNQSIGSQWKSKGTE- : 636
Pput_0805 (48546051)              369 : ---RVAIEKARNRMSITAALHNPDLVAGGR--DVIS-------DFGDRQVNSVIGAQWK------ : 415
PSYMO_00285 (330886100)           377 : ---ATAKKEVLEKMTTLAALHNPDIIAGGK--DVIN-------DLGDRRINSSIGPQWP------ : 423
PSYR_0686 (66043953)              379 : ---EAAIKKARETMASLAGLHNPDLTAGGK--DIIA-------DFGDRQVNSSIGPQWR------ : 425
PSPTO_2457 (28869652)             424 : ---IQAKARAKEIKSTVAALHEPDMVAGGWLSPDPV-------RMGSSSVNSSIGGSWS------ : 472
D187_004203 (528053360)            75 : ---NQASARAKDIMSTLDALHEPDMVTGGWSKSKPE-------GMGDASVNSAIGASWNQK---- : 125
C800_03411 (507739969)            270 : ----EITKKVEDELSSLAALHNPDQIAGGNFDDVTA--------MGDKRINSSIGSQWGTKDKGR : 322
BCERE0025_58760 (228709322)        80 : ----NAKKKAKEWLDTQAALHNPDQIAGGKVEIIGG--------MGDKRINSSIGSQWRYR---- : 128
CLONEX_01718 (151383)             452 : ----DAVKVADEWIKSKAVLHNPDQIAGGNSMNLTG--------LGDKNVNSSLGAQWRYR---- : 500
SEVCU071_1534 (365224737)         103 : ----EAERQSEIWLKKQAALHNLDMIAGGYAHNIGG--------LGDTKINSSLGSQYKYK---- : 151
G362_17760 (516967442)            131 : ----EAAENASSWLATQAATHRLDGIAGGNVTDISG--------VGDARINSSLGSQWRSR---- : 179
OUW_20586 (382939290)             643 : -----EQFPKDSGLIEQPVIHNPDGAIGGYKDALTY---------GDWQVNNALGNLTKQE---- : 689
Tpau_0235 (296026115)             613 : ----LSKSIAEQLMKKLVVTHNPDRIVGGRSDKMVT--------FGDSRTNSSIGNANQRG---- : 661
```

```
                                        HHHHHHHHHHHHHHHHHC CCCCEEEECCCCCCCCCCCCCCCCCCCCCCCCCCCCCC
Atu4350 (15890633)                222 : --------RSEQLRSHAKKAAEQK-KKMNAKLEECKPEGGNDNSPDAETPDNGTGKDGNP-DVP : 276
Atu3640 (15891300)                482 : ----NGHTRASRLTEHAKRQAANNCPSVQVDLRLCPSNPSRPGEPLTGT---------------- : 536
Rleg4DRAFT_5191 (393183392)       223 : ---NSDKTRLQQLKEHAQKAKEKG-ERTNADLKICEDGKSSKSGKKSGDSSGGSGKGQGGPGNVP : 283
PJE062_674 (211959488)            411 : ------RSQSLEDEAKRMQKDGKGKEKMNVKLEKC------------------------------ : 439
BUC_4514 (217394038)              432 : -----RVAELDDAAKKVP-ESERGGTKMNAKLKRCK----------------------------- : 461
VCHE48_1081 (445935354)           533 : ---NSRVSLLDKQACKEANEKGNGSKKMNVELVRCANKGKKS----------------------- : 571
VMA_001767 (262025037)            533 : ---NSRVSLLDKQACKEANEKGNGSKKMNVELVRCAKKRGK------------------------ : 570
A1S_0551 (193076348)              459 : -----KRLANMDAAAAQAKAKGMGKDAMDVELKRCK----------------------------- : 489
F971_00411 (479947885)            532 : -------VQALDEAAAKVPVSERGTTGMNAKLERCKK---------------------------- : 561
ATW7_01792 (119446764)           1194 : -----GRVAGMDLAASNSL---------------------------------------------- : 1207
Swoo_2338 (170726686)             637 : ---KSSRVALMDIEAEKALAESGPNTKMNVDLHRCK----------------------------- : 669
Pput_0805 (48546051)              416 : -----TRIPNLKKAAERVSLESRGSIRMNVKLHKC------------------------------ : 445
PSYMO_00285 (330886100)           424 : -----SRIGELDRAANLVPNELRNATKINAKLERCK----------------------------- : 454
PSYR_0686 (66043953)              426 : -----PKIQNLKAAAEKVPETMRDSTFLNVKLHKC------------------------------ : 455
PSPTO_2457 (28869652)             473 : -----SRLKALDDAVDSAMLDGNGGAKLNVRLNVLRG-ATTP----------------------- : 508
D187_004203 (528053360)           126 : -----DRLSTIDAQAKQASDAKQGHAKMNVKLEVCRGRRYCP----------------------- : 162
C800_03411 (507739969)            323 : AQNLEDELLKVLEGPPKIDEEQQKYIKMNVIFAEDLEIIK------------------------- : 362
BCERE0025_58760 (228709322)       129 : ---IDIVDEQIKELAKNMTPEQLKSTYLNVKLTH------------------------------- : 159
CLONEX_01718 (151383)             501 : ---IDDLEKQIMSFANKIPENEWESIKLNIKLTYIKKGE-------------------------- : 536
SEVCU071_1534 (365224737)         152 : ---IDDLDYQIKEQSKKFNEKELEKIYLNIILN-------------------------------- : 181
G362_17760 (516967442)            180 : ---VGDIDAAVVRFIQSHPGADLSDVYMNVTFR-------------------------------- : 209
OUW_20586 (382939290)             690 : -----------KDALRAWLQTQDPNAIVNINLGRR------------------------------ : 713
Tpau_0235 (296026115)             662 : ---AAAYLAWLEVQMKKNPDAIVRFDFVEPDDPETPSTTPPGGTDD------------------- : 704
```

Figure S1. Sequence alignment and secondary structure prediction of the Tde family containing the toxin_43 domains, related to Figure 2A and 7. The sequence alignment of the toxin_43 domain of the representative Tde superfamily proteins generated by use of ClustalW shows the conserved HxxD catalytic motif (* on conserved H and D residues). The amino acid position of residues shown is indicated on each side of the sequences. The locus tag and GI number are shown on the left of each sequence and the conserved amino acid residues are shaded in black for identity and in grey for similarity. Secondary structure of Tde1 was predicted by using the PSIPRED server and is indicated on the top of the sequence alignment. C, coiled-coil; H, alpha-helix; E, beta-sheet.

Supplementary Figure 2

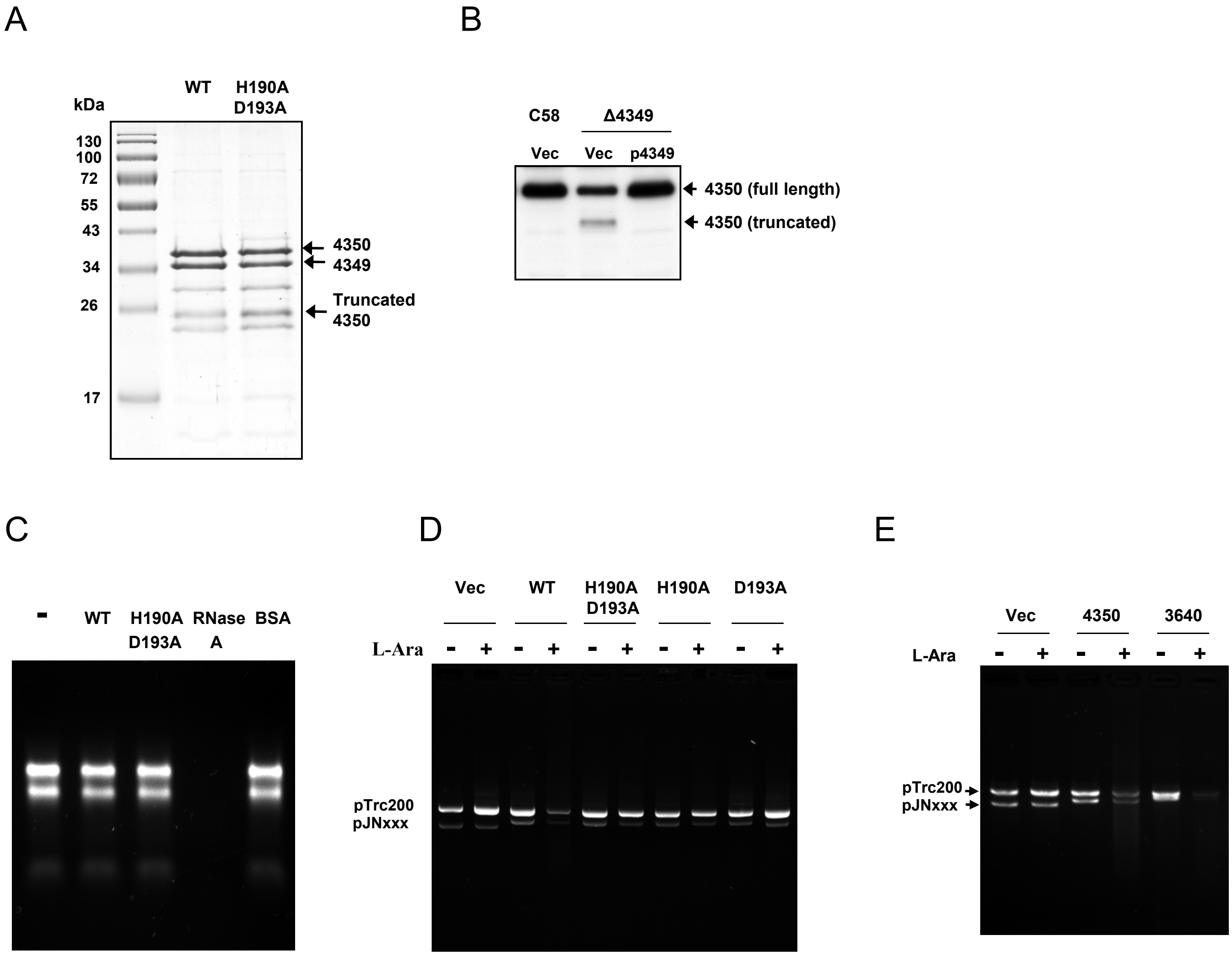

Figure S2. Protein analysis and nuclease activity assay, related to Figure 2. (A) SDS-PAGE analysis of purified wild-type Atu4350 (WT) and its variant (H190A D193A). In total, 2.5 μg of Ni^{2+}-affinity purified His-tagged fusion protein (WT or H190A D193A) co-produced with Atu4349 was loaded in each lane and visualized by Coomassie Blue staining. Full-length Atu4350 and Atu4349 proteins as well as truncated Atu4350 are indicated. Molecular weight standards are shown in kilo daltons on the left. (B) Western blot analysis of Atu4350 stability in *A. tumefaciens* wild-type C58 or the *atu4349* deletion mutant (Δ*4349)* harboring pTrc200 (Vec) or pTrc200 expressing Atu4349 (p4349) grown in AB-MES (pH 5.5) liquid medium. Full-length and truncated Atu4350 proteins are indicated. (C) RNA degradation analysis. An amount of 2 μg of *E. coli* total RNA treated with buffer (-), Atu4350 wild-type (WT), Atu4350 mutant (H190A D193A), RNase A, or BSA was analyzed by RNA-formaldehyde gel. (D) Atu4350 degrades plasmid DNA in *E. coli*. The *E. coli* DH10B cells containing pTrc200 and pJN105 (Vec) or the derivatives expressing wild-type 4350 (WT) or catalytic site mutants (H190A D193A, H190A, D193A) were induced with (+) or without (-) L-arabinose (L-Ara) for 2 hr. An equal volume of plasmids extracted from the same cell mass was analyzed on 1% agarose gel. (E) Atu4350 and Atu3640 degraded plasmid DNA in *E. coli* cells. *E. coli* DH10B cells containing pTrc200 and pJN105 (Vec) or derivatives expressing wild- type Atu4350 or Atu3640 were incubated with (+) or without (-) L-arabinose (L-Ara) for 2 hr. An equal volume of plasmids (pTrc200 and pJNxxx, indicating pJN105 or derivatives) extracted from the same cell mass was analyzed by 1% agarose gel electrophoresis.

Supplementary Figure 3

```
Atu4351 (15890632)               ------------MPELFITYENALKRFGLP---ENPEIMGEADIARYKNRIPETYLDFIRHAGLGIWKQGYFQFCNPEKY :  65
Atu3639 (159185838)              ---------MVEQPNYNVFETKWIEELLNAYEIDHIVKPSAQIVERYKDRLPEILVKFWIEKGWCSWSKGQYWICDPSIF :  71
Rleg4DRAFT_5192 (393183393)      ------------MNNYQARLDSIVQDFGSP---ESG--TAALDLGHYRGKVPEAMIDFWQQNGIGAVLDGYFQFCDPGQY :  63
PJE062_861 (211959674)           ------------MLSEDFESLFEDIDRPRE-----FRKIQSSEAKSLKESVPDDLFEYFTVYGRSILFEGRYQTCHPDDM :  63
BUC_4515 (7393834)               MPSLSDANSEDMVMDNFMENFLGFEGFGPP---MARRDVPLEKIEKFRGKLPNKLLEYWQEYGWCGYAKGLLWTVDPDEW :  77
VCHE48_1082 (40049333)           ---MSFKFQWTAKMQQGFDEFEHSMGVHRGFPIVSSTPVPAEVLEQYNGVLPENLLAYWERHGWCGYDNGMFWTVNPNDY :  77
VMA_001768 (262025038)           ---MAFKFQWSEVMDSKFERFQRRMGTA-----FVSQEVPLQALEYYEGILPTNLLAYWERYGWCGYNNGLYWTVNPDDY :  72
A1S_0552 (126640623)             --------------------------------------MTEDIIKKYRGKLPESILEQWHLFGFAGYLNGLYWITNPDDY :  42
F971_00412 (479947886)           -------------MDENFEIFYHDEGFGPP---VCVEPVSEETLKKYQGKLPNQLLEYWRAFGFSGWGNGLFWLVNPEDY :  64
ATW7_01785 (119444997)           -----------------------------------STPVTNETLQKFKGRLPSRLLEYWQEYGFCGFMDGFFWIVDPDDY :  45
Swoo_2339 (70726687)             -------------MNKFFDNFYNFAGFGPA---VKSKQPTAEELEFFEDKLPNRLLEYWQEYGFCGWGEGLIWVVNPADY :  64
Pput_0806 (148546052)            -------------MDKVFASLI--ENFGPP---IDRREVPGSSIERYRDKLPPKLLEYWSEHGWGGYGEGIFWLVNPQEY :  62
PSYMO_00280 (330886099)          -------------MDEDYAFFL--KKFGPA---IEQQAVPPSSIERYKKRLPEQLLKYWEDFGWCGYAKGLFWTVNPQDY :  62
PSYR_0687 (66043954)             -------------MDKVFARFL--EKFGGP---VDRQEVPASSIERYRGKLPSQLLEYWTEHGWCGYGDGIFWIVNPQEY :  62
PSPTO_2458 (28869653)            ------------MRDESFEIFV--DAMGEP---VKVCPASESVIDKYKGVLPDVLLDYWRSEGWCGYADGLFWTVNPDEY :  63
D187_004202 (528053359)          ------------MRDEAFEVFI--EDIGEA---SFRTPALELSFEKYQKVLPKQFLEYWKEEGWCGYAEGLFWTVNPEDY :  63
C800_03412 (507739970)           -------------MYEMFLFNSVNSKITQN--------VNEEFILKYSDYSCEQLNSLWKEVGLGSYYNGLFKIIEPNDL :  59
BCERE0025_58750 (228709321)     -------------------MEQYLKDFKLH------NKVPSEIIEKYKRVVPNEIINLWSNYGFGTFMQGYFKSVNPEEF :  55
CLONEX_01717 (210151382)        --------------------MSIFSDFKEN------YKFDNITEEKIKKIFPKELSDILCNYGSGDFLNGYFRFINPFEY :  54
SEVCU071_1533 (365224744)       --------------------MIEIRDFKKV------ANVPQEIINKYENILSEEIIEFWKDYGLGTFFEGFMKSINPEEY :  54
G362_17755 (516967440)          --------------------MVQIEDFVAH------SPVSQEVIAAYRDRVPSEIVEIWEQYGYGTFGEGFIRVINPSVF :  54
OUW_20591 (382939291)           ------------MADEDFEYFLTKLPLSIP-----GPACTDEHVRVYTSLVPDCLISYWQEFGFSGFGNGTAWLVDPIEW :  63
Tpau_0236 (296137982)           ------------MTDDHFELFLRDVPLTTA-----GPACTQQHLDTYRGVLPNPLLSYWQEYGFSGFGDGIVWLTDPLEW :  63
```

GAD-like domain

```
Atu4351 (15890632)               KSIVALALGGDKQLN----PVRTHALGFSAFGKILAWNEDY-KTTEINILLHRVTCRGLFKEIP-AERSDINLGIA--VE : 137
Atu3639 (159185838)              QPVIDYVFLGDSELD----PTRMVAFGYNAFGNVDIWYGDATIRLNIPNGMVRVEPRGYDEGQK-RQWTDEVMIGLKLSF : 146
Rleg4DRAFT_5192 (393183393)      SGILKLVFGGDTDIR----PEQTHALGFGAFGTIIAWNEVH-QDVTIDLVKGQVSCSALVN----GKRYDPNLAVTGQLM : 134
PJE062_861 (211959674)           RGVLSLIFRADKDFT----HKNCHAFLFSSFGEISFWHQEHGYGSVNLLSGEVICRSFTKKTVR----GPGFLKTFIVGF : 135
BUC_4515 (7393834)               EDELDAWVGETEFME----RDAYYVIARTAFGELILWGEKTGQSLKIITPYGMIFPSFDESK-FERRGPDLSIQLFF--S : 150
VCHE48_1082 (40049333)           TEIIERLSAISQLDD----PSENFVIARGAFGDLEIWNRKRGNVMCYCPVIGLLYQWHIDVKQE-KTEAELESAMASFFW : 152
VMA_001768 (262025038)           REIIERLSAISQLDD----PSENFVIARGAFGDLEIWNRKRGNVMYYSPVIGLLYQWDIDVKPE-KTEAELESDMASFFW : 147
A1S_0552 (126640623)             AEVIYDWLEETPLPD----DDVYHVLARSAFGELLIWGERNYGRYYIKTMEGILHDNGEQLES-----AEFYGSDFF--F : 111
F971_00412 (479947886)           QDVLDAWLEHIELPP----HEEYFVIARTAFGDLSIWGTIHGRCFTISAATSQIFPKMEKME---QGEEDLLIKIFF--S : 135
ATW7_01785 (119444997)           EDVLDAWIDDTFIVE----TDSFYVIGRNAFGDLYLWGEKTGYKYKISTSNGWIIEKVGDENDIKKNNALRAIKMFFYKS : 121
Swoo_2339 (70726687)             ADILMTWLSDTPFEN----ADNYYVIARSAFGELIVWGEKTGQSIDINVNFGMIFPTDNTEK-LKKRGEERSIELFF--A : 137
Pput_0806 (148546052)            DAVVSCWIAGTALAS----HDSYHLVARSAFGDLYLWGEKTGFSLEITSVGSQYIFYRTEFTK---EQLNTELQGFI--L : 133
PSYMO_00280 (330886099)          EDLLTDWLVGKEAFK----NDNYHVIARSAFGELYLWGEKSADSLSITSYMSRYSTNNSIFAD---GEKDFGIRVFF--S : 133
PSYR_0687 (66043954)             EGVVASWLEGTKFEK----RDNYHLIARGAFGDLYLWGEKTGFSLKITSVFSRCVIHDFEITT---EEMNRELQGFL--L : 133
PSPTO_2458 (28869653)            KHLLDMWLSGTKFSG----IDNYHVIARSAFGDLYAWGEKYGRKIIVSCPGDYIVSLADEAEIR-CDDPDLAIQSFF--A : 136
D187_004202 (528053359)          EGLVERWLSGTPFET----LDKYHVVARSAFGKLYAWGERNHRTLVILCPTSALIGIEKELRTP-AKDPDIAAQVFF--A : 136
C800_03412 (507739970)           KDIINQCYIMDDDES-------LLPFMCTAFGDVFAYVKNKRFGNYVVFLNIRYGTSLIIPDN------------FVAIF : 120
BCERE0025_58750 (228709321)     KEILEESSQR---------YKDSIVLFATGMGDLVIWS--DGYVRLINYRYGIVKTIMFTFE-------------FFFQN : 111
CLONEX_01717 (210151382)        QEVIKDTYFD---------AENSLPFMITAFGDVIVYKK-DGYIGIIKYKEKESAIIGKKIS-------------LFIRF : 111
SEVCU071_1533 (365224744)       TDLMEYGTQF---------FKDAVPLFVTGLGDIIYVEKKDGFMGILKFRYKETKVLMQMFN-------------LTIKL : 112
G362_17755 (516967440)          EAELGDRIGKTQGDG------IAIPVMVTALGDLITWEPSVDSLVAILFRKFETVGLGSPKT-------------FLG-- : 113
OUW_20591 (382939291)           KVTTEEVLLDVINHPRLGEDAKYIPFARSAFGKVWFWTPGYGISVIVDPVRGTMFFRQPARDSSVSGLEGTMKAFFG--- : 140
Tpau_0236 (296137982)           QATTTLITAGITHRP-LGTDATYIPILRTAFGKIWFWTPGFGRSLTITPAVGTASCIIQSAPRDLS-----LQATFA--- : 134
```

GAD-like domain

```
Atu4351 (15890632)               GIDAESFDAPDEKGKL-MFNRLLKNLGKLQLGQIYSPKLHPSLGG-QLTVENMRPVDALSAMTIAAQAGPFTLYDTTKPS : 215
Atu3639 (159185838)              NVSPYVAPWEDEKYQN-MMPLALERIGQLEPGEIYGFAPALSIGG-RNNVEHLQKVKVPEHLLLLASLDSPTLYDYSPPS : 224
Rleg4DRAFT_5192 (393183393)      LIDDPTLDEYDANAKK-LFKRARSKLGKLGVGQIYGFRPILALGG-NRALANLTVYEALPHMAILAQAHEMQLMDNAPFP : 212
PJE062_861 (211959674)           ASETFEYDFYDTNDRP-LFSRARRKLGEIGIGECYGFVPALGLGG-VPELETLKKLKAPEHFAILAQLIDFQLIDVQGYG : 213
BUC_4515 (7393834)               TCSKEAFDFLDNDKAA-LFERALEKLGPLDHTTMYGFVPALALGG-TPMLERLQKLDAHVHLDILSQVTELHVMRDIAQD : 228
VCHE48_1082 (40049333)           RASSSDQDQEDEYEEP-LFERALDKLGPLAENEMYAFVPATCLGG-KWLLENLQKVDITTHIEMLMDLEEPRINIMR--- : 227
VMA_001768 (262025038)           TASSSDQDQEDEYEEP-LFERALDKLGPLAENEMYAFVPATCLGG-KWLLENLQKVDITTHIEMLMDLEEPRINIMR--- : 222
A1S_0552 (126640623)             LPKKNYLDYTDKNGNK-LFDRAVKKLGVLKADEMYAFEPALALGG-EESLQHLTKVNLPVHMKLLKQVTPLRLRTFEDLT : 189
F971_00412 (479947886)           SKDKESLDINDYKNKP-LFDRAVKKYGELSKNEMFGFEPALILGG-EAKLENVRKLPIISHLQFLASLDTPRMMLDIGKF : 213
ATW7_01785 (119444997)           AMKVEAFDFIDIDQEP-LFGRCKELHNPLEYDEVFAFEPAIFLGG-EPRIENITKVNIFAHLTMLASFGEKEILNQESLI : 199
Swoo_2339 (70726687)             SMSKDSLDKKDLDENP-LFERAMAKLGPLEADEMYGFVPALAIGG-APKLENLQKVKVLEHLTFLADLGEKTVMADIVAM : 215
Pput_0806 (148546052)            SREVESMDFNG------LFNPAKEKLGRLKHDEMYGFFPALMLGG-ADSLQHLKKVSAVEHLIFLAQLTDLQPYSFSEQP : 206
PSYMO_00280 (330886099)          SMQLEHNDLDS------FFEPALKNLGQLQSDEMYGFVPALALGG-PVELKNLQKVKTTEHLTFLSQLSPLQDWGFPDV- : 205
PSYR_0687 (66043954)             SRNVDSNDLED------LFKPARRQLGTLRHDEMYGFVPALMLGG-SASLDHIEKLKAVEHLILLSQLAELQPYRF---- : 202
PSPTO_2458 (28869653)            MSDREDFDMEDHDGIP-LFERAVQKLGCLNEDEMYGFQPALVLGG-SITLQNLVKLDLEVHLTILHQLSR---------- : 204
D187_004202 (528053359)          SKKKQELDMKDEAGKA-LFQRALKKLGPLAPDEMYGFEPALVAGG-RNRLENLQKVNLFVHLAIVRELAEPR-IPFAGVD : 213
C800_03412 (507739970)           NKVIPNQSFLKGWFDLENYAFVKEKIGEIDFDECYGYFPTLSMGG-NESIDNISIVKMIPYIDMNVQMIDVFERADKL-- : 197
BCERE0025_58750 (228709321)     IT---DLEFRDEDLSWQPYPEALKQYNELDYEECFGYTPLLGLGG-AKKVENLKKVKLKEHILIITSFMGPVQ------- : 180
CLONEX_01717 (210151382)        LE---DDSFKKINFDIPLYEDAIKLYGKLQNNECFGFVPLLPLGG-KKEANCLEKVNIKVHIELITELVGKIE------- : 180
SEVCU071_1533 (365224744)       IIDENDKYFCDSHIEPQMYFEALKTKEEPAYDECFGYVPILPLGG-KEEVAHMDSVKIREHILLITELTGPIND------ : 185
G362_17755 (516967440)          LTRLDGAEHLEEEFAWDPYPEAVARHGAVEFDESFIFVPLPSLGG-AGTVDTLQKRPTLSAIQVMVDLQGPIGH------ : 186
OUW_20591 (382939291)           ASDGERFDFLDNSEQP-MFDRVYEHLGALRFDELYGFAPGLRIGG-AAIVEATHLFQIHVHMALLRSVIGD--AWYVAG- : 215
Tpau_0236 (296137982)           TGDRERYDFDPKGFPGGLFEQVLTRCGPLTVDQVYHFTPSIVSDDDPVDISTAQVANIHEWLAAVKTEVGDWSTFYI--- : 211
```

DUF1851

Figure S3. Sequence alignment of the Tdi immunity protein family, related to Figure 7A. BLASTP analysis was performed and full-length sequence alignment with ClustalW showed 2 conserved domains of representative Tdi family proteins. The locus tag and GI number are on the left and the amino acid position of residues is on the right of the sequences. The GAD-like domain and the DUF1851 domain are underlined in blue and yellow, respectively. The conserved amino acid residues are shaded in black for identity and in grey for similarity.

Supplementary Figure 4

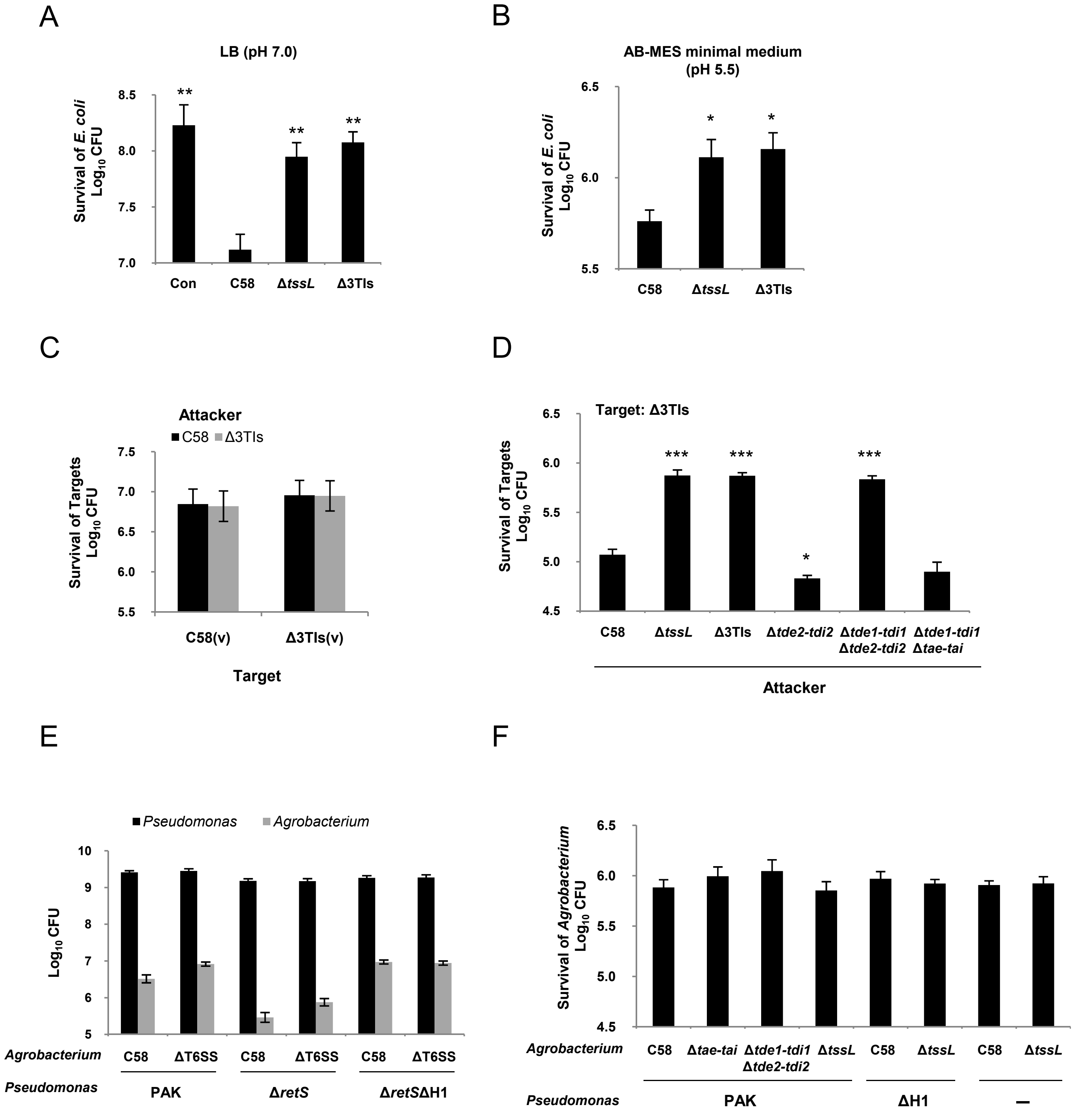

Figure S4. **Interbacterial competition assays, related to Figure 4 and 5.** (A) *A. tumefaciens* antibacterial activity assay against *E. coli* on LB. The *A. tumefaciens* wild-type C58, Δ*tssL*, or Δ3TIs mutant was co-cultured on LB (pH 7.0) agar with *E. coli* strain DH10B cells harboring the plasmid pRL662 to confer gentamicin resistance, at a ratio of 10:1. *E. coli* alone without contact with *A. tumefaciens* serves as a control (Con). (B) *A. tumefaciens* antibacterial activity assay against *E. coli* on AB-MES agar. The *A. tumefaciens* wild-type C58, Δ*tssL*, or Δ3TIs mutant was co-cultured on AB-MES (pH 5.5) agar with *E. coli* strain DH10B cells harboring the plasmid pRL662 at a ratio of 10:1. (C) *A. tumefaciens* intra-species competition on agar. The *A. tumefaciens* attacker strain (C58 or Δ3TIs) was mixed with the target strain (C58 or Δ3TIs) harboring pRL662 that confers gentamicin resistance at a 100:1 (attacker: target) ratio and co-cultured on AB-MES (pH 5.5) agar. The survival of target cells was quantified and no significant difference could be detected. Similar results were obtained by 10:1 (attacker: target) ratio (data not shown). (D) *A. tumefaciens* intra-species competition *in planta*. The *A. tumefaciens* attacker strain was mixed with the target strain harboring a gentamicin resistance-encoding vector pRL662 at a 10:1 (attacker: target) ratio, infiltrated into *N. benthamiana* leaves, and incubated at room temperature for 24 hr. The survival of target cells was quantified. (E) Cells of *P. aeruginosa* was mixed equally with *A. tumefaciens* harboring pRL662 and co-cultured at 28°C for 16 hr on LB agar. The survival of *P. aeruginosa* cells was quantified by growth on LB agar at 37°C for 12–16 hr before the emergence of visible *A. tumefaciens* colonies, which were quantified by growth on gentamicin-containing LB agar at 28°C for 48 hr. (F) Cells of *P. aeruginosa* and *A. tumefaciens* harboring pRL662 were mixed equally and infiltrated into *N. benthamiana* leaves and incubated at room temperature for 24 hr. The survival of *A. tumefaciens* cells was quantified by growth on gentamicin-containing LB agar. Data are mean ± SE of four biological replicates from three independent experiments (A, B) or three to six biological replicates from a minimum of two independent experiments (C, D, E, F). Significant difference compared with C58 was denoted as ***=*P* <0.0005, **=*P* <0.005, and *=*P* <0.05.

Supplementary Figure 5

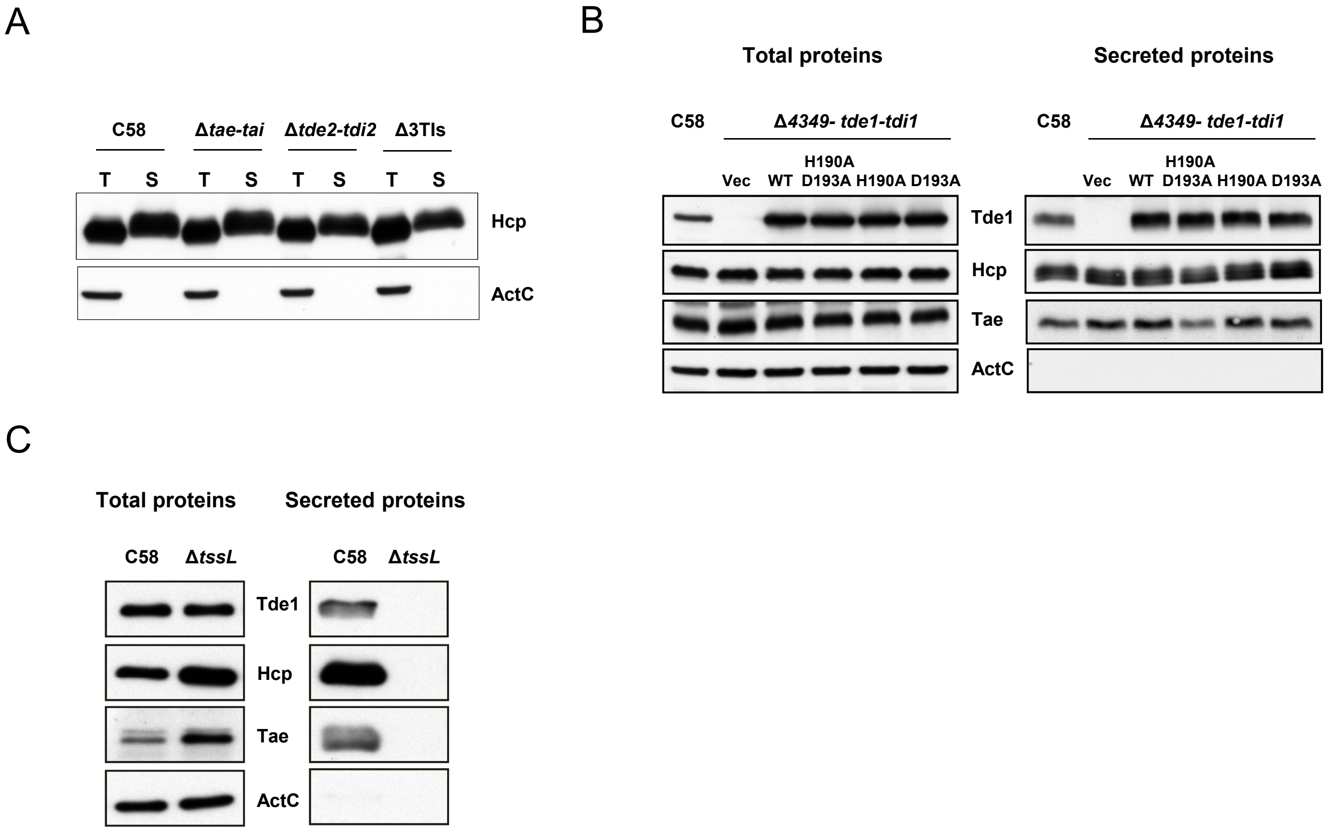

Figure S5. **Secretion assay, related to Figure 4 and Figure 5.** (A) Hcp secretion assay with wild-type *A. tumefaciens* C58, Δ*tae1–tai1*, Δ*tde2–tdi2*, and Δ3TIs (Δ*tae-tai* Δ*tde1–tdi1* Δ*tde2–tdi2*) grown in AB-MES (pH 5.5) liquid culture. Total (T) and secreted (S) proteins were isolated for western blot analysis of Hcp and ActC. ActC was a non-secreted protein control. (B) Secretion assay for various Atu4350 (Tde1) variants. Total and secreted proteins were isolated from the *A. tumefaciens* Δ*4349-tde1-tdi1* mutant containing vector pTrc200 (Vec) or derivatives expressing wild-type (WT) or HxxD variants of Tde1 grown on AB-MES minimal agar (pH 5.5) for western blot analysis of Tde1, Hcp, and Tae. (C) Secretion assay in LB medium. Total and secreted proteins were isolated from wild-type C58 and Δ*tssL* mutant grown in LB broth (pH 7.0) for 4-6 hr at 25°C for western blot analysis of Tde1, Hcp, and Tae.

Supplementary Figure 6

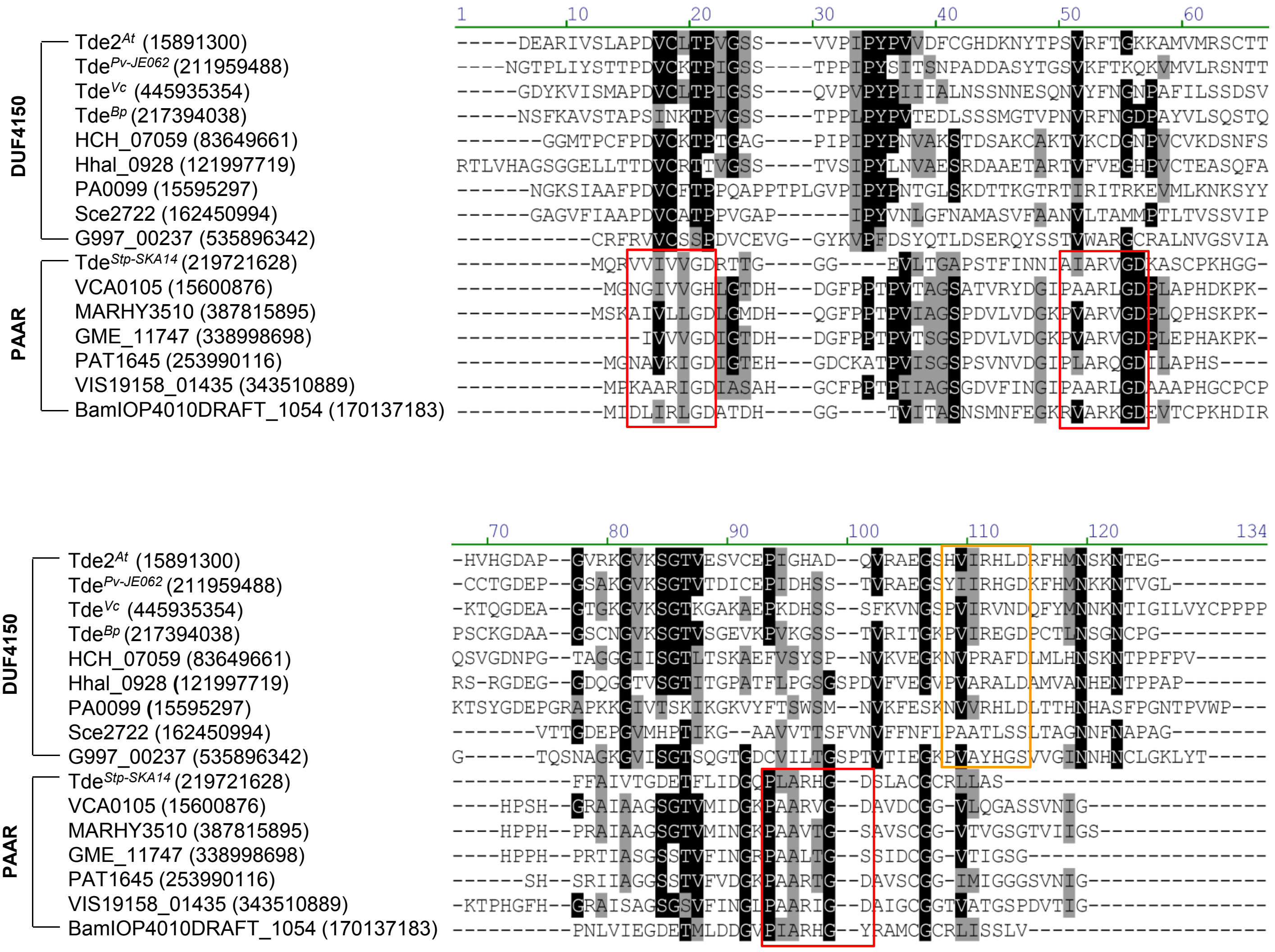

Figure S6. **Sequence alignment of DUF4150 and PAAR domains, related to Figure 7B.** Sequence alignment of DUF4150 domain and PAAR domain from selected Pfam family proteins was generated by using ClustalW. The locus tag and GI number are on the left and the amino acid position of residues is on the right of the sequences. The conserved amino acid residues are shaded in black for identity and in grey for similarity. PAARxGD motif is shown in a red box for PAAR-domain proteins and an orange box for DUF4150 domain proteins.

Supplementary Figure 7

	* 20 * 40 * 60
**Tde^{At}** (15890633)	---------KQVEEFRRQLKEQQDEINRMEPDDLVRNIDKYRQQGRPTDDAANRRQSRED
**Tde^{Bp}** (217394038)	--------KGDPKEYDRQLLEQEKGLNDLSVKEYLEGRARYQEIGRAGTGAA-QEQARAK
**Tde^{Vc}** (445935354)	--------KIDDYINNQSLLEDKKAQHGLTSEKAVQK--KLKEFG---------MEAEAK
**Tde^{Pp}** (48546051)	--------EYKIGEFKRQLNGQEDGLNWMTVEEFLE---KVENPNQRNKGLA-QR-ARKK
**RhsA** (307129607)	----------------AELRTGSGTNQ--SSRDYARS--------------------LGN
**RhsB** (307131672)	-----------------KIGDRQKGMIKDKLSTVKER--------------------SKA
**Cdi-CT$_{o11}^{Ec}$** (446167868)	----TATATVNASEVQGMQWGQGNMKQGMPWEDYVGKSLPADARLPKNFKIFDYYDGATK
**Colicin E7** (510385)	KRNKPGKATGKGKPVNNKWLNNAGKDLGSPVPDRIAN---------------KLRDKEFK
**Pyocin S3** (854363)	-------VKPLEVDIYGNFAGRPRDGN--HLDHMPDQ---------------------GA
**CdiA –CT^{Dda}** (307131201)	-------VKPLDVGSYKELKDRAVVGDGLEHDHIPSF---------------------AA

	* 80 * 100 * 120
**Tde^{At}** (15890633)	YRTDRTRELEEKYLS---KGRNDYKEQAANDVAEEMKKLA------ATHTLDLVAGGDGS
**Tde^{Bp}** (217394038)	YSRELANQFKDALNE-QGIFGKAAKEQAEAMAADRMKTLA------ALHNPDMIAGGKDV
**Tde^{Vc}** (445935354)	KRGWSKETIIRNV-T------------AMADSQHEMRSLA------ALHNPDMVAGGVDK
**Tde^{Pp}** (48546051)	EQEFIKEKIEKELRKTMGPF--EAQRVAIEKARNRMSITA------ALHNPDLVAGGRDV
**RhsA** (307129607)	QTDDAGHILGNVLGG-------------QGGKGNVFPQLP------AINRGQYRDFEKVV
**RhsB** (307131672)	LNTKMREHFNANEQK----------------IISEWEKQT------GMNWPTLSSGSRAT
**Cdi-CT$_{o11}^{Ec}$** (446167868)	TATSVKSIDTQTMAK-------------LANPNQVYSSIKGNIDAAAKFKEYALSGRELT
**Colicin E7** (510385)	SFDDFRKKFWEEVSK-------------DPELSKQFSRNN-------NDRMKVGKAPKTR
**Pyocin S3** (854363)	LATSLR-AIYPDIPY--------------GEIRKLMKKGG------SVAIPARVHQR--F
**CdiA –CT^{Dda}** (307131201)	LRTAKENELGRKLTP--------------AEEKTLYQNAT------AVEVPKDVHR---A

	* 140 * 160 *
**Tde^{At}** (15890633)	ISGLGDKSINSSLGSQWKG----RRSEQLRSHAKKAAEQK----KKMNAKLE--
**Tde^{Bp}** (217394038)	VTMMGDRGVNSSIGSQWKD-----RVAELDDAAKKVPESE-RGGTKMNAKLK--
**Tde^{Vc}** (445935354)	ITGFGDKNTNSMIGASWSSGKENSRVSLLDKQACKEANEKGNGSKKMNVEL---
**Tde^{Pp}** (48546051)	ISDFGDRQVNSVIGAQWKT-----RIPNLKKAAERVSLES-RGSIRMNVKLH--
**RhsA** (307129607)	KDYIGQHGS---VDIEWAF--------KYGNGGTRPTEI---------------
**RhsB** (307131672)	PHHVIPIKNGG--SNEWWN---------IIPVQHPHTGTI--------------
**Cdi-CT$_{o11}^{Ec}$** (446167868)	SSMISNREIQLAIPADTTK--------TQWAEINRAIEYGKSQGVKVTVTQVK-
**Colicin E7** (510385)	TQDVSGKRTSFELHHEKPIS---------QNGGVYDMDNISVVTPKRHIDIHRG
**Pyocin S3** (854363)	SETYGGRNTKEKQQKDASD---------LRAAVDSNFDAVKKGLLEEGF-----
**CdiA –CT^{Dda}** (307131201)	GPTYGGKNTAAQVQQDALD---------LCGAVCRDTDALRTNMIERGY-----

Figure S7. The Tde family is distinct from known DNase toxins, related to Figure 2A. The unique toxin domains from *A. tumefaciens* Tde1^{At}, *B. pseudomallei* Tde^{Bp}, *V. cholerae* Tde^{Vc}, *P. putida* Tde^{Pp}, *D. dadantii* 3937 RhsA and RhsB, *E. coli* colicin E7, *P. aeruginosa* Pyocin S3, *D. dadantii* 3937 CdiA-CT^{Dda}, and *E. coli* 869 CdiA-CT_{o11}^{Ec} were aligned by use of ClustalW. The conserved amino acid residues identified among Tde family proteins are shaded in black. The locus tag and GI number are on the left of each sequence.

SUPPLEMENTAL EXPERIMENTAL PROCEDURES

Protein purification

C-terminal His-tagged Tde1 (Tde1-His) and Atu4349 proteins were co-expressed in *E. coli* DH10B cells with the plasmids pJN105 and pTrc200, respectively. *E. coli* cells were grown to OD_{600} 0.7 in the presence of 0.5% glucose and 1 mM IPTG. Cells were harvested and resuspended in fresh LB medium with 0.2% L-arabinose and 1 mM IPTG. Growth was continued for another 2 hr to induce production of Tde1-His. The proteins were purified to homogeneity by nickel chromatography as previously described (Ma et al., 2012). Briefly, cells were lysed in Buffer A (20 mM Tris-Cl, 0.3 M NaCl, 0.5 mM DTT, 20 mM imidazole, and 20% glycerol, pH 7.5) and proteins were finally eluted from the nickel column in Buffer B (20 mM Tris-Cl, 50 mM NaCl, 250 mM imidazole, and 20% glycerol, pH 7.5).

In vitro RNase activity assay

Total RNA was extracted from *E. coli* DH10B (Lin et al., 2013) and 2 μg of total RNA was incubated with 0.2 μg of bovine serum albumin (BSA), Tde1, or RNase A in 10 μl of 50 mM Tris/HCl, 100 mM NaCl, and 10 mM $MgCl_2$ for 15 min at 37°C. The integrity of RNA was analyzed by RNA-formaldehyde gel.

TUNEL (terminal deoxynucleotidyl transferase dUTP nick-end labelling) and FACS (fluorescence-activated cell sorting) analysis

Overnight culture of *E. coli* DH10B strains harboring the pJN105 vector or derivatives expressing Tde toxins were harvested and adjusted to OD_{600} 0.3 with LB agar containing 0.2% L-arabinose. After 2-hr induction, cells (OD_{600} ~0.5) were washed with PBS buffer, fixed, and stained by use of the Apo-Direct Kit (BD Bioscience). The 3'-OH end of fragmented DNA was labelled with FITC-dUTP by terminal deoxynucleotidyl transferase. Propidium iodide (PI) labelling both intact and fragmented DNA was used as counterstaining. The PI-labelled and FITC-unlabelled *E. coli* cells were first gated at 10^2 with an FSC trigger with a threshold of 0.1%. The

FITC fluorescence was detected with a 529/28-nm filter in FL1 channel. The FL1-H signal < 10^2 was set as negative and > 10^2 was FITC-positive. At least 100,000 cells were collected for each sample. The intensity of fluorescence was determined by FACS analysis with the MoFlo XDP Cell Sorter (Beckman Coulter) and Summit V 5.2 software.

Interbacterial competition on agar plates

For interbacterial competition assay between *P. aeruginosa* and *A. tumefaciens,* overnight cultures of *P. aeruginosa* and *A. tumefaciens* containing pRL662 derivative conferring gentamicin resistance were grown in LB or 523 medium at 37°C and 28°C, respectively. The bacteria were sub-cultured for further growth for 4 to 5 hr under the same conditions. The bacterial cells were adjusted to OD_{600} 0.01, mixed at a 1:1 ratio, and 10 µl was spotted on LB (pH7.0) agar and incubated for 16 hr at 28°C. Cells were harvested, serially diluted, and plated in triplicates on LB agar with or without gentamicin for colony forming units (CFU) counting. Because of the higher replication rate of *P. aeruginosa*, the *P. aeruginosa* cell number was scored after 16-hr incubation at 37°C on LB agar without any antibiotics. *A. tumefaciens* cells were counted on gentamicin-containing LB agar plates after 2- days' incubation at 28°C. Similar procedures were used for *E. coli-A. tumefaciens* and *A. tumefaciens* intra-species competition assay except that the bacterial cells were co-cultured at a ratio of 10 (*A. tumefaciens* attacker cells at OD_{600} 0.1) to 1 (*A. tumefaciens* or *E. coli* DH10B harboring pRL662 target cells at OD_{600} 0.01) and grown on LB (pH7.0) or AB-MES (pH5.5) agar plates at 25°C for 16 hr. Target *E. coli* and *A. tumefaciens* cells were counted on gentamicin-containing LB agar plates for 16 hr at 37°C and 2-days' incubation at 28°C, respectively. At least three independent experiments or minimum of three biological replicates from two independent experiments were performed for all assays. Data represent mean ± standard error (SE) of all biological replicates. Statistics was calculated by Student's t test and the p-value was denoted as ***=P <0.0005, **=P <0.005, and *=P <0.05.

Interbacterial competition assay *in planta*

The intra-species *A. tumefaciens* competition assay was performed with a 10:1 attacker-to-target ratio by leaf infiltration of *Nicotiana benthamiana*. Briefly, 523 overnight-cultured *A. tumefaciens* cells were sub-cultured at 28°C in the same medium for further growth to OD_{600} 1.0-1.5. The harvested cells were resuspended in 1/2 Murashige and Skoog (MS) medium (pH 5.7) to an appropriate OD_{600}. The attacker (OD_{600} 5) and target (OD_{600} 0.5) were mixed equally before infiltration into 2-month-old leaves of *N. benthamiana* with use of a needleless syringe. After 24-hr

incubation at room temperature, the infiltrated spot was punched out, ground in 0.9% NaCl, serially diluted, and plated in triplicates on LB agar containing appropriate antibiotic to select for the target cells. Similar procedures were used for *A. tumefaciens-P. aeruginosa* inter-species competition assay, except the bacterial cells were adjusted to OD_{600} 1 mixed equally for infiltration. All assays were performed with at least two independent experiments and each with two biological replicates; or three independent experiments and each with one or two biological replicates. Data represent mean ± standard error (SE) of all biological replicates. Statistics was calculated by Student's t test and the p-value was denoted as ***=P <0.0005, **=P <0.005, and *=P <0.05.

REFERENCES

Goodman, A.L., Kulasekara, B., Rietsch, A., Boyd, D., Smith, R.S., and Lory, S. (2004). A signaling network reciprocally regulates genes associated with acute infection and chronic persistence in Pseudomonas aeruginosa. Dev. Cell *7*, 745-754.

Hachani, A., Lossi, N.S., and Filloux, A. (2013). A visual assay to monitor T6SS-mediated bacterial competition. J. Vis. Exp. e50103.

Kado, C.I., and Heskett, M.G. (1970). Selective media for isolation of Agrobacterium, Carynebacterium, Erwinia, Pseudomonas, and Xanthomonas. Phytopathology *60*, 969-976.

Lai, E.M., and Kado, C.I. (1998). Processed VirB2 is the major subunit of the promiscuous pilus of Agrobacterium tumefaciens. J. Bacteriol. *180*, 2711-2717.

Lin, J.S., Ma, L.S., and Lai, E.M. (2013). Systematic dissection of the Agrobacterium type VI secretion system reveals machinery and secreted components for subcomplex formation. PLoS One *8*, e67647.

Ma, L.S., Lin, J.S., and Lai, E.M. (2009). An IcmF family protein, $ImpL_M$, is an integral inner membrane protein interacting with $ImpK_L$, and its walker a motif is required for type VI secretion system-mediated Hcp secretion in Agrobacterium tumefaciens. J. Bacteriol. *191*, 4316-4329.

Ma, L.S., Narberhaus, F., and Lai, E.M. (2012). IcmF family protein TssM exhibits ATPase activity and energizes type VI secretion. J. Biol. Chem. *287*, 15610-15621.

Newman, J.R., and Fuqua, C. (1999). Broad-host-range expression vectors that carry the L-arabinose-inducible Escherichia coli araBAD promoter and the araC regulator.

Gene *227*, 197-203.

Quandt, J., and Hynes, M.F. (1993). Versatile suicide vectors which allow direct selection for gene replacement in gram-negative bacteria. Gene *127*, 15-21.

Schmidt-Eisenlohr, H., Domke, N., and Baron, C. (1999). TraC of IncN plasmid pKM101 associates with membranes and extracellular high-molecular-weight structures in Escherichia coli. J. Bacteriol. *181*, 5563-5571.

Studier, F.W., Rosenberg, A.H., Dunn, J.J., and Dubendorff, J.W. (1990). Use of T7 RNA polymerase to direct expression of cloned genes. Methods Enzymol. *185*, 60-89.

Vergunst, A.C., Schrammeijer, B., den Dulk-Ras, A., de Vlaam, C.M., Regensburg-Tuink, T.J., and Hooykaas, P.J. (2000). VirB/D4-dependent protein translocation from Agrobacterium into plant cells. Science *290*, 979-982.
