# Supplementary material for: Agrobacterium tumefaciens Deploys a Superfamily of Type VI Secretion DNase Effectors as Weapons for Interbacterial Competition In Planta
Source: Cell Host Microbe. 2014 Jul 9;16(1):94–104. doi: 10.1016/j.chom.2014.06.002 (PMC4096383; doi:10.1016/j.chom.2014.06.002)
Supplement: Document S2. Article plus Supplemental Information [file mmc2.pdf]

Cell Host & Microbe
Article

Agrobacterium tumefaciens Deploys a Superfamily of Type VI Secretion DNase Effectors as Weapons for Interbacterial Competition In Planta

Lay-Sun Ma,[1,2] Abderrahman Hachani,[2] Jer-Sheng Lin,[1] Alain Filloux,[2,*] and Erh-Min Lai[1,*]
[1]Institute of Plant and Microbial Biology, Academia Sinica, Taipei 11529, Taiwan
[2]MRC Centre for Molecular Bacteriology and Infection, Department of Life Sciences, Imperial College London, London SW7 2AZ, UK
*Correspondence: a.filloux@imperial.ac.uk (A.F.), emlai@gate.sinica.edu.tw (E.-M.L.)
http://dx.doi.org/10.1016/j.chom.2014.06.002
This is an open access article under the CC BY license (http://creativecommons.org/licenses/by/3.0/).

SUMMARY

The type VI secretion system (T6SS) is a widespread molecular weapon deployed by many Proteobacteria to target effectors/toxins into both eukaryotic and prokaryotic cells. We report that *Agrobacterium tumefaciens*, a soil bacterium that triggers tumorigenesis in plants, produces a family of type VI DNase effectors (Tde) that are distinct from previously known polymorphic toxins and nucleases. Tde exhibits an antibacterial DNase activity that relies on a conserved HxxD motif and can be counteracted by a cognate immunity protein, Tdi. In vitro, *A. tumefaciens* T6SS could kill *Escherichia coli* but triggered a lethal counterattack by *Pseudomonas aeruginosa* upon injection of the Tde toxins. However, in an in planta coinfection assay, *A. tumefaciens* used Tde effectors to attack both siblings cells and *P. aeruginosa* to ultimately gain a competitive advantage. Such acquired T6SS-dependent fitness in vivo and conservation of Tde-Tdi couples in bacteria highlights a widespread antibacterial weapon beneficial for niche colonization.

INTRODUCTION

Bacteria produce diverse toxic compounds, including diffusible small molecules such as antibiotics, that allow them to thrive in a competitive environment. They can also produce and secrete enzymatic toxins targeting nucleic acids, membrane lipids, or the peptidoglycan of competing bacterial cells (Benz and Meinhart, 2014; Braun and Patzer, 2013). The type VI secretion system (T6SS) is a molecular machine found in most Proteobacteria (Cascales, 2008; Filloux et al., 2008) and can deliver effectors to both eukaryotic (Pukatzki et al., 2007) and prokaryotic cells, which appear to be the major targets (Dong et al., 2013; English et al., 2012; Hood et al., 2010; Russell et al., 2011, 2012, 2013).

Functional and structural studies have shown that the T6SS nanomachine shares remarkable similarities with the bacteriophage tail structure (Basler et al., 2012; Brunet et al., 2014; Kapitein et al., 2013; Leiman et al., 2009). The system contains a TssB-TssC contractile sheath, which is proposed to accommodate the Hcp-VgrG tail tube/puncturing device. The contraction of the sheath leads to the propelling of Hcp, VgrG, and T6SS effectors across bacterial membranes (Basler et al., 2012; Bönemann et al., 2010; Kapitein et al., 2013; Leiman et al., 2009). Time-lapse fluorescent experiments highlighted the dynamics of this mechanism by revealing ‘‘T6SS dueling’’ between interacting cells (Basler et al., 2013; Basler and Mekalanos, 2012; Ho et al., 2014; LeRoux et al., 2012).

To date, only a few toxins have been biochemically characterized and shown to contribute to the bactericidal activity mediated by the T6SS (Russell et al., 2014). The most remarkable examples are the cell-wall-degrading effectors that include the type VI secretion amidase effector (Tae) and type VI secretion glycoside hydrolase effector (Tge) superfamilies (Russell et al., 2011, 2012; Whitney et al., 2013). The Tae family includes Tse1 from *Pseudomonas aeruginosa* (Russell et al., 2011) and Ssp1 or Ssp2 from *Serratia marcescens* (English et al., 2012). The Tge family includes the Tse3 muramidase from *P. aeruginosa* (Russell et al., 2011) and Tge2 and Tge3 from *Pseudomonas protegens* (Whitney et al., 2013). VgrG3 from *Vibrio cholerae* (Brooks et al., 2013; Dong et al., 2013) represents another effector family with a distinct muramidase fold unrelated to the Tge family (Russell et al., 2014). These enzymes are injected into the periplasm of target cells, where they hydrolyze the peptidoglycan, thereby inducing cell lysis (Brooks et al., 2013; English et al., 2012; Russell et al., 2011; Whitney et al., 2013). The phospholipase Tle superfamilies represent an additional set of T6SS toxins. By degrading phosphotidylethanolamine, a major constituent of bacterial membranes, these effectors challenge the membrane integrity of target cells (Russell et al., 2013).

A recent study reported the nuclease activity of two proteins, RhsA and RhsB from *Dickeya dadantii*, containing NS_2 and HNH endonuclease domains, respectively, which cause the degradation of cellular DNA and confer an intraspecies competitive advantage (Koskiniemi et al., 2013). However, whether the *D. dadantii* antibacterial activity mostly relies on the DNase activity, and whether Rhs proteins are delivered by a dedicated T6SS machine remains to be determined (Russell et al., 2014).

Agrobacterium tumefaciens is a soil bacterium that triggers tumorigenesis in plants by delivering T-DNA from bacterial cells into host plant cells through a type IV secretion system (T4SS) (Alvarez-Martinez and Christie, 2009; Gelvin, 2010). Although not essential for tumorigenesis (Wu et al., 2008), the

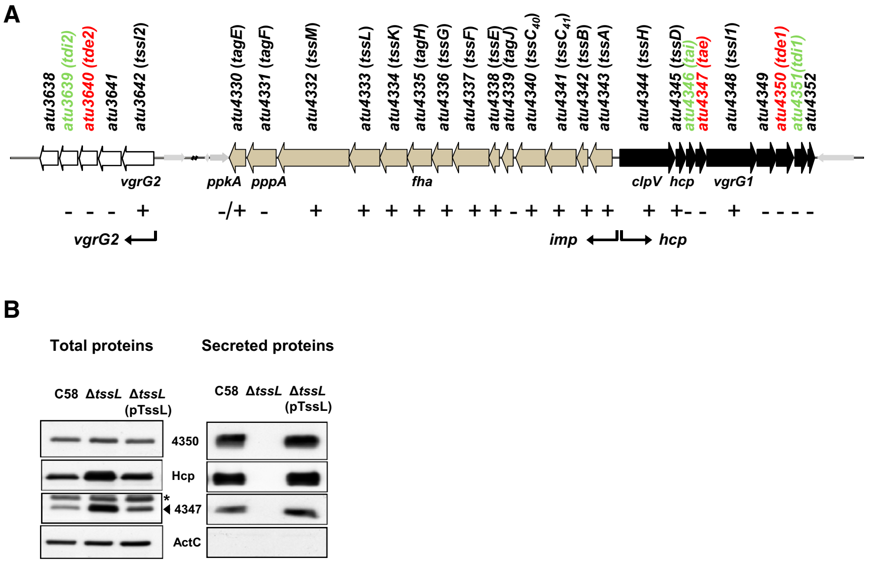

Figure 1. Atu4350 Is an *A. tumefaciens* T6SS-Dependent Effector

(A) *A. tumefaciens* T6SS consists of the major T6SS gene cluster containing two operons, *imp* (in gray; *atu4343* to *atu4330*) and *hcp* (in black; *atu4344* to *atu4352*), and another divergent operon named *vgrG2* (in white; *atu3642* to *atu3638*) (Lin et al., 2013). The genes are indicated with locus/common names and/or designated as *tss* (type VI secretion) or *tag* (type VI secretion-associated gene) based on the proposed nomenclature (Shalom et al., 2007). The three toxins and their cognate immunity proteins identified in this study are indicated in red and green, respectively. The genes, which are essential, nonessential, or partially required for Hcp secretion, are flagged as (+), (−) or (−/+), respectively.

(B) Secretion of Atu4350 is T6SS dependent. Total and secreted proteins were isolated from wild-type C58, Δ*tssL* mutant, and the complemented strain Δ*tssL*(pTssL) grown on AB-MES minimal agar (pH 5.5) for 24 hr at 25°C for western blot analysis of nonsecreted protein ActC (Liu et al., 2008), Hcp, and Atu4347, known T6SS-dependent secreted proteins. Asterisk * indicates the cross-reacting band of the antibody against Atu4347.

A. tumefaciens T6SS is activated at both transcriptional (Wu et al., 2012) and posttranslational levels (Lin et al., 2014) when sensing acidity, a signal enriched in the plant wound site and apoplast. Here, using *A. tumefaciens* as a model organism, we report the discovery of a type VI DNase effector (Tde) family that exhibits potent antibacterial activity. The toxic activity of the Tde DNase is counteracted by a cognate immunity protein, here called Tdi. The T6SS increases the fitness of *A. tumefaciens* during in planta colonization, and the bacterium uses Tde to attack both intraspecies and interspecies bacterial competitors. The widespread conservation of the Tde toxin and Tdi immunity across bacterial genomes suggests that an appropriate combination of a functional T6SS and a broad toxin repertoire is key to niche colonization within a polymicrobial environment.

RESULTS

Atu4350 Is an *A. tumefaciens* T6SS-Dependent Effector

A. tumefaciens strain C58 contains a T6SS gene cluster in which 14 of 23 genes are essential for the assembly of a functional type VI secretion machinery (Lin et al., 2013). The other genes are dispensable because the secretion of Hcp, a hallmark for T6SS activity, is not significantly affected in corresponding mutants (Figure 1A) (Lin et al., 2013). The gene *atu4347*, which is located in the so-called *hcp* operon (Figure 1A), encodes a T6SS-secreted protein predicted to act as a peptidoglycan amidase (Lin et al., 2013). The gene *atu4347* and its neighboring gene *atu4346* encode proteins orthologous to the *S. marcescens* T6SS antibacterial toxin secreted small protein (Ssp), belonging to the amidase family 4, and a cognate immunity, classified as resistance-associated protein (Rap), respectively (English et al., 2012; Russell et al., 2012). Because several genes encoded in the *hcp* operon (Figure 1A) are dispensable for type VI secretion, additional T6SS toxin-immunity gene pairs may exist within this operon.

Attempts to delete the *atu4351* gene were unsuccesful (Lin et al., 2013), which suggests that it may encode for a potential immunity protein protecting against the activity of a cognate toxin. This toxin is probably encoded by the adjacent gene, *atu4350*, and the secretion of Atu4350 is indeed readily detectable with growth of *A. tumefaciens* on acidic AB-MES minimal medium (pH 5.5), as was shown for the secretion of Hcp or Atu4347 (Figure 1B) (Lin et al., 2013, 2014; Ma et al., 2009, 2012; Wu et al., 2012; Wu et al., 2008). The secretion of Atu4350 is T6SS dependent, since it was abolished in a T6SS mutant, Δ*tssL* (Figure 1B).

A Superfamily of Type VI DNase Effectors

Atu4350 is annotated as a hypothetical protein, and no functional domains were identified by a BLASTP search of the NCBI database. A screening of the Pfam database linked the Atu4350 protein to a recently identified superfamily containing the putative domain toxin_43 (PF15604) (Zhang et al., 2012). This superfamily displays a conserved putative catalytic motif HxxD and exhibits an all-alpha helical fold feature (Figures 2A; Figure S1 available online). Furthermore, the members of this family are distinct from known polymorphic toxins and have been tentatively assigned a putative RNase activity (Zhang et al., 2012).

To investigate whether Atu4350 harbors a nuclease activity, we overexpressed a C-terminal His_6-tagged fusion of the protein in *Escherichia coli*. Atu4350 was then purified in the presence of Atu4349, which resulted in increased Atu4350 yield and stability (Figures S2A and S2B). Atu4350 did not display a detectable RNase activity in vitro (Figure S2C). Instead, it showed a Mg^{2+}-dependent DNase activity, as seen by the rapid degradation of supercoiled plasmidic DNA (pTrc200) (Figure 2B). The conserved HxxD motif is required for this DNase activity, since an Atu4350 derivative bearing amino acid substitutions within this motif (H190A D193A) lost its ability to degrade the pTrc200 plasmid

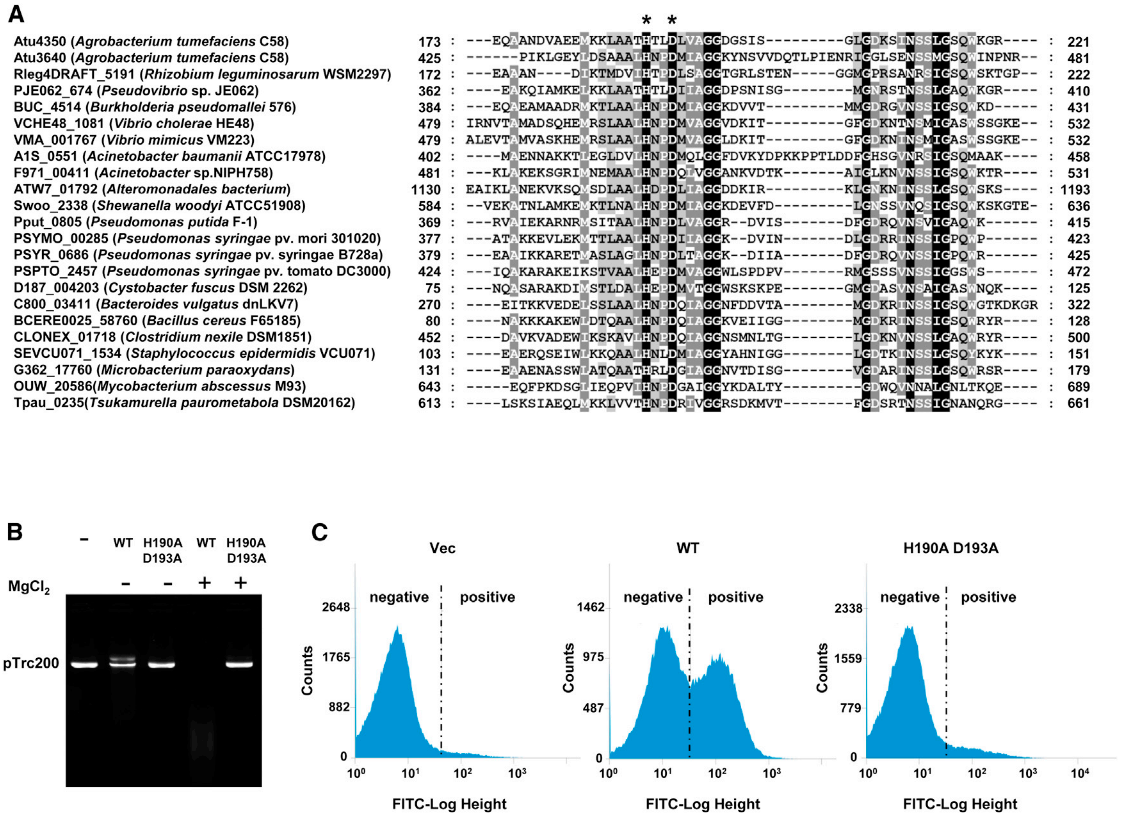

Figure 2. A Superfamily of Type VI DNase Effectors

(A) Partial sequence alignment of the representative Tde superfamily proteins that contain the toxin_43 domain showing the conserved HxxD catalytic motif. The locus tag and organism name are on the left, and the amino acid position of residues in the alignment is indicated on each side of the sequences. The conserved amino acid residues are shaded in black for identity and in gray for similarity. Asterisks (*) indicate amino acids in the HxxD catalytic motif, which were targeted for mutagenesis.

(B) In vitro DNase activity assay. The integrity of plasmid DNA (pTrc200) coincubated with purified proteins of the wild-type 4350 (WT) or the H190A D193A catalytic site mutant in the presence (+) or absence (–) of Mg^{2+} at 37°C for 1 hr was visualized with 1% agarose gel. Plasmid DNA with buffer (–) was a control.

(C) Detection of DNA fragmentation by TUNEL assay and analysis by cell sorting. *E. coli* cells containing pJN105 (vector) or derivatives expressing the wild-type Atu4350 or H190A D193A catalytic site mutant were induced by L-arabinose. Cells were fixed and stained with FITC-dUTP to detect the fragmented DNA by monitoring fluorescence intensity (indicated on the x axis) by cell sorting. FITC-labeled cells are indicated as positive, and cells with background FITC signal are indicated as negative. The counts for cell sorting are indicated on the y axis. Similar results were obtained from at least two independent experiments. See also Figures S1 and S2.

(Figure 2B). To assess the DNase activity in vivo, the *atu4350* gene and its derivatives were cloned under the control of an arabinose-inducible pBAD promoter in the plasmid pJN105. Induction of *atu4350* expression resulted in rapid degradation of the pTrc200 and pJN105 (or derivatives) plasmids (Figure S2D). Cells producing the Atu4350 variant with substitutions in the HxxD motif showed no DNase activity (Figure S2D). The Atu4350-dependent DNA fragmentation was also characterized by using terminal deoxynucleotidyl transferase dUTP nick-end labeling (TUNEL) with 3′-OH termini of DNA breaks labeled with FITC-dUTP. TUNEL-positive cells (FITC labeled) were observed in *E. coli* cells producing only wild-type Atu4350 but not the Atu4350 variant (H190A D193A) (Figure 2C). More precisely, ~50% of cells expressing Atu4350 but only ~8% of cells producing the Atu4350 variant (H190A D193A) showed FITC labeling. Our results establish that Atu4350 is a bona fide DNase.

Three Toxin-Immunity Pairs in *A. tumefaciens*

The *A. tumefaciens* T6SS activity also relies on the expression of an operon encoding *vgrG2*, which is functionally redundant with *vgrG1* for Hcp secretion (Lin et al., 2013) (Figure 1A). Standard bioinformatic tools showed that Atu3640 and Atu3639, encoded within the so-called *vgrG2* operon (Figure 1A), are homologous to Atu4350 and Atu4351, respectively (Figures 2A, S1, and S3). As observed with Atu4350, Atu3640 also possesses a C-terminal toxin_43 domain, and production of Atu3640 in *E. coli* cells caused rapid degradation of plasmidic DNA (Figure S2E).

Collectively, our results suggest that Atu4350-Atu4351 and Atu3640-Atu3639, together with the Atu4347-Atu4346 proteins, are potential T6SS toxin-immunity pairs in *A. tumefaciens*. Atu4350 and Atu3640 have DNase activity, whereas Atu4347 is a putative peptidoglycan amidase (English et al., 2012). We used a strategy based on the coproduction of a given toxin-immunity pair to investigate the role of the putative immunity in

protecting against the adverse effects of the toxin. The toxin gene was cloned under the control of an inducible promoter, whereas the putative cognate immunity gene was expressed from a compatible plasmid. The growth of *A. tumefaciens* cells harboring the vector controls increased steadily over time, with no growth upon induction of *atu4350* and *atu3640* expression (Figures 3A and 3B). The growth inhibition exerted by Atu4350 and Atu3640 was readily alleviated by the coexpression of the cognate immunity genes *atu4351* and *atu3639*, respectively (Figures 3A and 3B). Atu4350 and Atu3640 exert a toxic effect via their DNase activity when produced within the cytoplasm, whereas the putative peptidoglycan amidase activity of Atu4347 is likely to occur within the periplasm. Indeed, the fusion of Atu4347 to a cleavable N-terminal Sec-dependent signal peptide (ssPelB) led to a significant growth inhibition (Figure 3C). The growth inhibitory effect of Atu4347 was neutralized by the coexpression of the cognate immunity gene *atu4346*, predicted to encode a protein bearing a typical N-terminal signal peptide (data not shown).

In conclusion, we identified three toxin-immunity pairs. The Atu4347-Atu4346 pair belongs to the family type VI secretion amidase effector and immunity (Tae-Tai), and the toxin likely targets the peptidoglycan. Atu4350 and Atu3640 represent a family of T6SS toxins and are named Tde1 and Tde2, respectively, for Tde. Their cognate immunity proteins Atu4351 and Atu3639 are named Tdi1 and Tdi2, respectively.

The *A. tumefaciens* T6SS Has a Role in Bacterial Competition

The role of the three *A. tumefaciens* T6SS toxins Tae, Tde1, and Tde2 was assessed in bacterial competition, with T6SS-negative *E. coli* K12 cells used as prey cells (Dong et al., 2013; English et al., 2012; Hachani et al., 2013; Hood et al., 2010; Russell et al., 2011, 2012, 2013). *A. tumefaciens* and *E. coli* strains carrying gentamicin resistance were cocultured on LB (pH 7.0) or acidic AB-MES (pH 5.5) agar, and *E. coli* survival was monitored by counting gentamicin-resistant colony-forming units. *E. coli* survival was greatly reduced when cocultured with wild-type *A. tumefaciens* strain C58, as compared to *E. coli* alone or the *A. tumefaciens* T6SS mutant, Δ*tssL* (Figures S4A and S4B). Importantly, a strain presenting a functional T6SS, as shown by the high levels of Hcp secretion (Figure S5A), but lacking all toxin-immunity pairs (Δ3TIs) was unable to kill *E. coli*. These results demonstrate the antibacterial activity of the *A. tumefaciens* T6SS, which is relying on at least one of the three identified toxins, Tae, Tde1, or Tde2.

Tde Toxins Equip *A. tumefaciens* with a Plant Colonization Advantage

Despite its usefulness in identifying T6SS antibacterial activity, the *E. coli* K12 model does not provide information on whether

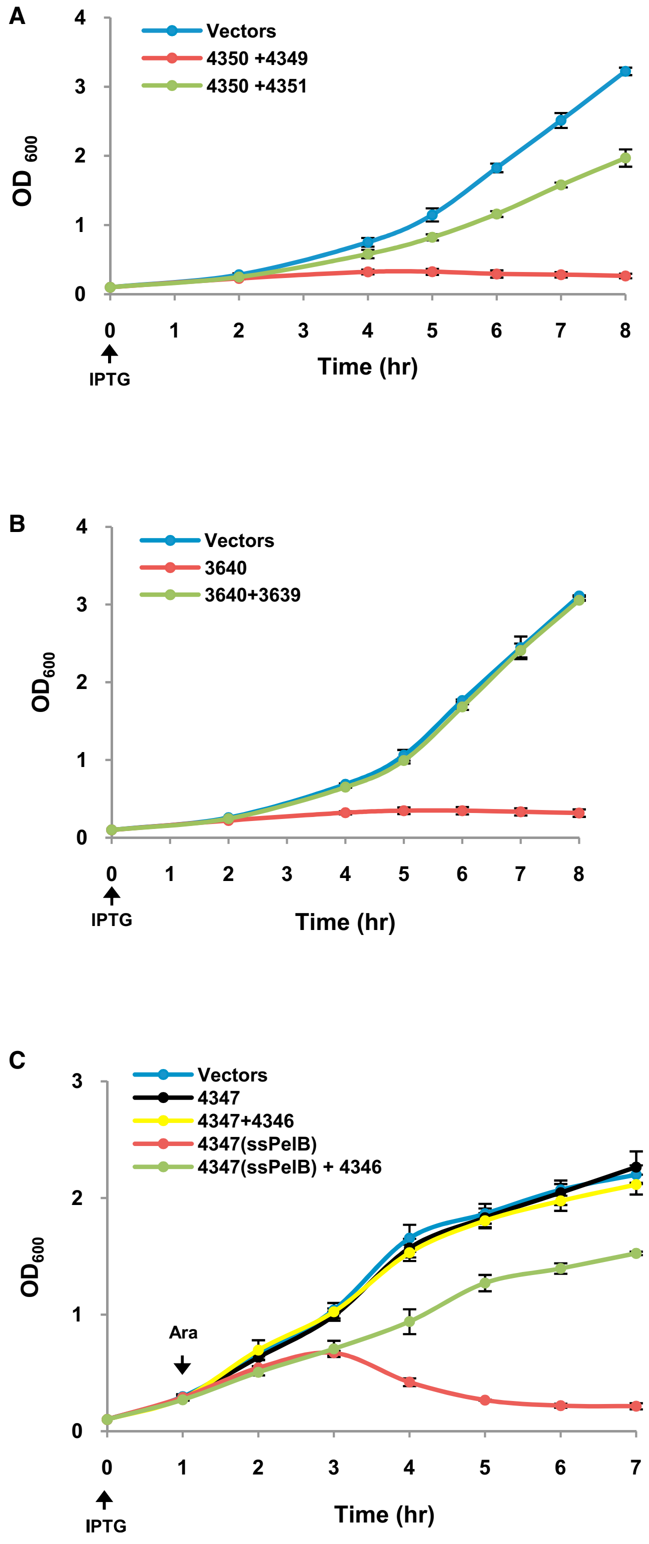

Figure 3. Three Toxin-Immunity Pair Analysis

(A and B) Cultures of *A. tumefaciens* wild-type C58 harboring the vectors (pTrc200 and pRL662) or derivatives were supplemented with 1 mM IPTG (at time 0 hr) for growth curve analysis. Atu4350 was produced from plasmid pTrc200, and the putative immunity protein Atu4351 or Atu4349 was constitutively expressed from plasmid pRL662 (A). Atu3640 was produced from plasmid pTrc200, and the putative immunity protein Atu3639 was constitutively expressed from plasmid pRL662 (B).

(C) *E. coli* DH10B cultures were induced at 0 hr with 1 mM IPTG for 1 hr to produce the putative immunity protein Atu4346 from plasmid pTrc200, then L-arabinose (Ara) induction of Atu4347 with or without signal peptide (ssPelB) from plasmid pJN105. Cell growth was monitored by measuring OD_{600} at 1 hr intervals. The growth of control cells carrying empty vectors was monitored in parallel. Data are mean ±SE of three (A) or two ([B] and [C]) independent experiments.

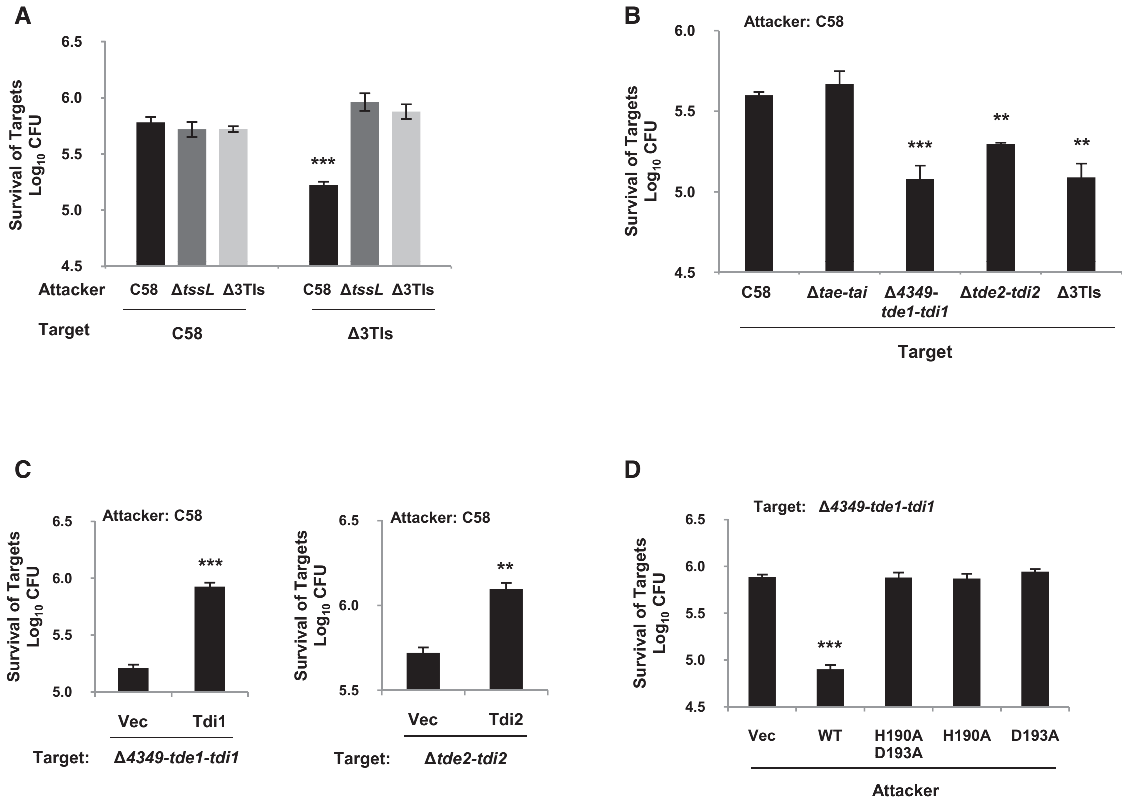

Figure 4. *A. tumefaciens* Intraspecies Competition In Planta

The *A. tumefaciens* attacker strain was mixed with the target strain harboring plasmids pRL662 or pTrc200 at 10:1 (attacker: target) ratio and infiltrated into *N. benthamiana* leaves. The survival of target cells was quantified by counting CFUs on antibiotics-containing LB agar.

(A) Attackers are wild-type C58, Δ*tssL*, or Δ3TIs (Δ*tae-tai*, Δ*tde1-tdi1*, Δ*tde2-tdi2*) coinfected with target strains C58 or Δ3TIs.

(B) Attacker wild-type C58 was tested against target mutants lacking single (Δ*tae-tai*, Δ*4349-tde1-tdi1*, or Δ*tde2-tdi2*) or triple toxin-immunity pairs (Δ3TIs).

(C) The attacker strain C58 was coinfected with the target strains (Δ*4349-tde1-tdi1* or Δ*tde2-tdi2*) harboring plasmid pTrc200 (Vector) or derivatives expressing the cognate immunity gene.

(D) Attacker strains containing vector pTrc200 (Vec) or derivatives expressing wild-type (WT) or catalytic site mutants of Tde1 (H190A D193A, H190A, or D193A) were tested against the target mutant strain Δ*4349-tde1-tdi1* harboring pRL662 plasmid. Data are mean ±SE ([B]: n = 3; [A], [C], and [D]: n = 4). Significant difference compared with C58 or Vec was denoted as *** = $p < 0.0005$, ** = $p < 0.005$, and * = $p < 0.05$. See also Figures S4 and S5.

a specific set of toxins can be advantageous for *A. tumefaciens*. Thus, we investigated the function of the T6SS antibacterial activity during interbacterial competition between *A. tumefaciens* strains. The *A. tumefaciens* attacker strain was mixed with target strains carrying gentamicin resistance to allow the quantification of surviving cells. Although Tde1 and Tae were readily secreted when bacteria were grown on acidic AB-MES agar plate (Figure 1B), the *A. tumefaciens* wild-type C58 strain had no significant growth advantage when cocultured with the strain Δ3TIs (Figure S4C).

However, the above described phenotypes may result from the limitations of an in vitro setup, which prompted us to assess the T6SS antibacterial activity in an environment closer to the in vivo situation. We thus assessed whether a functional T6SS and the associated toxins may give *A. tumefaciens* an advantage for survival inside the host plant. We used a combination of *A. tumefaciens* strains, which contain attacker and target cells, in coinfection assays. These strains carried the plasmid pRL662 encoding gentamicin resistance or pTrc200 conferring spectinomycin resistance, which allowed for selecting surviving cells within what we define here as the target cell population. The assay involved coinfiltration of *A. tumefaciens* attacker and target strains into *Nicotiana benthamiana* leaves (Anand et al., 2007). Coinfection with the *A. tumefaciens* wild-type C58 attacker strain caused a ~5-fold decrease in surviving cell numbers of the Δ3TIs target strain in comparison to the C58 target strain (Figure 4A). In contrast, coincubation of the Δ3TIs target strain with an attacker strain lacking a functional T6SS, Δ*tssL*, or the three T6SS toxins, Δ3TIs, resulted in wild-type levels of fitness. These results strongly suggest that the *A. tumefaciens* T6SS and its associated toxins provide a competitive advantage to this bacterium during plant colonization.

We monitored the contribution of each individual toxin-immunity pair in this experimental model. Target strains lacking Tde1-Tdi1 or Tde2-Tdi2 toxin-immunity pairs lost their competitive advantage against the wild-type C58 attacker (Figure 4B). Furthermore, the expression of a *tdi* immunity gene in the absence of the corresponding *tde* toxin gene was sufficient to protect the target strain against killing by the C58 attacker (Figure 4C). In contrast, the Δ*tae-tai* mutant showed wild-type levels

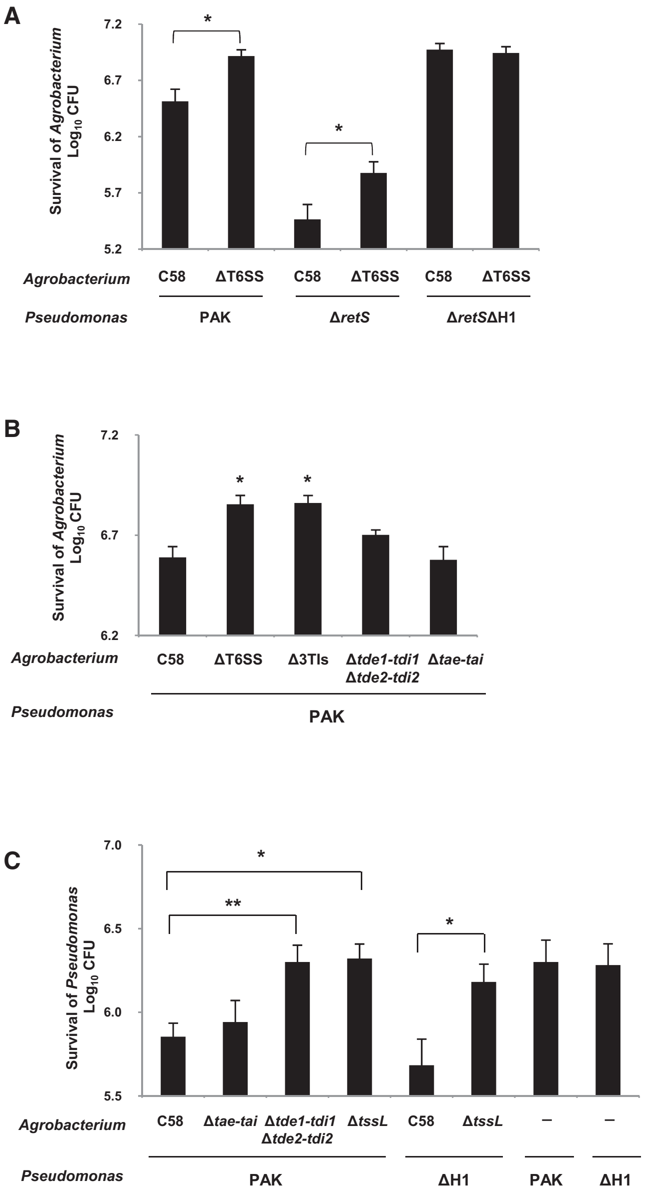

Figure 5. *A. tumefaciens-P. aeruginosa* Competition Assays

(A and B) *P. aeruginosa* and *A. tumefaciens* cells were mixed equally and cocultured on LB agar ([A] and [B]) or coinfected in planta (C).

(A) *P. aeruginosa* wild-type PAK, PAKΔ*retS* (Δ*retS*), or PAKΔ*retS*ΔH1 (Δ*retS*ΔH1) was cocultured with *A. tumefaciens* wild-type C58 or T6SS mutant (ΔT6SS).

(B) *P. aeruginosa* PAK was mixed with one of the *A. tumefaciens* strains C58, ΔT6SS, Δ3TIs, Δ*tde1-tdi1*Δ*tde2-tdi2*, or Δ*tae-tai* mutant.

(C) Cells of *P. aeruginosa* and *A. tumefaciens* harboring pRL662 derivative were mixed equally and infiltrated into *N. benthamiana* leaves. *P. aeruginosa* cell number was scored after 16 hr incubation at 37°C on LB agar without any antibiotics. Data are mean ±SE ([A]: n = 4–6; [B] and [C]: n = 3–4). Significant difference compared with C58 was denoted as ** = $p < 0.005$ and * = $p < 0.05$. See also Figures S4 and S5.

of fitness, which suggests that both Tde1 and Tde2, but not Tae, are crucial for *A. tumefaciens* competition during colonization in planta (Figure 4B). These observations are further supported by evidence showing that the presence of either of the *tde-tdi* toxin-immunity pairs is sufficient to attack the Δ3TIs target strain, but this ability is lost if the attacker is a double *tde-tdi* deletion mutant (Δ*tde1-tdi1*Δ*tde2-tdi2*) (Figure S4D). Importantly, attacking strains producing any variants of the Tde1 proteins (H190A, D193A, or H190A D193A substitutions) were unable to inhibit the growth of target cells (Figure 4D), which suggests that the Tde DNase activity was essential for providing the competitive advantage. Of note, mutations in the HxxD motif did not affect the secretion of Tde1, Hcp, or Tae (Figure S5B). These observations highlight the decisive role played by the Tde DNase toxins and their cognate immunity proteins in the fitness of *A. tumefaciens* during the colonization of the plant host.

A. tumefaciens T6SS Toxins Trigger a *P. aeruginosa* Counterattack In Vitro

Because multiple microbial taxa coexist as communities to compete for resources, we further investigated the impact of the *Agrobacterium* T6SS activity in the frame of an interspecies context. *P. aeruginosa* is an opportunistic pathogen for humans and plants (Rahme et al., 1995), but it also coexists with *A. tumefaciens* as common residents in freshwater, bulk soil, and the rhizosphere (Hu et al., 2003; Schmeisser et al., 2003; Troxler et al., 1997). We examined *A. tumefaciens-P. aeruginosa* competition in both in vitro and in vivo assays. For competition assay in vitro, we designed coculture conditions on LB agar (pH 7.0) for which type VI secretion is observed in both strains (Hachani et al., 2011) (Figure S5C) and measured the competition outcomes. Even though *A. tumefaciens* and *P. aeruginosa* cells were cocultured in equal amounts, *P. aeruginosa* outcompeted *A. tumefaciens* by at least 100-fold after 16 hr of coincubation (Figure S4E). H1-T6SS is constitutively active in the *P. aeruginosa* strain PAKΔ*retS* (Hachani et al., 2011), and this strain exerted a stronger inhibition on *A. tumefaciens* growth than the wild-type PAK strain (Figure 5A). Strikingly, upon contact with *P. aeruginosa*, the number of viable *A. tumefaciens* wild-type C58 cells was ∼5-fold lower than the isogenic ΔT6SS strain, suggesting that *A. tumefaciens* T6SS activity can trigger a *P. aeruginosa* counterattack. *P. aeruginosa* H1-T6SS is required for this counterattack as a mutant lacking this cluster (Δ*retS*ΔH1) was unresponsive to *A. tumefaciens* (Figure 5A). An *A. tumefaciens* mutant lacking all three pairs of toxin-immunity genes (Δ3TIs) displayed a higher survival rate when cocultured with the *P. aeruginosa* wild-type strain (Figure 5B). Because the *A. tumefaciens* strain Δ3TIs was still T6SS active (as shown by Hcp secretion) (Figure S5A), the presence of a functional T6SS may not be sufficient for *A. tumefaciens* to trigger a *P. aeruginosa* counterattack. Of note, the *A. tumefaciens* wild-type C58, as well as the isogenic variants Δ*tde1-tdi1*Δ*tde2-tdi2* and Δ*tae-tae* mutants, could still deliver at least one T6SS toxin and were all killed by *P. aeruginosa* (Figure 5B). These data suggest that the injection of *A. tumefaciens* T6SS toxins was required to trigger a *P. aeruginosa* counterattack.

A. tumefaciens Uses Tde as a Weapon against *P. aeruginosa* In Planta

The advantage provided by the Tde toxins to *A. tumefaciens* when grown in planta (Figure 4) but not in vitro (Figure S4C) underlines the importance of a physiologically relevant

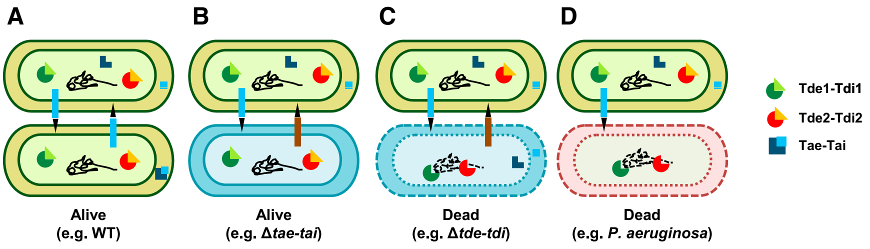

Figure 6. Illustration of *A. tumefaciens* Interbacterial Competition during In Planta Colonization

A. tumefaciens wild-type C58 (WT, green) injects Tde toxin (red or green circle) via the T6SS puncturing device drawn between the cells.

(A) None of the *A. tumefaciens* siblings is killed because of the presence of the Tdi immunity protein (orange or light green triangle) inactivating the injected Tde toxin from the WT.

(B) With Δ*tae-tai* lacking an amidase toxin-immunity pair (light blue), no killing occurs because Tae toxin is not the major antibacterial weapon during in planta colonization.

(C) Injection of Tde toxin from WT *A. tumefaciens* to its sibling Δ*tde-tdi* mutant (light blue) lacking the cognate immunity protein results in cell death caused by degradation of cellular DNA.

(D) Injection of Tde toxin from WT *A. tumefaciens* to *P. aeruginosa* (pink) results in cell death caused by degradation of cellular DNA.

environment for studying bacterial fitness. Thus, we investigated whether the relationship between *A. tumefaciens* and *P. aeruginosa* could differ in planta. Remarkably, the survival of *P. aeruginosa* wild-type PAK and its isogenic H1-T6SS mutant (ΔH1) was reduced by ~5-fold following 24 hr coinfection with *A. tumefaceins* wild-type C58 in leaves of *N. benthamiana* (Figure 5C). In contrast, we detected no significant growth difference for *A. tumefaciens* strains grown alone or coinfected with *P. aeruginosa* inside the host plant (Figure S4F). The *P. aeruginosa* attack against *A. tumefaciens* observed in vitro may be totally inefficient or prevented in planta. Furthermore, the Δ*tae-tai* strain retained the ability to attack *P. aeruginosa*, but Δ*tssL* or a strain lacking both *tde-tdi* (Δ*tde1-tdi1*Δ*tde2-tdi2*) were unable to kill *P. aeruginosa* (Figure 5C). During plant colonization, *A. tumefaciens* is able to attack *P. aeruginosa* by using a functional T6SS and the Tde toxins, whereas the Tae toxin does not seem to act as a potent effector in this context. All together, the Tde DNase toxins may be pivotal antibacterial toxins that *A. tumefaciens* uses against competitors during in planta colonization, as shown by the different competition scenarios illustrated in Figure 6.

The Tde-Tdi Couple Is Conserved among Bacterial Species

The identification of Tde toxins and the characterization of their role in plant colonization by *A. tumefaciens* prompted us to explore whether the Tde family is prevalent in plant-associated bacteria. The results obtained by BLASTP sequence homology search and the information extracted from the Pfam database highlighted the conservation of Tde-like proteins harboring the putative toxin_43 domain across several bacterial phyla (Figure 7A). The Tde-like superfamily can be divided into eight classes depending on the domain organization of the protein, ranging from a single (class 1) or tandem toxin_43 domains (class 2) to fusion with other domains with known or yet-to-be-identified functions (classes 3 to 8) (Figure 7B). Tde1 belongs to class 1, the most frequent, and contains only an identifiable C-terminal toxin_43 domain. Tde2 falls in class 3 and displays a domain of unknown function, DUF4150, within its N-terminal region. According to the Pfam database, this domain shows similarity to the recently characterized proline-alanine-alanine-arginine (PAAR) domain (Shneider et al., 2013), which can also be found in class 7. A direct sequence alignment between DUF4150 and PAAR motif-containing proteins revealed significant conservation between the two domains and suggests that DUF4150 could act as a PAAR-like protein (Figure S6).

The immunity proteins Tdi1 and Tdi2 contain an uncharacterized GAD-like and DUF1851 domains, which are well-conserved features in other putative Tdi homologs (Figure S3). Notably, the *tde-tdi* gene pair is conserved in Gram-negative Proteobacteria harboring T6SS features and highly prevalent in a wide range of plant pathogens (e.g., *Pseudomonas syringae* pv. syringae, *Pseudomonas syringae* pv. tomato), symbionts (e.g., *Rhizobium leguminosarum*), and plant growth-promoting bacteria (e.g., *Pseudomonas putida*), which further suggests their potential role for colonization in planta. The *tde-tdi* gene pair is also found in T6SS-negative organisms including Gram-positive Firmicutes (e.g., *Bacillus cereus*, *Staphylococcus epidermidis*) and Actinobacteria (e.g., *Mycobacterium abscessus*) as well as Gram-negative Bacteroidetes (e.g., *Bacteroides vulgatus*) (Figure 7A). This observation would imply the presence of alternative secretion mechanisms for Tde transport or other functions yet to be identified in this subset of microorganisms.

DISCUSSION

In a form of bacterial warfare involving the T6SS nanomachine, peptidoglycan (English et al., 2012; Russell et al., 2011, 2012) and membrane lipids (Russell et al., 2013) were shown to be the main targets for T6SS toxins. Our discovery of a superfamily of DNases (Tde), together with the recently identified VgrG-dependent Rhs DNases (Koskiniemi et al., 2013) and predicted polymorphic nuclease toxins (Zhang et al., 2012), expands the repertoire of characterized T6SS-dependent antibacterial toxins. The Tde DNase toxins identified in this present study do not share homology with Rhs or any other characterized bacterial DNases (Figure S7), which suggests a unique biochemical activity for the Tde toxins.

The widespread presence of *tde-tdi* couples in divergent bacterial phyla reveals the conservation of this family of toxin-immunity pairs. The presence of a genetic linkage between *vgrG* and *tde-tdi* genes in most analyzed Proteobacteria agrees with previous observations that *vgrG* genes are often linked to genes encoding toxins (Koskiniemi et al., 2013; Russell et al., 2013). Two recent reports further demonstrated the requirement of the cognate VgrG for specific toxin-mediated antibacterial activity (Hachani et al., 2014; Whitney et al., 2014). Considering

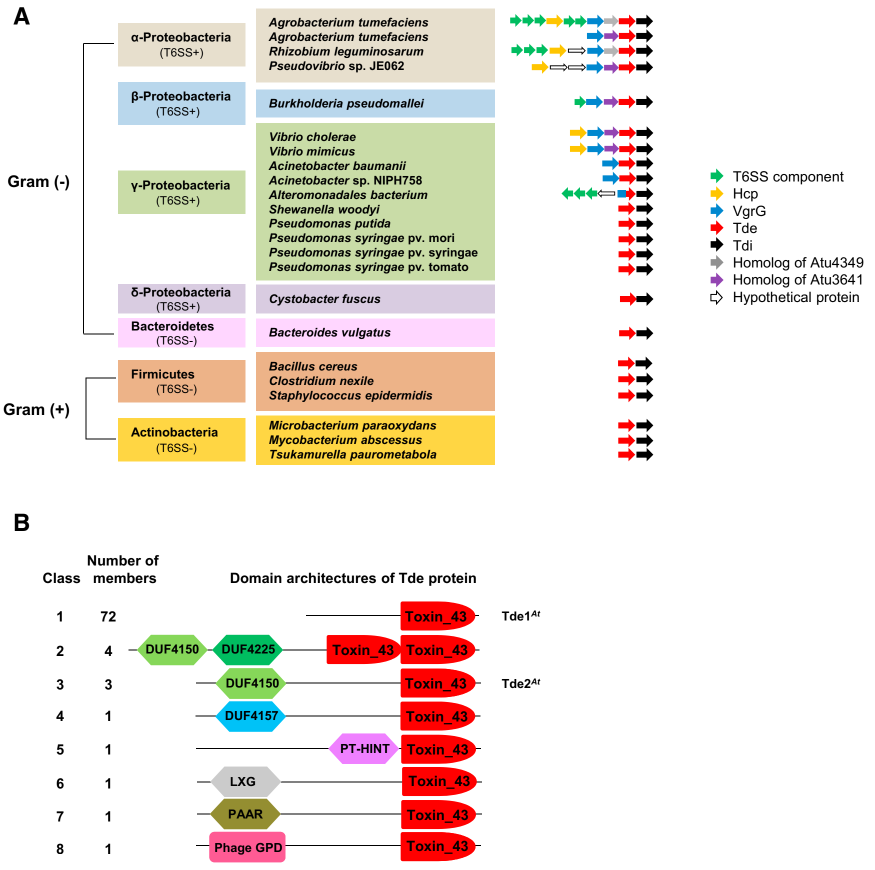

Figure 7. Conservation of Tde-Tdi Families in Bacteria

(A) Representatives of the Tde family (shown in Figure 2A) from Gram (−) Proteobacteria and Bacteroidetes and Gram (+) Firmicutes and Actinobacteria phyla. The genetic organization is deducted from the genome context survey by BLASTP analysis and homologous genes are color-coded according to their known or predicted functions. The presence (indicated as T6SS+) or absence of T6SS (indicated as T6SS−) is based on the BLASTP analysis of the conserved T6SS components TssM, TssB, VgrG, and Hcp.

(B) Eight classes of toxin_43 superfamily (PF15604). Proteins containing the toxin_43 domains are classified into eight classes/architectures according to the Pfam database. The graphical domain composition shows distinct domain organizations from a single to tandem toxin_43 domain fused to domains with known or unknown functions. The number of protein members found in each class is shown and classification of Tde1^{At} (*A. tumefaciens* Tde1) as class 1 and Tde2^{At} (*A. tumefaciens* Tde2) as class 3 is indicated. Detailed information for all class members and domain descriptions can be found in the Pfam PF15604 database (http://pfam.xfam.org/family/toxin_43). See also Figures S1, S3, S6, and S7.

the genetic linkage between *vgrG1* and *tde1-tdi1* or *vgrG2* and *tde2-tdi2* in *A. tumefaciens*, VgrG1 and VgrG2 may bind specifically to Tde1 and Tde2, respectively, either directly or indirectly, to facilitate their secretion and delivery in the target cells.

Interestingly, the domain modularity observable in the Tde superfamily further supports the use of distinct transport mechanisms for each Tde class, as was generally suggested for the T6SS (Shneider et al., 2013). For example, Tde1 contains only a recognizable C-terminal toxin_43 domain, whereas Tde2 contains an additional N-terminal DUF4150 domain that shares sequence similarity with PAAR motif-containing proteins. This PAAR superfamily of proteins was recently described to sharpen the VgrG spike and to act as an adaptor to facilitate T6SS-mediated secretion of a broad range of toxins (Filloux, 2013; Shneider et al., 2013). Thus, the DUF4150 motif within the Tde2 toxin may be required to adapt or connect the protein at the tip of a VgrG spike to allow for delivery. The DUF4150 domain is also found in class 2 Tde toxins and can have a similar function for this subclass of proteins. Additional adaptor domains including known PAAR domain and other uncharacterized domains located at the N-terminal sequence of different Tde subclasses may be candidates for this function. In contrast, independent adaptor domains could be involved, as it would be the case for Tde1, which does not display any recognizable domain at its N terminus.

Of note, the importance of the T6SS and its associated toxins varies substantially depending on which set of bacteria are placed in competition and whether this occurs during in vitro or in vivo situations. Our findings that *A. tumefaciens* was outcompeted by *P. aeruginosa* in vitro is consistent with previous observation for significant competitive advantage of *P. aeruginosa* over *A. tumefaciens* in both planktonic and biofilm growth (An et al., 2006). The mechanisms for the domination of *P. aeruginosa* involve a faster growth rate, motility, and an unknown compound(s) capable of dispersal and inhibition of *A. tumefaciens* biofilm (An et al., 2006; Hibbing and Fuqua, 2012). Interestingly, in addition to its obvious growth advantage over *A. tumefaciens* under laboratory growth conditions, *P. aeruginosa* further triggers a lethal counterattack against T6SS-active *A. tumefaciens*. This phenomenon is clearly reminiscent of the recently described T6SS-dueling behavior (Basler and Mekalanos, 2012), with *P. aeruginosa* using a "tit-for-tat" strategy to counterattack threatening cells such as *Vibrio cholerae* or *Acinetobacter baylyi* (Basler et al., 2013). In regards to the *A. tumefaciens*-*P. aeruginosa* competition in vitro, the danger signal sensed by *P. aeruginosa* may be represented by the injected toxin and not the T6SS machinery itself. *P. aeruginosa* was recently found to induce a lethal T6SS counterattack in response to the T4SS mating system (Ho et al., 2013). In our study, the "T6SS counter-attack" trigger was not restricted to the Tde injection but was also effective with the injection of Tae, which alters the integrity of the bacterial cell envelope. Thus, the *P. aeruginosa* T6SS response may result from sensing a wide variety of cellular perturbation, including DNA damage or membrane/cell wall damage.

The competition outcomes and the relationship between *A. tumefaciens* and *P. aeruginosa* appear to vary greatly when switching from an in vitro to an in vivo environmental context.

Inside the host plant, *A. tumefaciens* exhibits the T6SS- and Tde-dependent competitive advantage over *P. aeruginosa*, which suggests that the plant environment is a crucial determinant for the selection of the fittest *A. tumefaciens* strains. It is also striking that this competitive advantage for *A. tumefaciens* during intraspecies interaction is only observed in planta but not during in vitro growth, even though both antibacterial activity and type VI secretion were readily detected in vitro. While the molecular mechanisms and biological significance underlying this observation await future investigation, we speculated that *A. tumefaciens* may be able to recognize *Agrobacterium* or Rhizobiaceae-specific components that are absent in other distantly related bacteria such as *E. coli* and *P. aeruginosa* and choose not to attack its own siblings during free-living environment. Once *A. tumefaciens* successfully infects the host plant, *A. tumefaciens* may adjust its antibacterial stragtegy to attack all other nonisogenic bacteria at both intraspecies and interspecies levels, aiming to secure the nutrient for its own replication in the apoplast. It is worth mentioning that *Agrobacterium* T6SS may be also regulated by nutrients as type VI secretion is active in neutral rich medium 523 (Wu et al., 2008) or LB (Figure S5C) but not in minimal AB-MES medium (pH 7.0) (Wu et al., 2012). Thus, *A. tumefaciens* seems to regulate T6SS activity at multiple levels with complex mechanisms in response to different environmental cues. Therefore, beyond acidity (Wu et al., 2012), additional plant signal(s) may be required to trigger the ability of *A. tumefaciens* in differentiating self from nonself in order to attack coexisting competitors in the same ecological niche. Recent findings for a role of T6SS in export of self-identity proteins to provide a competitive advantage and territoriality in the social bacterium *Proteus mirabilis* indeed support the importance of self-recognition in interbacterial interactions (Alteri et al., 2013; Wenren et al., 2013).

The use of Tde as an antibacterial toxin to increase the fitness of *A. tumefaciens* during plant colonization lends support to their key role in a physiological and ecological context. This finding presents an unprecedented role of T6SS effector activity for bacterial competitive advantage at both intraspecies and interspecies levels inside a plant host. The distribution of tandem *tde-tdi* genes in the genomes of plant-associated bacteria suggests the conservation of this mechanism among other phytobacteria. Similar benefits were observed in the human pathogen *V. cholerae* during colonization of the infant rabbit intestine (Fu et al., 2013). Whereas *A. tumefaciens* uses the Tde DNases as major weapons to attack both its own siblings and *P. aeruginosa* during in planta colonization, *V. cholerae* delivers VgrG3 to target peptidoglycan of competing siblings for survival inside the animal host. In both cases, the cognate immunity is essential for this in vivo competitive advantage and sufficient to protect the toxin-producing bacterium from killing. In conclusion, the in vivo fitness advantage conferred by the T6SS for both plant and animal pathogens offers a unique perspective in the evaluation of the T6SS in the host, particularly within a polymicrobial environment.

EXPERIMENTAL PROCEDURES

Bacterial Strains and Plasmids

Strains, plasmids, and primer sequences used in this study are in Tables S1 and S2. *E. coli* and *P. aeruginosa* strains were grown in LB, whereas 523 medium (Kado and Heskett, 1970) was routinely used for *A. tumefaciens* strains unless indicated. Growth conditions and mutant construction are as previously described (Lossi et al., 2013; Ma et al., 2009).

Bioinformatics Analysis

All sequences identified in this study were obtained from the NCBI database (http://www.ncbi.nlm.nih.gov/). Tde family proteins were identified by a BLASTP search with the amino acid sequence of the toxin_43 domain (defined by Pfam database, http://pfam.xfam.org/) for Tde1 (Atu4350) and Tde2 (Atu3640) against the non-redundant protein database to identify the Tde homologs with E value < 10^{-4} and extracted from the Pfam toxin_43 (PF15604) database. The Tde family was aligned by use of ClustalW on EMBL-EBI website (http://www.ebi.ac.uk/), and the secondary structure for the Tde1 toxin_43 domain was predicted by using the PSIPRED server (http://bioinf.cs.ucl.ac.uk/psipred/). Sequence logos were generated manually by examining the genome context of the neighbor genes. The presence of a signal peptide was predicted by using SignalP (http://www.cbs.dtu.dk/services/SignalP/).

In Vitro DNase Activity Assay

Plasmid DNA of pTrc200 (1 μg) was incubated with purified C-terminal His-tagged Tde1 or Tde1 derivative (H190A D193A) (0.5 μg) in 15 μl of 10 mM Tris/HCl (pH 7.5) for 1 hr at 37°C in the presence or absence of 2 mM Mg^{2+}. Plasmid DNA with sample buffer served as a control. The integrity of DNA was visualized on 1% agarose gel. Tde proteins were overexpressed and purified from *E. coli* by nickel chromatography with details described in Supplemental Experimental Procedures.

Plasmid DNA Degradation Analysis in *E. coli* Cells

Overnight cultures of *E. coli* DH10B strain harboring the empty vectors or derivatives expressing Tde toxins were harvested and adjusted to an OD_{600} 0.3 containing 0.2% L-arabinose for a further 2 hr to produce Tde toxins. Equal cell mass was collected, and plasmid DNA was extracted within an equal volume for DNA gel analysis.

Secretion Assay

Secretion assay from liquid culture was performed in LB or AB-MES for 4–6 hr at 25°C, as previously described (Ma et al., 2009). For detecting secretion on agar plate, *A. tumefaciens* cells were grown in liquid 523 for 16 hr at 28°C. The harvested cells were adjusted to OD_{600} 1 with AB-MES (pH 5.5) (Lai and Kado, 1998), and 100 μl of cell suspension was spread and incubated on an AB-MES (pH 5.5) agar plate for 24 hr at 25°C. Cells were collected in 5 ml AB-MES (pH 5.5) and secreted protein was analyzed as described (Ma et al., 2009).

Growth Inhibition Assay

Overnight cultures of *E. coli* DH10B strain harboring vectors or their derivatives were adjusted to OD_{600} 0.1. Expression of the tested immunity protein was induced by 1 mM IPTG for 1 hr before L-arabinose (0.2% final concentration) was added to induce expression of the toxin. For growth inhibition assay with *A. tumefaciens*, overnight cultures of *A. tumefaciens* C58 strain harboring empty vectors or their derivatives were adjusted to OD_{600} 0.1. The tested immunity protein was constitutively expressed, and the toxin protein was induced with 1 mM IPTG. The growth was monitored by measuring OD_{600} at 1 hr intervals.

Interbacterial Competition Assay

The in planta competition assay was carried out by infiltration of bacterial cells into leaves of *Nicotiana benthamiana*, and the bacterial cell number was counted after 24 hr incubation at room temperature. Interbacterial competition assay on agar plate was performed by coculture on LB (pH 7.0) or AB-MES (pH 5.5) agar at 25°C for 16 hr. The competition outcome was quantified by counting colony forming units (CFU) on selective LB agar. All assays were performed with at least three independent experiments or a minimum of three biological replicates from two independent experiments. Data represent mean ±SE of all biological replicates. Statistics were calculated by Student's t test, and the p value was denoted as *** = $p < 0.0005$, ** = $p < 0.005$, and * = $p < 0.05$. Detailed methods and associated references are described in Supplemental Experimental Procedures.

TUNEL and Fluorescence-Activated Cell Sorting Analysis

Overnight culture of *E. coli* DH10B strains harboring the pJN105 vector or derivatives expressing Tde toxins were harvested, fixed, and stained by Apo-Direct Kit (BD Bioscience), and the intensity of fluorescence was determined by MoFlo XDP Cell Sorter (Beckman Coulter) and Summit V 5.2 software. Detailed methods and associated references are described in Supplemental Experimental Procedures.

SUPPLEMENTAL INFORMATION

Supplemental Information includes seven figures, two tables, and Supplemental Experimental Procedures and can be found with this article online at http://dx.doi.org/10.1016/j.chom.2014.06.002.

ACKNOWLEDGMENTS

The authors acknowledge Fred Ausubel, Jen Sheen, Chih-Horng Kuo, and Hanna Yuan for critical reading of this manuscript and the members of the Lai and Filloux laboratories for discussion. We also thank the technical assistance of Wen-Ching Lin with the secretion assay and the Flow Cytometry Core Facility and DNA Sequencing Laboratories at the Institute of Plant and Microbial Biology, Academia Sinica, for fluorescence-activated cell sorting analysis and DNA sequencing, respectively. This work was supported by the 2011 Taiwan Initiative Research Cooperation among Top Universities between UK and Taiwan from the National Science Council (NSC 100-2911-I-001-038) to E.M.L. and A.F., research grants from the National Science Council (NSC 98-2311-B-001 -002 -MY3 and NSC 101-2321-B-001 -033 -) to E.M.L., and the Medical Research Council grant MR/K001930/1 and the Wellcome Trust grant WT091939 to A.F. L.S.M. received postdoctoral fellowships from the National Science Council (NSC 100-2911-I-001-038) and Academia Sinica. J.S.L. received a postdoctoral fellowship from the National Science Council (101-2321-B-001 -033 -).

Received: March 17, 2014
Revised: May 6, 2014
Accepted: May 27, 2014
Published: June 26, 2014

REFERENCES

Alteri, C.J., Himpsl, S.D., Pickens, S.R., Lindner, J.R., Zora, J.S., Miller, J.E., Arno, P.D., Straight, S.W., and Mobley, H.L. (2013). Multicellular bacteria deploy the type VI secretion system to preemptively strike neighboring cells. PLoS Pathog. *9*, e1003608.

Alvarez-Martinez, C.E., and Christie, P.J. (2009). Biological diversity of prokaryotic type IV secretion systems. Microbiol. Mol. Biol. Rev. *73*, 775–808.

An, D., Danhorn, T., Fuqua, C., and Parsek, M.R. (2006). Quorum sensing and motility mediate interactions between Pseudomonas aeruginosa and Agrobacterium tumefaciens in biofilm cocultures. Proc. Natl. Acad. Sci. USA *103*, 3828–3833.

Anand, A., Vaghchhipawala, Z., Ryu, C.M., Kang, L., Wang, K., del-Pozo, O., Martin, G.B., and Mysore, K.S. (2007). Identification and characterization of plant genes involved in Agrobacterium-mediated plant transformation by virus-induced gene silencing. Mol. Plant Microbe Interact. *20*, 41–52.

Basler, M., and Mekalanos, J.J. (2012). Type 6 secretion dynamics within and between bacterial cells. Science *337*, 815.

Basler, M., Pilhofer, M., Henderson, G.P., Jensen, G.J., and Mekalanos, J.J. (2012). Type VI secretion requires a dynamic contractile phage tail-like structure. Nature *483*, 182–186.

Basler, M., Ho, B.T., and Mekalanos, J.J. (2013). Tit-for-tat: type VI secretion system counterattack during bacterial cell-cell interactions. Cell *152*, 884–894.

Benz, J., and Meinhart, A. (2014). Antibacterial effector/immunity systems: it's just the tip of the iceberg. Curr. Opin. Microbiol. *17*, 1–10.

Bönemann, G., Pietrosiuk, A., and Mogk, A. (2010). Tubules and donuts: a type VI secretion story. Mol. Microbiol. *76*, 815–821.

Braun, V., and Patzer, S.I. (2013). Intercellular communication by related bacterial protein toxins: colicins, contact-dependent inhibitors, and proteins exported by the type VI secretion system. FEMS Microbiol. Lett. *345*, 13–21.

Brooks, T.M., Unterweger, D., Bachmann, V., Kostiuk, B., and Pukatzki, S. (2013). Lytic activity of the Vibrio cholerae type VI secretion toxin VgrG-3 is inhibited by the antitoxin TsaB. J. Biol. Chem. *288*, 7618–7625.

Brunet, Y.R., Hénin, J., Celia, H., and Cascales, E. (2014). Type VI secretion and bacteriophage tail tubes share a common assembly pathway. EMBO Rep. *15*, 315–321.

Cascales, E. (2008). The type VI secretion toolkit. EMBO Rep. *9*, 735–741.

Dong, T.G., Ho, B.T., Yoder-Himes, D.R., and Mekalanos, J.J. (2013). Identification of T6SS-dependent effector and immunity proteins by Tn-seq in Vibrio cholerae. Proc. Natl. Acad. Sci. USA *110*, 2623–2628.

English, G., Trunk, K., Rao, V.A., Srikannathasan, V., Hunter, W.N., and Coulthurst, S.J. (2012). New secreted toxins and immunity proteins encoded within the Type VI secretion system gene cluster of Serratia marcescens. Mol. Microbiol. *86*, 921–936.

Filloux, A. (2013). Microbiology: a weapon for bacterial warfare. Nature *500*, 284–285.

Filloux, A., Hachani, A., and Bleves, S. (2008). The bacterial type VI secretion machine: yet another player for protein transport across membranes. Microbiology *154*, 1570–1583.

Fu, Y., Waldor, M.K., and Mekalanos, J.J. (2013). Tn-Seq analysis of vibrio cholerae intestinal colonization reveals a role for T6SS-mediated antibacterial activity in the host. Cell Host Microbe *14*, 652–663.

Gelvin, S.B. (2010). Plant proteins involved in Agrobacterium-mediated genetic transformation. Annu. Rev. Phytopathol. *48*, 45–68.

Hachani, A., Lossi, N.S., Hamilton, A., Jones, C., Bleves, S., Albesa-Jové, D., and Filloux, A. (2011). Type VI secretion system in Pseudomonas aeruginosa: secretion and multimerization of VgrG proteins. J. Biol. Chem. *286*, 12317–12327.

Hachani, A., Lossi, N.S., and Filloux, A. (2013). A visual assay to monitor T6SS-mediated bacterial competition. J. Vis. Exp. *73*, e50103.

Hachani, A., Allsopp, L.P., Oduko, Y., and Filloux, A. (2014). The VgrG proteins are "A la carte" delivery systems for bacterial type VI effectors. J. Biol. Chem. http://dx.doi.org/10.1074/jbc.M114.563429.

Hibbing, M.E., and Fuqua, C. (2012). Inhibition and dispersal of Agrobacterium tumefaciens biofilms by a small diffusible Pseudomonas aeruginosa exoproduct(s). Arch. Microbiol. *194*, 391–403.

Ho, B.T., Basler, M., and Mekalanos, J.J. (2013). Type 6 secretion system-mediated immunity to type 4 secretion system-mediated gene transfer. Science *342*, 250–253.

Ho, B.T., Dong, T.G., and Mekalanos, J.J. (2014). A view to a kill: the bacterial type VI secretion system. Cell Host Microbe *15*, 9–21.

Hood, R.D., Singh, P., Hsu, F., Güvener, T., Carl, M.A., Trinidad, R.R., Silverman, J.M., Ohlson, B.B., Hicks, K.G., Plemel, R.L., et al. (2010). A type VI secretion system of Pseudomonas aeruginosa targets a toxin to bacteria. Cell Host Microbe *7*, 25–37.

Hu, J.Y., Fan, Y., Lin, Y.H., Zhang, H.B., Ong, S.L., Dong, N., Xu, J.L., Ng, W.J., and Zhang, L.H. (2003). Microbial diversity and prevalence of virulent pathogens in biofilms developed in a water reclamation system. Res. Microbiol. *154*, 623–629.

Kado, C.I., and Heskett, M.G. (1970). Selective media for isolation of Agrobacterium, Carynebacterium, Erwinia, Pseudomonas, and Xanthomonas. Phytopathology *60*, 969–976.

Kapitein, N., Bönemann, G., Pietrosiuk, A., Seyffer, F., Hausser, I., Locker, J.K., and Mogk, A. (2013). ClpV recycles VipA/VipB tubules and prevents non-productive tubule formation to ensure efficient type VI protein secretion. Mol. Microbiol. *87*, 1013–1028.

Koskiniemi, S., Lamoureux, J.G., Nikolakakis, K.C., t'Kint de Roodenbeke, C., Kaplan, M.D., Low, D.A., and Hayes, C.S. (2013). Rhs proteins from diverse bacteria mediate intercellular competition. Proc. Natl. Acad. Sci. USA *110*, 7032–7037.

Lai, E.M., and Kado, C.I. (1998). Processed VirB2 is the major subunit of the promiscuous pilus of Agrobacterium tumefaciens. J. Bacteriol. *180*, 2711–2717.

Leiman, P.G., Basler, M., Ramagopal, U.A., Bonanno, J.B., Sauder, J.M., Pukatzki, S., Burley, S.K., Almo, S.C., and Mekalanos, J.J. (2009). Type VI secretion apparatus and phage tail-associated protein complexes share a common evolutionary origin. Proc. Natl. Acad. Sci. USA *106*, 4154–4159.

LeRoux, M., De Leon, J.A., Kuwada, N.J., Russell, A.B., Pinto-Santini, D., Hood, R.D., Agnello, D.M., Robertson, S.M., Wiggins, P.A., and Mougous, J.D. (2012). Quantitative single-cell characterization of bacterial interactions reveals type VI secretion is a double-edged sword. Proc. Natl. Acad. Sci. USA *109*, 19804–19809.

Lin, J.S., Ma, L.S., and Lai, E.M. (2013). Systematic dissection of the agrobacterium type VI secretion system reveals machinery and secreted components for subcomplex formation. PLoS ONE *8*, e67647.

Lin, J.S., Wu, H.H., Hsu, P.H., Ma, L.S., Pang, Y.Y., Tsai, M.D., and Lai, E.M. (2014). Fha interaction with phosphothreonine of TssL activates type VI secretion in Agrobacterium tumefaciens. PLoS Pathog. *10*, e1003991.

Liu, A.C., Shih, H.W., Hsu, T., and Lai, E.M. (2008). A citrate-inducible gene, encoding a putative tricarboxylate transporter, is downregulated by the organic solvent DMSO in Agrobacterium tumefaciens. J. Appl. Microbiol. *105*, 1372–1383.

Lossi, N.S., Manoli, E., Förster, A., Dajani, R., Pape, T., Freemont, P., and Filloux, A. (2013). The HsiB1C1 (TssB-TssC) complex of the Pseudomonas aeruginosa type VI secretion system forms a bacteriophage tail sheathlike structure. J. Biol. Chem. *288*, 7536–7548.

Ma, L.S., Lin, J.S., and Lai, E.M. (2009). An IcmF family protein, $ImpL_M$, is an integral inner membrane protein interacting with $ImpK_L$, and its walker a motif is required for type VI secretion system-mediated Hcp secretion in Agrobacterium tumefaciens. J. Bacteriol. *191*, 4316–4329.

Ma, L.S., Narberhaus, F., and Lai, E.M. (2012). IcmF family protein TssM exhibits ATPase activity and energizes type VI secretion. J. Biol. Chem. *287*, 15610–15621.

Pukatzki, S., Ma, A.T., Revel, A.T., Sturtevant, D., and Mekalanos, J.J. (2007). Type VI secretion system translocates a phage tail spike-like protein into target cells where it cross-links actin. Proc. Natl. Acad. Sci. USA *104*, 15508–15513.

Rahme, L.G., Stevens, E.J., Wolfort, S.F., Shao, J., Tompkins, R.G., and Ausubel, F.M. (1995). Common virulence factors for bacterial pathogenicity in plants and animals. Science *268*, 1899–1902.

Russell, A.B., Hood, R.D., Bui, N.K., LeRoux, M., Vollmer, W., and Mougous, J.D. (2011). Type VI secretion delivers bacteriolytic effectors to target cells. Nature *475*, 343–347.

Russell, A.B., Singh, P., Brittnacher, M., Bui, N.K., Hood, R.D., Carl, M.A., Agnello, D.M., Schwarz, S., Goodlett, D.R., Vollmer, W., and Mougous, J.D. (2012). A widespread bacterial type VI secretion effector superfamily identified using a heuristic approach. Cell Host Microbe *11*, 538–549.

Russell, A.B., LeRoux, M., Hathazi, K., Agnello, D.M., Ishikawa, T., Wiggins, P.A., Wai, S.N., and Mougous, J.D. (2013). Diverse type VI secretion phospholipases are functionally plastic antibacterial effectors. Nature *496*, 508–512.

Russell, A.B., Peterson, S.B., and Mougous, J.D. (2014). Type VI secretion system effectors: poisons with a purpose. Nat. Rev. Microbiol. *12*, 137–148.

Schmeisser, C., Stöckigt, C., Raasch, C., Wingender, J., Timmis, K.N., Wenderoth, D.F., Flemming, H.C., Liesegang, H., Schmitz, R.A., Jaeger, K.E., and Streit, W.R. (2003). Metagenome survey of biofilms in drinking-water networks. Appl. Environ. Microbiol. *69*, 7298–7309.

Shalom, G., Shaw, J.G., and Thomas, M.S. (2007). In vivo expression technology identifies a type VI secretion system locus in Burkholderia pseudomallei that is induced upon invasion of macrophages. Microbiology *153*, 2689–2699.

Shneider, M.M., Buth, S.A., Ho, B.T., Basler, M., Mekalanos, J.J., and Leiman, P.G. (2013). PAAR-repeat proteins sharpen and diversify the type VI secretion system spike. Nature *500*, 350–353.

Troxler, J., Azelvandre, P., Zala, M., Defago, G., and Haas, D. (1997). Conjugative transfer of chromosomal genes between fluorescent Pseudomonads in the rhizosphere of wheat. Appl. Environ. Microbiol. *63*, 213–219.

Wenren, L.M., Sullivan, N.L., Cardarelli, L., Septer, A.N., and Gibbs, K.A. (2013). Two independent pathways for self-recognition in Proteus mirabilis are linked by type VI-dependent export. MBio. *4*, e00374–13.

Whitney, J.C., Chou, S., Russell, A.B., Biboy, J., Gardiner, T.E., Ferrin, M.A., Brittnacher, M., Vollmer, W., and Mougous, J.D. (2013). Identification, structure, and function of a novel type VI secretion peptidoglycan glycoside hydrolase effector-immunity pair. J. Biol. Chem. *288*, 26616–26624.

Whitney, J.C., Beck, C.M., Goo, Y.A., Russell, A.B., Harding, B.N., De Leon, J.A., Cunningham, D.A., Tran, B.Q., Low, D.A., Goodlett, D.R., et al. (2014). Genetically distinct pathways guide effector export through the type VI secretion system. Mol. Microbiol. *92*, 529–542.

Wu, H.Y., Chung, P.C., Shih, H.W., Wen, S.R., and Lai, E.M. (2008). Secretome analysis uncovers an Hcp-family protein secreted via a type VI secretion system in Agrobacterium tumefaciens. J. Bacteriol. *190*, 2841–2850.

Wu, C.F., Lin, J.S., Shaw, G.C., and Lai, E.M. (2012). Acid-induced type VI secretion system is regulated by ExoR-ChvG/ChvI signaling cascade in Agrobacterium tumefaciens. PLoS Pathog. *8*, e1002938.

Zhang, D., de Souza, R.F., Anantharaman, V., Iyer, L.M., and Aravind, L. (2012). Polymorphic toxin systems: Comprehensive characterization of trafficking modes, processing, mechanisms of action, immunity and ecology using comparative genomics. Biol. Direct *7*, 18.

Cell Host & Microbe, Volume *16*

Supplemental Information

Agrobacterium tumefaciens Deploys a Superfamily of Type VI Secretion DNase Effectors as Weapons for Interbacterial Competition In Planta

Lay-Sun Ma, Abderrahman Hachani, Jer-Sheng Lin, Alain Filloux, and Erh-Min Lai

Cell Host & Microbe, Volume *16*

Supplemental Information

Agrobacterium tumefaciens Deploys a Superfamily of Type VI Secretion DNase Effectors As Weapons for Interbacterial Competition *In Planta*

Lay-Sun Ma, Abderrahman Hachani, Jer-Sheng Lin, Alain Filloux, and Erh-Min Lai

TABLE S1. Bacterial strains and plasmids, related to Experimental Procedures.

Strain /plasmid	Relevant characteristics	Source/Ref.
A. tumefaciens		
C58	Wild type virulent strain containing nopaline-type Ti plasmid pTiC58	Eugene Nester
ΔT6SS	Deletion of both *t6ss* promoter (Δ*pro*) and *vgrG2* operon	This study
Δ*tssL*	*tssL* deletion mutant	(Ma et al., 2009)
Δ*tae-tai*	Deletion from *atu4346* to *atu4347*	(Lin et al., 2013)
Δ*tde1-tdi1*	Deletion from *atu4350* to *atu4351*	This study
Δ*4349-tde1-tdi1*	Deletion from *atu4349* to *atu4351*	This study
Δ*tde2-tdi2*	Deletion from *atu3639 to atu3640*	This study
Δ3TIs	Deletion from *atu3639 to atu3640*, *atu4350* to *atu4351,* and *atu4346* to *atu4347*	This study
Δ*tde1-tdi1* Δ*tde2-tdi2*	Deletion from *atu3639 to atu3640* and from *atu4350* to *atu4351*	This study
P. aeruginosa		
PAK	Wild type *P. aeruginosa*	A. Filloux
Δ*retS*	In-frame deletion of *retS* (PA4856) in PAK	(Goodman et al., 2004)
Δ*retS*ΔH1	H1-T6SS cluster deletion in Δ*retS*	(Hachani et al., 2013)
E. coli		
Top10	Host for DNA cloning	Invitrogen
BL21(DE3)	Host for overexpressing genes driven by the T7 promoter	(Studier et al., 1990)
**Plasmids**		
pRL662	Gm^R, broad host range vector derived from pBBR1MCS-2	(Vergunst et al., 2000)
pET22b(+)	Ap^R, *E. coli* overexpression vector to produce C-terminal His-tagged protein	Novagen
pET28a(+)	Km^R, *E. coli* overexpression vector to produce N or C-terminal His-tagged protein	Novagen
pJQ200KS	Gm^R, suicide plasmid containing Gm^r and *sacB* gene for double crossover event selection	(Quandt and Hynes, 1993)
pTrc200	Sm^R,Sp^R, pVS1 origin *lacI*^{q}, *trc* promoter expression vector	(Schmidt-Eisenlohr et al.,

		1999)
pJN105	Gm^R, arabinose-inducible gene expression vector derived from pBBRMCS-1, *araC*-P_{BAD}	(Newman and Fuqua, 1999)
pJN4347	Gm^R, pJN105 expressing toxin Tae (Atu4347)	This study
pJN4347(ssPelB)	Gm^R, pJN105 expressing toxin Tae (Atu4347) with N-terminal PelB signal peptide (ssPelB)	This study
pJN4350	Gm^R, pJN105 expressing toxin Tde1 (Atu4350)	This study
pJN3640	Gm^R, pJN105 expressing toxin Tde2 (Atu3640)	This study
pTrc4346	Sp^R, pTrc200 expressing immunity protein Tai (Atu4346)	This study
pTrc4351	Sp^R, pTrc200 expressing immunity protein Tdi1 (Atu4351)	This study
pTrc3639	Sp^R, pTrc200 expressing immunity protein Tdi2 (Atu3639)	This study
pTrc4349	Sp^R, pTrc200 expressing Atu4349	This study
pTrc3641	Sp^R, pTrc200 expressing Atu3641	This study
pTrc3640-strep	Sp^R, pTrc200 expressing C-terminal Strep-tagged Tde2 (Atu3640)	This study
pTrc4350-HA	Sp^R, pTrc200 expressing C-terminal HA-tagged Tde1 (Atu4350)	This study
pTrc4349-4352	Sp^R, pTrc200 expressing wild type proteins from Atu4349 to Atu4352	This study
pTrc4349-4352 (H190A D193A)	Sp^R, pTrc200 expressing Atu4349, Tde1 with amino acid substitution (H190A D193A), Tdi1, and Atu4352	This study
pTrc4349-4352 (H190A)	Sp^R, pTrc200 expressing Atu4349, Tde1 with amino acid substitution (H190A), Tdi1, and Atu4352	This study
pTrc4349-4352 (D193A)	Sp^R, pTrc200 expressing Atu4349, Tde1 with amino acid substitution (D193A), Tdi1, and Atu4352	This study
pTssL	Gm^R, pRL662 constitutively expressing TssL (Atu4333)	(Ma et al., 2009)
pRL3639	Gm^R, pRL662 constitutively expressing Tdi2 (Atu3639)	This study
pRL4349	Gm^R, pRL662 constitutively expressing Atu4349	This study
pRL4351-strep	Gm^R, pRL662 constitutively expressing C-terminal Strep-tagged Tdi1 (Atu4351).	This study
pJQ200KS- *pro*	Gm^R, plasmid to generate *t6ss* promoter deletion mutant	(Lin et al., 2013)
pJQ200KS-*vgrG2OP*	Gm^R, plasmid to generate *vgrG2* operon deletion mutant	This study
pJQ200KS-*atu4346-atu4347*	Gm^R, plasmid to generate *atu4346* to *atu4347* deletion mutant	(Lin et al., 2013)
pJQ200KS-*atu3639-atu3640*	Gm^R, plasmid to generate *atu3639* to *atu3640* deletion mutant	This study
pJQ200KS-*atu4350-atu4351*	Gm^R, plasmid to generate *atu4350* to *atu4351* deletion mutant	This study
pJQ200KS-*atu4349-atu4351*	Gm^R, plasmid to generate *atu4349* to *atu4351* deletion mutant	This study

TABLE S2. Primers used in this study, related to Experimental Procedures.

**Plasmids**	**Primer sequence (5 '-3')**[a]
pJN4347 or pJN4347(ssPelB)	CATGCCATGGGCCGCGTTAACTTTGACAC
	TAATACGAGCTCTCAGGACCCGCGGCTGG
pJN4350	CATGCCATGGGCAGTGCGACGACAACTGT
	ATCCGAGCTCTCAAGACACCGGGACGTCA
pJN3640	GGATTCCATATGAGTATCCCTCGCGACAA
	CGGGATCCTACCATTGTCATGTTCCTG
pTrc4346	TATAGGTACCGTTTGCAGCTCACGTCGT
	GCTCTAGACCACTAGTTACTTTTCTGCT
pTrc4351	TATAGGTACCACGGCAATCCTGACGT
	GCTCTAGACTAGCTGCCAATAGTACGA
pTrc3639	TATAGGTACCGATCTTCGACTTTGCCC
	GCTCTAGATTACCTCGCCGAACCGATT
pTrc4349	TAATACGAGCTCAGGTGAAAGTGGCTC
	TATAGGTACCTCATGCGGGCGCTCCGGAT
pTrc3641	CATGCCATGGCGACGGATCATTTTCAG
	TATAGGTACCTCATGCTGCTCCCTTG
pTrc3640-strep	CCGCTCGAGGTACCAAACAACGCTTACCCTG
	GCTCTAGATCACTTTTCGAACTGCGGGTGGCTCCATGTTCCTGT TAATGGCT
pTrc4350-HA	CATGCCATGGTGATCGACCACACCGT
	AAACTGCAGAGACACCGGGACGTCA
pTrc4349-4352	TAATACGAGCTCAGGTGAAAGTGGCTC
	GCTCTAGATGCTGGATATCGTCGT
pTrc4349-4352 (H190A D193A)	TAATACGAGCTCAGGTGAAAGTGGCTC
	ACCAAA**G**CCAAGGTA**GC**GGTTGCGGCCAAC
	AACC**GC**TACCTTGG**C**TTTGGTTGCGGG
	GCTCTAGATGCTGGATATCGTCGT
pTrc4349-4352 (H190A)	TAATACGAGCTCAGGTGAAAGTGGCTC
	CAAATCCAAGGTA**GC**GGTTGCGGCCAA
	TTGGCCGCAACC**GC**TACCTTGGATTTG
	GCTCTAGATGCTGGATATCGTCGT
pTrc4349-4352(D193A)	TAATACGAGCTCAGGTGAAAGTGGCTC
	CCCGCAACCAAA**G**CCAAGGTATGGGT
	ACCCATACCTTGG**C**TTTGGTTGCGGG
	GCTCTAGATGCTGGATATCGTCGT

pRL3639	CCGCTCGAGATCTTCGACTTTGCC
	GCTCTAGATTACCTCGCCGAACCGATT
pRL4349	CCGCTCGAGGTGAAAGTGGCTCCT
	GCTCTAGATCATGCGGGCGCTCCGGAT
pRL4351-strep	TTCCGCTCGAGACGGCAATCCTGACGT
	AATGCGGCCGCTACTTTTCGAACTGCGGGTGGCTCCAGCTGCC AATAGTACGAA
pJQ200KS-*vgrG2OP*	1. GCTCTAGATCGCTGAGTGATCGCCATCG
	2. CGGGATCCATTCATCAGGAACCTCGATAGC
	3. CGGGATCCACGAGATGAGCCACGCCTGTG
	4. TCCCCCCGGGGCAGCAACTCGCCATCAGTG
pJQ200KS-*atu3639-atu3640*	1. GCTCTAGACGTTCATATAGATGTCATT
	2. CGGGATCCTAAGGCATGCGCGTACGG
	3. CGGGATCCACTCATGCTGTCTCCCTTG
	4. AAACTGCAGGAACGACTGGACTGGAAG
pJQ200KS-*atu4350-atu4351*	1. GCTCTAGACAATCCTGACAAGGCCACAGC
	2. CGGGATCCACTCATGCGGGCGCTCCGGA
	3. CGGGATCCAGCTAGAGGGATATTTAAATGG
	4. AACTGCAGGGTGCAGGGCTATATTTATGC
pJQ200KS-*atu4349-atu4351*	1. GCTCTAGAGCATCATGAACACGATCATCG
	2. CGGGATCCGTTCATAATCAAATCCTGACAAAC
	3. CGGGATCCAGCTAGAGGGATATTTAAATGG
	4. AACTGCAGGGTGCAGGGCTATATTTATGC

a: Restriction enzyme sites are underlined, and mutated sequences are indicated by bold type.

Supplementary Figure 1

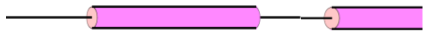

```
                                         CCCCCCCCHHHHHHHHHHHHHHHHC   CCCHHHHHHHH
Atu4350 (15890633)              94 : -PEGATPKQVEEFRRQLKEQQDEIN---RMEPDDLVRNI-------------------------- :  128
Atu3640 (15891300)             345 : FMPTKSTIDIDEFKRQMELQEQGLN---NMSPQQMLANQ-------------------------- :  380
Rleg4DRAFT_5191 (393183392)     93 : -PEGSTPEQIEEFKRQLKEQQDAIN---EIPPDTLVKNM-------------------------- :  127
PJE062_674 (211959488)         288 : PDHLDDDE----FKRQLDEQEDEIN---NTSADKLVERR-------------------------- :  319
BUC_4514 (217394038)           307 : NAKGDPK----EYDRQLLEQEKGLN---DLSVKEYLEGR-------------------------- :  338
VCHE48_1081 (445935354)        354 : KNKQSRKDKAAELDRQLADQQNGLN---NMSVDEYLAGRGAFLGRNPCNPDQKVPKVKRDPIIAK :  415
VMA_001767 (262025037)         354 : KNKQSRKDKAAELDRQLADQQNGLN---NMSVDEYLAGRGAFLGRNPCNPDQKVPKVKRDPIIAK :  415
A1S_0551 (193076348)           318 : KTGKSQAELDAEFDRQLKRQEEGLN---RLTVEEYQQNR-------------------------- :  353
F971_00411 (479947885)         401 : NSLAVEEYLNKETDWQLANQQKGLN---EMSVEDYIQGR-------------------------- :  436
ATW7_01792 (119446764)        1053 : NFKGGKQALEKEFYKQLKAQQAGIN---KMSVGEYLQNRN-----------------------LL : 1091
Swoo_2338 (170726686)          503 : CFKKNKKGDAEEYDRQLKGQQNGLN---DMTVQQYLDNR-------------------------- :  538
Pput_0805 (48546051)           300 : YKIGEFKR-------QLNGQEDGLN---WMTVEEFLEK--------------------------- :  327
PSYMO_00285 (330886100)        303 : DRIPEFDR-------QLAGQEKGLN---DLTVDEYLKGR-------------------------- :  331
PSYR_0686 (66043953)           310 : SKVGEFER-------QLKGQEDGLN---RLTVEEYLKN--------------------------- :  337
PSPTO_2457 (28869652)          342 : MSLVERQKYLKTYSAQLRAQQDAIN---NMTAEEFKSARDSYKN--------------------- :  382
D187_004203 (528053360)          1 : ----------------MRRQQDAIN---EMSVDKFESARKSYKDA-------------------- :   26
C800_03411 (507739969)         194 : KFQNNPHY-EKEMRRQLKMQEDGIN---KLTVFEWLTNR-------------------------- :  228
BCERE0025_58760 (228709322)      3 : -FNRNVKHDSEEFARQLKDQEKGMNELTELTVDEYLKNS-------------------------- :   40
CLONEX_01718 (151383)          377 : VFNYKSKFDEKEFARQLENQEKGLN---SLTIAEFVENR-------------------------- :  412
SEVCU071_1534 (365224737)       38 : SFKRNIKHDEKEFYRQLKNQEQGLN---KLTVKKYMDNR-------------------------- :   63
G362_17760 (516967442)          56 : PFKRNRKHDPVEYDRQYNEQMDALQ---QMPLSDWLRNR-------------------------- :   91
OUW_20586 (382939290)          562 : YTDKHAAEHAAEIDRQTQLWEHGFN---TQSVQQVLDNMD------------------------- :  598
Tpau_0235 (296026115)          536 : AAPTNPTHSPYERIRELLTQITWHN---SQTVQEAYDQA-------------------------- :  571
```

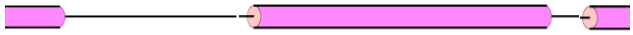

```
                                      HHHHHCCCCCCC           HHHHHHHHHHHHHHHHHHHHHHHHH  HCCCHHHH
Atu4350 (15890633)             129 : -DKYRQQGRPTDD----------AANRRQSREDYRTDRTRELEEKYL--SKGRNDYK-------- :  172
Atu3640 (15891300)             381 : -AKYLANPAGMR------------ALSEPLQAKARQEYRNDPRIQKKYVDQYGPIQG-------- :  424
Rleg4DRAFT_5191 (393183392)    128 : -DTYEDLGRGAKD----------AVDRANAREAWIKNRAAEIVKQDP--STTAKAAR-------- :  171
PJE062_674 (211959488)         320 : --QAIRDAGGTKG-----------VRDHKAQQDAREKHLRDRMLELR--EQQGLSRS-------- :  361
BUC_4514 (217394038)           339 : -ARYQEIGRAGTG---------AAQEQARAKYSRELANQFKDALNEQ--GIFGKAAK-------- :  383
VCHE48_1081 (445935354)        416 : KERIKETKRRQNDKIDDYINNQSLLEDKKAQHGLTSEKAVQKKLKEF--GMEAEAKKRGWSKETI :  478
VMA_001767 (262025037)         416 : KERIKETKRRKKNKIYEYINNQSLLEDKKAQYGLTSEKAVQKKLKEL--GMAETAKKEKWNANEI :  478
A1S_0551 (193076348)           354 : -QLYEKYKRAGTG---------TQQQRIREDMQRQLEESEYKKLKTEQPQLSKGQIER------- :  401
F971_00411 (479947885)         437 : -KAFDTEGRGGGAP----------AEQARKKYGADLAEKYEKEYQQQ--NIGAREAK-------- :  480
ATW7_01792 (119446764)        1092 : TELTDKHGHDKAR---------KILTRNGAAQLSARKELEETIFDSVIDSLERKGILG------D : 1141
Swoo_2338 (170726686)          539 : -KAYNAIGRKGTG-----------AAQKEAREKFRDKLISEYKNDLIDSGEYFGQEA-------- :  583
Pput_0805 (48546051)           328 : -----VENPNQRN--------KGLAQRARKKEQEFIKEKIEKELRK---TMGPFEAQ-------- :  368
PSYMO_00285 (330886100)        332 : --EAFKSGEALRD--------PKVAADARKLLGRKMELNIFRELRLG--GMSPEVAE-------- :  376
PSYR_0686 (66043953)           338 : -----IANPVKRD--------AMAAKKARTDLKDTLQERFQREFQK---EMSPLDAE-------- :  378
PSPTO_2457 (28869652)          383 : ------LGRNPAA--------DAMQRRMGKQMEREVAEKIQKSLVK--KGVDVREAI-------- :  423
D187_004203 (528053360)         27 : -LKATGSGRNPDA--------QRAREVERAAFEEKIRDSLMESMKNDNKGLGYRELK-------- :   74
C800_03411 (507739969)         229 : -KTFREKGR---------------LSSQTAQNDARDAYKRRKMFDYMLLSAENFKYD-------- :  269
BCERE0025_58760 (228709322)     41 : -ERYIAQGRA--------------IEGNAAQQAAREEAYVQKVNELQ---REGLTLS-------- :   79
CLONEX_01718 (151383)          413 : -KKYNVAGRN--------------KKANQFQKELRDKFKQDKIEELF---ENGMSYD-------- :  451
SEVCU071_1534 (365224737)       64 : -EKYIKNGID--------------IEAKHYQMLMREKAIKRKFQELL---SSGVSIK-------- :  102
G362_17760 (516967442)          92 : -TEYLQNGRT--------------PDSLRAQENARAAALKAKILELR---EQGQSRS-------- :  130
OUW_20586 (382939290)          599 : -----ATRPPSTG---------LRDELREGLSDYGYEDLRKAGYGPQQAEQLAKTYAN------- :  642
Tpau_0235 (296026115)          572 : ------KNRTYSS-----------AEAEQARKDFRDQLRTSGYEALLEMGYRPDAADS------- :  612
```

Supplementary Figure 1

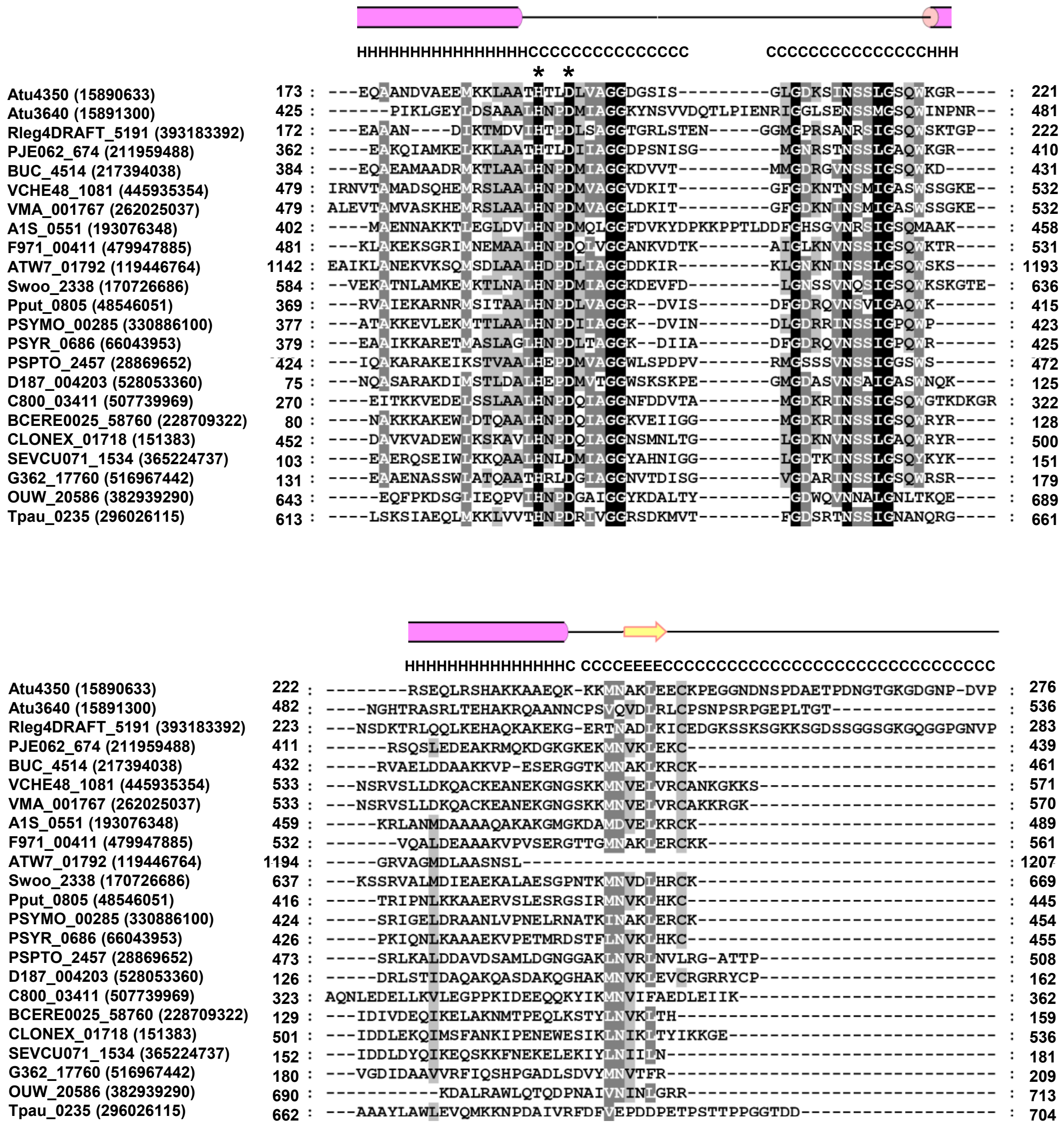

Figure S1. Sequence alignment and secondary structure prediction of the Tde family containing the toxin_43 domains, related to Figure 2A and 7. The sequence alignment of the toxin_43 domain of the representative Tde superfamily proteins generated by use of ClustalW shows the conserved HxxD catalytic motif (* on conserved H and D residues). The amino acid position of residues shown is indicated on each side of the sequences. The locus tag and GI number are shown on the left of each sequence and the conserved amino acid residues are shaded in black for identity and in grey for similarity. Secondary structure of Tde1 was predicted by using the PSIPRED server and is indicated on the top of the sequence alignment. C, coiled-coil; H, alpha-helix; E, beta-sheet.

Supplementary Figure 2

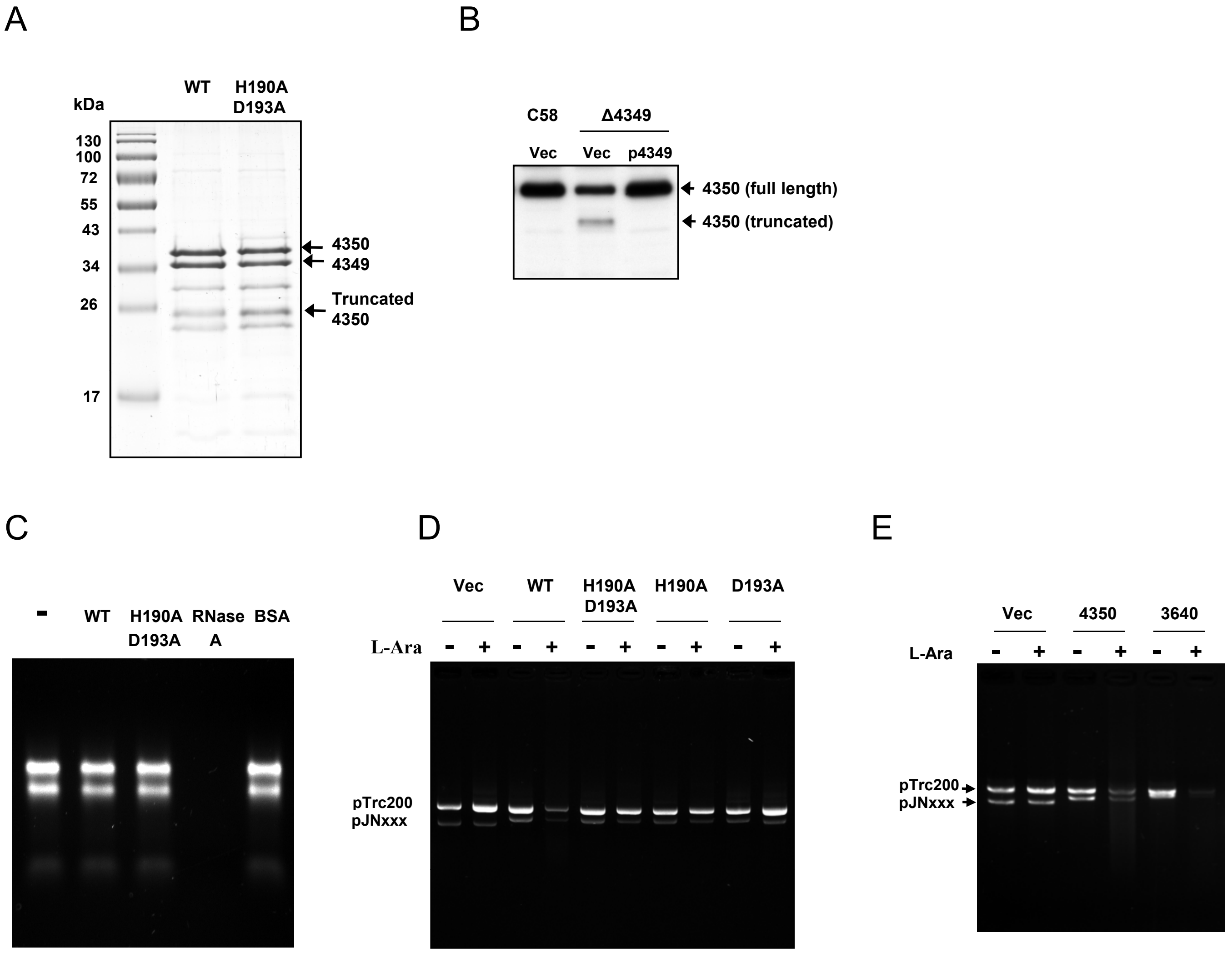

Figure S2. Protein analysis and nuclease activity assay, related to Figure 2. (A) SDS-PAGE analysis of purified wild-type Atu4350 (WT) and its variant (H190A D193A). In total, 2.5 μg of Ni^{2+}-affinity purified His-tagged fusion protein (WT or H190A D193A) co-produced with Atu4349 was loaded in each lane and visualized by Coomassie Blue staining. Full-length Atu4350 and Atu4349 proteins as well as truncated Atu4350 are indicated. Molecular weight standards are shown in kilo daltons on the left. (B) Western blot analysis of Atu4350 stability in *A. tumefaciens* wild-type C58 or the *atu4349* deletion mutant (Δ*4349)* harboring pTrc200 (Vec) or pTrc200 expressing Atu4349 (p4349) grown in AB-MES (pH 5.5) liquid medium. Full-length and truncated Atu4350 proteins are indicated. (C) RNA degradation analysis. An amount of 2 μg of *E. coli* total RNA treated with buffer (-), Atu4350 wild-type (WT), Atu4350 mutant (H190A D193A), RNase A, or BSA was analyzed by RNA-formaldehyde gel. (D) Atu4350 degrades plasmid DNA in *E. coli*. The *E. coli* DH10B cells containing pTrc200 and pJN105 (Vec) or the derivatives expressing wild-type 4350 (WT) or catalytic site mutants (H190A D193A, H190A, D193A) were induced with (+) or without (-) L-arabinose (L-Ara) for 2 hr. An equal volume of plasmids extracted from the same cell mass was analyzed on 1% agarose gel. (E) Atu4350 and Atu3640 degraded plasmid DNA in *E. coli* cells. *E. coli* DH10B cells containing pTrc200 and pJN105 (Vec) or derivatives expressing wild- type Atu4350 or Atu3640 were incubated with (+) or without (-) L-arabinose (L-Ara) for 2 hr. An equal volume of plasmids (pTrc200 and pJNxxx, indicating pJN105 or derivatives) extracted from the same cell mass was analyzed by 1% agarose gel electrophoresis.

Supplementary Figure 3

```
Atu4351 (15890632)                  ------------MPELFITYENALKRFGLP---ENPEIMGEADIARYKNRIPETYLDFIRHAGLGIWKQGYFQFCNPEKY :  65
Atu3639 (159185838)                 ---------MVEQPNYNVFETKWIEELLNAYEIDHIVKPSAQIVERYKDRLPEILVKFWIEKGWCSWSKGQYWICDPSIF :  71
Rleg4DRAFT_5192 (393183393)         ------------MNNYQARLDSIVQDFGSP---ESG--TAALDLGHYRGKVPEAMIDFWQQNGIGAVLDGYFQFCDPGQY :  63
PJE062_861 (211959674)              ------------MLSEDFESLFEDIDRPRE-----FRKIQSSEAKSLKESVPDDLFEYFTVYGRSILFEGRYQTCHPDDM :  63
BUC_4515 (7393834)                  MPSLSDANSEDMVMDNFMENFLGFEGFGPP---MARRDVPLEKIEKFRGKLPNKLLEYWQEYGWCGYAKGLLWTVDPDEW :  77
VCHE48_1082 (40049333)              ---MSFKFQWTAKMQQGFDEFEHSMGVHRGFPIVSSTPVPAEVLEQYNGVLPENLLAYWERHGWCGYDNGMFWTVNPNDY :  77
VMA_001768 (262025038)              ---MAFKFQWSEVMDSKFERFQRRMGTA-----FVSQEVPLQALEYYEGILPTNLLAYWERYGWCGYNNGLYWTVNPDDY :  72
A1S_0552 (126640623)                --------------------------------------MTEDIIKKYRGKLPESILEQWHLFGFAGYLNGLYWITNPDDY :  42
F971_00412 (479947886)              -------------MDENFEIFYHDEGFGPP---VCVEPVSEETLKKYQGKLPNQLLEYWRAFGFSGWGNGLFWLVNPEDY :  64
ATW7_01785 (119444997)              -----------------------------------STPVTNETLQKFKGRLPSRLLEYWQEYGFCGFMDGFFWIVDPDDY :  45
Swoo_2339 (70726687)                -------------MNKFFDNFYNFAGFGPA---VKSKQPTAEELEFFEDKLPNRLLEYWQEYGFCGWGEGLIWVVNPADY :  64
Pput_0806 (148546052)               -------------MDKVFASLI--ENFGPP---IDRREVPGSSIERYRDKLPPKLLEYWSEHGWGGYGEGIFWLVNPQEY :  62
PSYMO_00280 (330886099)             -------------MDEDYAFFL--KKFGPA---IEQQAVPPSSIERYKKRLPEQLLKYWEDFGWCGYAKGLFWTVNPQDY :  62
PSYR_0687 (66043954)                -------------MDKVFARFL--EKFGGP---VDRQEVPASSIERYRGKLPSQLLEYWTEHGWCGYGDGIFWIVNPQEY :  62
PSPTO_2458 (28869653)               ------------MRDESFEIFV--DAMGEP---VKVCPASESVIDKYKGVLPDVLLDYWRSEGWCGYADGLFWTVNPDEY :  63
D187_004202 (528053359)             ------------MRDEAFEVFI--EDIGEA---SFRTPALELSFEKYQKVLPKQFLEYWKEEGWCGYAEGLFWTVNPEDY :  63
C800_03412 (507739970)              -------------MYEMFLFNSVNSKITQN--------VNEEFILKYSDYSCEQLNSLWKEVGLGSYYNGLFKIIEPNDL :  59
BCERE0025_58750 (228709321)         -------------------MEQYLKDFKLH------NKVPSEIIEKYKRVVPNEIINLWSNYGFGTFMQGYFKSVNPEEF :  55
CLONEX_01717 (210151382)            --------------------MSIFSDFKEN------YKFDNITEEKIKKIFPKELSDILCNYGSGDFLNGYFRFINPFEY :  54
SEVCU071_1533 (365224744)           --------------------MIEIRDFKKV------ANVPQEIINKYENILSEEIIEFWKDYGLGTFFEGFMKSINPEEY :  54
G362_17755 (516967440)              --------------------MVQIEDFVAH------SPVSQEVIAAYRDRVPSEIVEIWEQYGYGTFGEGFIRVINPSVF :  54
OUW_20591 (382939291)               ------------MADEDFEYFLTKLPLSIP-----GPACTDEHVRVYTSLVPDCLISYWQEFGFSGFGNGTAWLVDPIEW :  63
Tpau_0236 (296137982)               ------------MTDDHFELFLRDVPLTTA-----GPACTQQHLDTYRGVLPNPLLSYWQEYGFSGFGDGIVWLTDPLEW :  63
```

GAD-like domain

```
Atu4351 (15890632)                  KSIVALALGGDKQLN----PVRTHALGFSAFGKILAWNEDY-KTTEINILLHRVTCRGLFKEIP-AERSDINLGIA--VE : 137
Atu3639 (159185838)                 QPVIDYVFLGDSELD----PTRMVAFGYNAFGNVDIWYGDATIRLNLPNGMVRVEPRGYDEGQK-RQWTDEVMIGLKLSF : 146
Rleg4DRAFT_5192 (393183393)         SGILKLVFGGDTDIR----PEQTHALGFGAFGTIIAWNEVH-QDVTIDLVKGQVSCSALVN----GKRYDPNLAVTGQLM : 134
PJE062_861 (211959674)              RGVLSLIFRADKDFT----HKNCHAFLFSSFGEISFWHQEHGYGSVNLLSGEVICRSFTKKTVR----GPGFLKTFIVGF : 135
BUC_4515 (7393834)                  EDELDAWVGETEFME----RDAYYVIARTAFGELILWGEKTGQSLKIITPYGMIFPSFDESK-FERRGPDLSIQLFF--S : 150
VCHE48_1082 (40049333)              TEIIERLSAISQLDD----PSENFVIARGAFGDLEIWNRKRGNVMCYCPVIGLLYQWHIDVKQE-KTEAELESAMASFFW : 152
VMA_001768 (262025038)              REIIERLSAISQLDD----PSENFVIARGAFGDLEIWNRKRGNVMYYSPVIGLLYQWDIDVKPE-KTEAELESDMASFFW : 147
A1S_0552 (126640623)                AEVIYDWLEETPLPD----DDVYHVLARSAFGELLIWGERNYGRYYIKTMEGILHDNGEQLES-----AEFYGSDFF--F : 111
F971_00412 (479947886)              QDVLDAWLEHIELPP----HEEYFVIARTAFGDLSIWGTIHGRCFTISAATSQIFPKMEKME---QGEEDLLIKIFF--S : 135
ATW7_01785 (119444997)              EDVLDAWIDDTFIVE----TDSFYVIGRNAFGDLYLWGEKTGYKYKISTSNGWIIEKVGDENDIKKNNALRAIKMFFYKS : 121
Swoo_2339 (70726687)                ADILMTWLSDTPFEN----ADNYYVIARSAFGELIVWGEKTGQSIDINVNFGMIFPTDNTEK-LKKRGEERSIELFF--A : 137
Pput_0806 (148546052)               DAVVSCWIAGTALAS----HDSYHLVARSAFGDLYLWGEKTGFSLEITSVGSQYIFYRTEFTK---EQLNTELQGFI--L : 133
PSYMO_00280 (330886099)             EDLLTDWLVGKEAFK----NDNYHVIARSAFGELYLWGEKSADSLSITSYMSRYSTNNSIFAD---GEKDFGIRVFF--S : 133
PSYR_0687 (66043954)                EGVVASWLEGTKFEK----RDNYHLIARGAFGDLYLWGEKTGFSLKITSVFSRCVIHDFEITT---EEMNRELQGFL--L : 133
PSPTO_2458 (28869653)               KHLLDMWLSGTKFSG----IDNYHVIARSAFGDLYAWGEKYGRKIIVSCPGDYIVSLADEAEIR-CDDPDLAIQSFF--A : 136
D187_004202 (528053359)             EGLVERWLSGTPFET----LDKYHVVARSAFGKLYAWGERNHRTLVILCPTSALIGIEKELRTP-AKDPDIAAQVFF--A : 136
C800_03412 (507739970)              KDIINQCYIMDDDES-------LLPFMCTAFGDVFAYVKNKRFGNYVVFLNIRYGTSLIIPDN------------FVAIF : 120
BCERE0025_58750 (228709321)         KEILEESSQR---------YKDSIVLFATGMGDLVIWS--DGYVRLLNYRYGIVKTIMFTFE-------------FFFQN : 111
CLONEX_01717 (210151382)            QEVIKDTYFD---------AENSLPFMITAFGDVIVYKK-DGYIGIIKYKEKESAIIGKKIS-------------LFIRF : 111
SEVCU071_1533 (365224744)           TDLMEYGTQF---------FKDAVPLFVTGLGDIIYVEKKDGFMGILKFRYKETKVLMQMFN-------------LTIKL : 112
G362_17755 (516967440)              EAELGDRIGKTQGDG------IAIPVMVTALGDLITWEPSVDSLVAILFRKFETVGLGSPKT-------------FLG-- : 113
OUW_20591 (382939291)               KVTTEEVLLDVINHPRLGEDAKYIPFARSAFGKVWFWTPGYGISVIVDPVRGTMFFRQPARDSSVSGLEGTMKAFFG--- : 140
Tpau_0236 (296137982)               QATTTLITAGITHRP-LGTDATYIPILRTAFGKIWFWTPGFGRSLTITPAVGTASCIIQSAPRDLS-----LQATFA--- : 134
```

GAD-like domain

```
Atu4351 (15890632)                  GIDAESFDAPDEKGKL-MFNRLLKNLGKLQLGQIYSPKLHPSLGG-QLTVENMRPVDALSAMTIAAQAGPFTLYDTTKPS : 215
Atu3639 (159185838)                 NVSPYVAPWEDEKYQN-MMPLALERIGQLEPGEIYGFAPALSIGG-RNNVEHLQKVKVPEHLLLLASLDSPTLYDYSPPS : 224
Rleg4DRAFT_5192 (393183393)         LIDDPTLDEYDANAKK-LFKRARSKLGKLGVGQIYGFRPILALGG-NRALANLTVYEALPHMAILAQAHEMQLMDNAPFP : 212
PJE062_861 (211959674)              ASETFEYDFYDTNDRP-LFSRARRKLGEIGIGECYGFVPALGLGG-VPELETLKKLKAPEHFAILAQLIDFQLIDVQGYG : 213
BUC_4515 (7393834)                  TCSKEAFDFLDNDKAA-LFERALEKLGPLDHTTMYGFVPALALGG-TPMLERLQKLDAHVHLDILSQVTELHVMRDIAQD : 228
VCHE48_1082 (40049333)              RASSSDQDQEDEYEEP-LFERALDKLGPLAENEMYAFVPATCLGG-KWLLENLQKVDITTHIEMLMDLEEPRINIMR--- : 227
VMA_001768 (262025038)              TASSSDQDQEDEYEEP-LFERALDKLGPLAENEMYAFVPATCLGG-KWLLENLQKVDITTHIEMLMDLEEPRINIMR--- : 222
A1S_0552 (126640623)                LPKKNYLDYTDKNGNK-LFDRAVKKLGVLKADEMYAFEPALALGG-EESLQHLTKVNLPVHMKLLKQVTPLRLRTFEDLT : 189
F971_00412 (479947886)              SKDKESLDINDYKNKP-LFDRAVKKYGELSKNEMFGFEPALILGG-EAKLENVRKLPIISHLQFLASLDTPRMMLDIGKF : 213
ATW7_01785 (119444997)              AMKVEAFDFIDIDQEP-LFGRCKELHNPLEYDEVFAFEPAIFLGG-EPRIENITKVNIFAHLTMLASFGEKEILNQESLI : 199
Swoo_2339 (70726687)                SMSKDSLDKKDLDENP-LFERAMAKLGPLEADEMYGFVPALAIGG-APKLENLQKVKVLEHLTFLADLGEKTVMADIVAM : 215
Pput_0806 (148546052)               SREVESMDFNG------LFNPAKEKLGRLKHDEMYGFFPALMLGG-ADSLQHLKKVSAVEHLIFLAQLTDLQPYSFSEQP : 206
PSYMO_00280 (330886099)             SMQLEHNDLDS------FFEPALKNLGQLQSDEMYGFVPALALGG-PVELKNLQKVKTTEHLTFLSQLSPLQDWGFPDV- : 205
PSYR_0687 (66043954)                SRNVDSNDLED------LFKPARRQLGTLRHDEMYGFVPALMLGG-SASLDHIEKLKAVEHLILLSQLAELQPYRF---- : 202
PSPTO_2458 (28869653)               MSDREDFDMEDHDGIP-LFERAVQKLGCLNEDEMYGFQPALVLGG-SITLQNLVKLDLEVHLTILHQLSR---------- : 204
D187_004202 (528053359)             SKKKQELDMKDEAGKA-LFQRALKKLGPLAPDEMYGFEPALVAGG-RNRLENLQKVNLFVHLAIVRELAEPR-IPFAGVD : 213
C800_03412 (507739970)              NKVIPNQSFLKGWFDLENYAFVKEKIGEIDFDECYGYFPTLSMGG-NESIDNISIVKMIPYIDMNVQMIDVFERADKL-- : 197
BCERE0025_58750 (228709321)         IT---DLEFRDEDLSWQPYPEALKQYNELDYEECFGYTPLLGLGG-AKKVENLKKVKLKEHILIITSFMGPVQ------- : 180
CLONEX_01717 (210151382)            LE---DDSFKKINFDIPLYEDAIKLYGKLQNNECFGFVPLLPLGG-KKEANCLEKVNIKVHIELITELVGKIE------- : 180
SEVCU071_1533 (365224744)           IIDENDKYFCDSHIEPQMYFEALKTKEEPAYDECFGYVPILPLGG-KEEVAHMDSVKIREHILLITELTGPIND------ : 185
G362_17755 (516967440)              LTRLDGAEHLEEEFAWDPYPEAVARHGAVEFDESFIFVPLPSLGG-AGTVDTLQKRPTLSAIQVMVDLQGPIGH------ : 186
OUW_20591 (382939291)               ASDGERFDFLDNSEQP-MFDRVYEHLGALRFDELYGFAPGLRIGG-AAIVEATHLFQIHVHMALLRSVIGD--AWYVAG- : 215
Tpau_0236 (296137982)               TGDRERYDFDPKGFPGGLFEEQVLTRCGPLTVDQVYHFTPSIVSDDDPVDISTAQVANIHEWLAAVKTEVGDWSTFYI--- : 211
```

DUF1851

Figure S3. Sequence alignment of the Tdi immunity protein family, related to Figure 7A. BLASTP analysis was performed and full-length sequence alignment with ClustalW showed 2 conserved domains of representative Tdi family proteins. The locus tag and GI number are on the left and the amino acid position of residues is on the right of the sequences. The GAD-like domain and the DUF1851 domain are underlined in blue and yellow, respectively. The conserved amino acid residues are shaded in black for identity and in grey for similarity.

Supplementary Figure 4

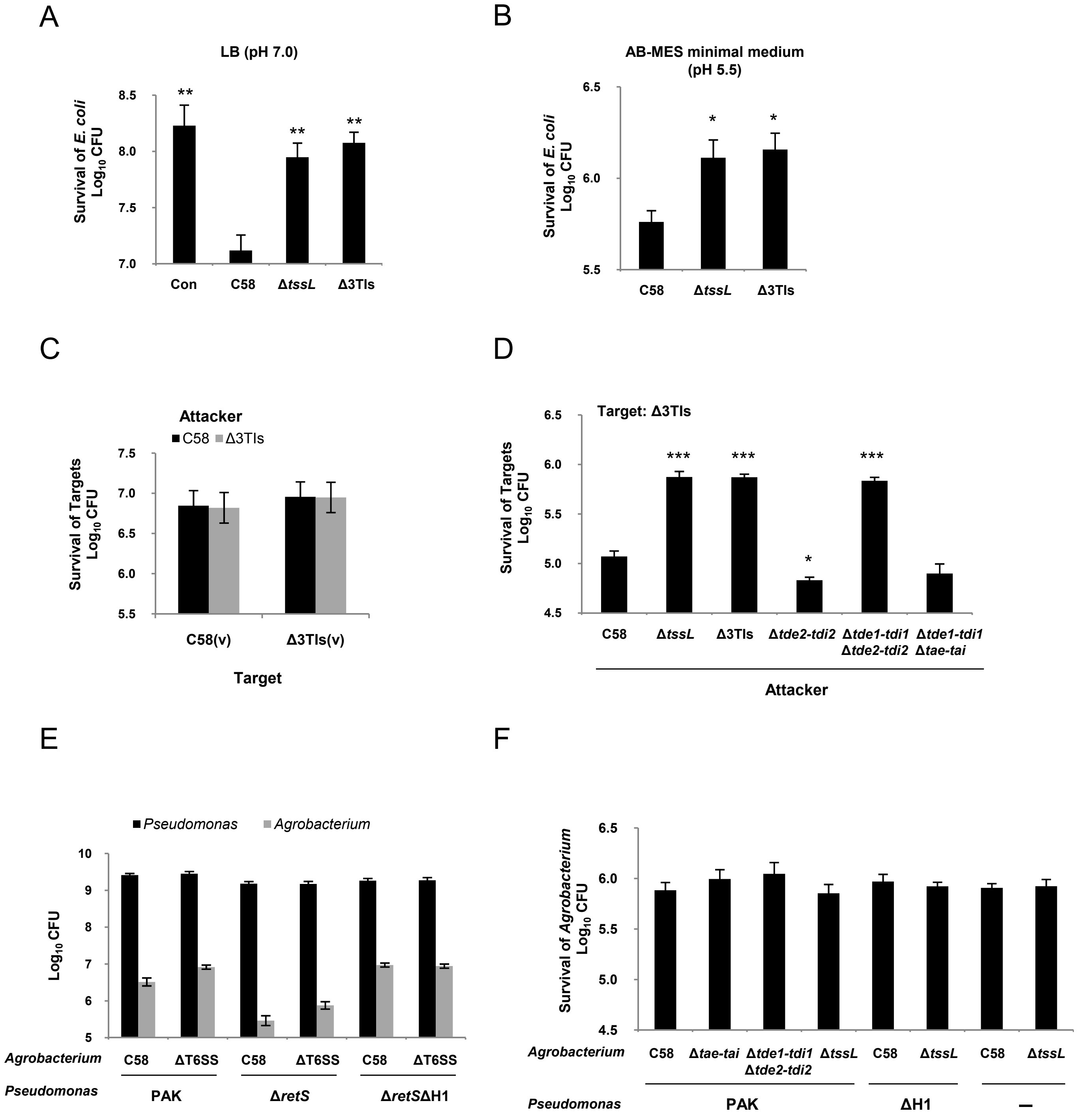

Figure S4. **Interbacterial competition assays, related to Figure 4 and 5.** (A) *A. tumefaciens* antibacterial activity assay against *E. coli* on LB. The *A. tumefaciens* wild-type C58, Δ*tssL*, or Δ3TIs mutant was co-cultured on LB (pH 7.0) agar with *E. coli* strain DH10B cells harboring the plasmid pRL662 to confer gentamicin resistance, at a ratio of 10:1. *E. coli* alone without contact with *A. tumefaciens* serves as a control (Con). (B) *A. tumefaciens* antibacterial activity assay against *E. coli* on AB-MES agar. The *A. tumefaciens* wild-type C58, Δ*tssL*, or Δ3TIs mutant was co-cultured on AB-MES (pH 5.5) agar with *E. coli* strain DH10B cells harboring the plasmid pRL662 at a ratio of 10:1. (C) *A. tumefaciens* intra-species competition on agar. The *A. tumefaciens* attacker strain (C58 or Δ3TIs) was mixed with the target strain (C58 or Δ3TIs) harboring pRL662 that confers gentamicin resistance at a 100:1 (attacker: target) ratio and co-cultured on AB-MES (pH 5.5) agar. The survival of target cells was quantified and no significant difference could be detected. Similar results were obtained by 10:1 (attacker: target) ratio (data not shown). (D) *A. tumefaciens* intra-species competition *in planta*. The *A. tumefaciens* attacker strain was mixed with the target strain harboring a gentamicin resistance-encoding vector pRL662 at a 10:1 (attacker: target) ratio, infiltrated into *N. benthamiana* leaves, and incubated at room temperature for 24 hr. The survival of target cells was quantified. (E) Cells of *P. aeruginosa* was mixed equally with *A. tumefaciens* harboring pRL662 and co-cultured at 28°C for 16 hr on LB agar. The survival of *P. aeruginosa* cells was quantified by growth on LB agar at 37°C for 12–16 hr before the emergence of visible *A. tumefaciens* colonies, which were quantified by growth on gentamicin-containing LB agar at 28°C for 48 hr. (F) Cells of *P. aeruginosa* and *A. tumefaciens* harboring pRL662 were mixed equally and infiltrated into *N. benthamiana* leaves and incubated at room temperature for 24 hr. The survival of *A. tumefaciens* cells was quantified by growth on gentamicin-containing LB agar. Data are mean ± SE of four biological replicates from three independent experiments (A, B) or three to six biological replicates from a minimum of two independent experiments (C, D, E, F). Significant difference compared with C58 was denoted as ***=P <0.0005, **=P <0.005, and *=P <0.05.

Supplementary Figure 5

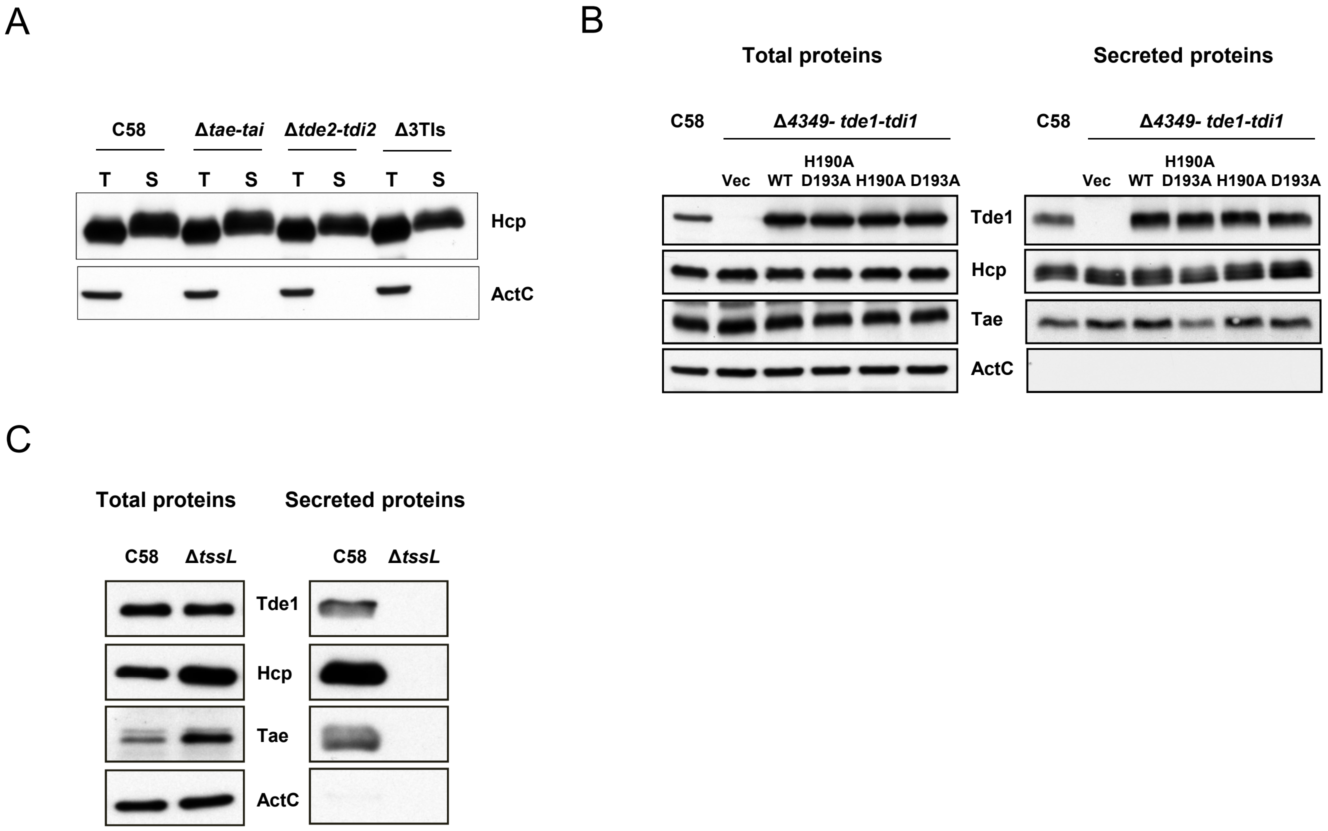

Figure S5. **Secretion assay, related to Figure 4 and Figure 5.** (A) Hcp secretion assay with wild-type *A. tumefaciens* C58, Δ*tae1–tai1*, Δ*tde2–tdi2*, and Δ3TIs (Δ*tae-tai* Δ*tde1–tdi1* Δ*tde2–tdi2*) grown in AB-MES (pH 5.5) liquid culture. Total (T) and secreted (S) proteins were isolated for western blot analysis of Hcp and ActC. ActC was a non-secreted protein control. (B) Secretion assay for various Atu4350 (Tde1) variants. Total and secreted proteins were isolated from the *A. tumefaciens* Δ*4349-tde1-tdi1* mutant containing vector pTrc200 (Vec) or derivatives expressing wild-type (WT) or HxxD variants of Tde1 grown on AB-MES minimal agar (pH 5.5) for western blot analysis of Tde1, Hcp, and Tae. (C) Secretion assay in LB medium. Total and secreted proteins were isolated from wild-type C58 and Δ*tssL* mutant grown in LB broth (pH 7.0) for 4-6 hr at 25°C for western blot analysis of Tde1, Hcp, and Tae.

Supplementary Figure 6

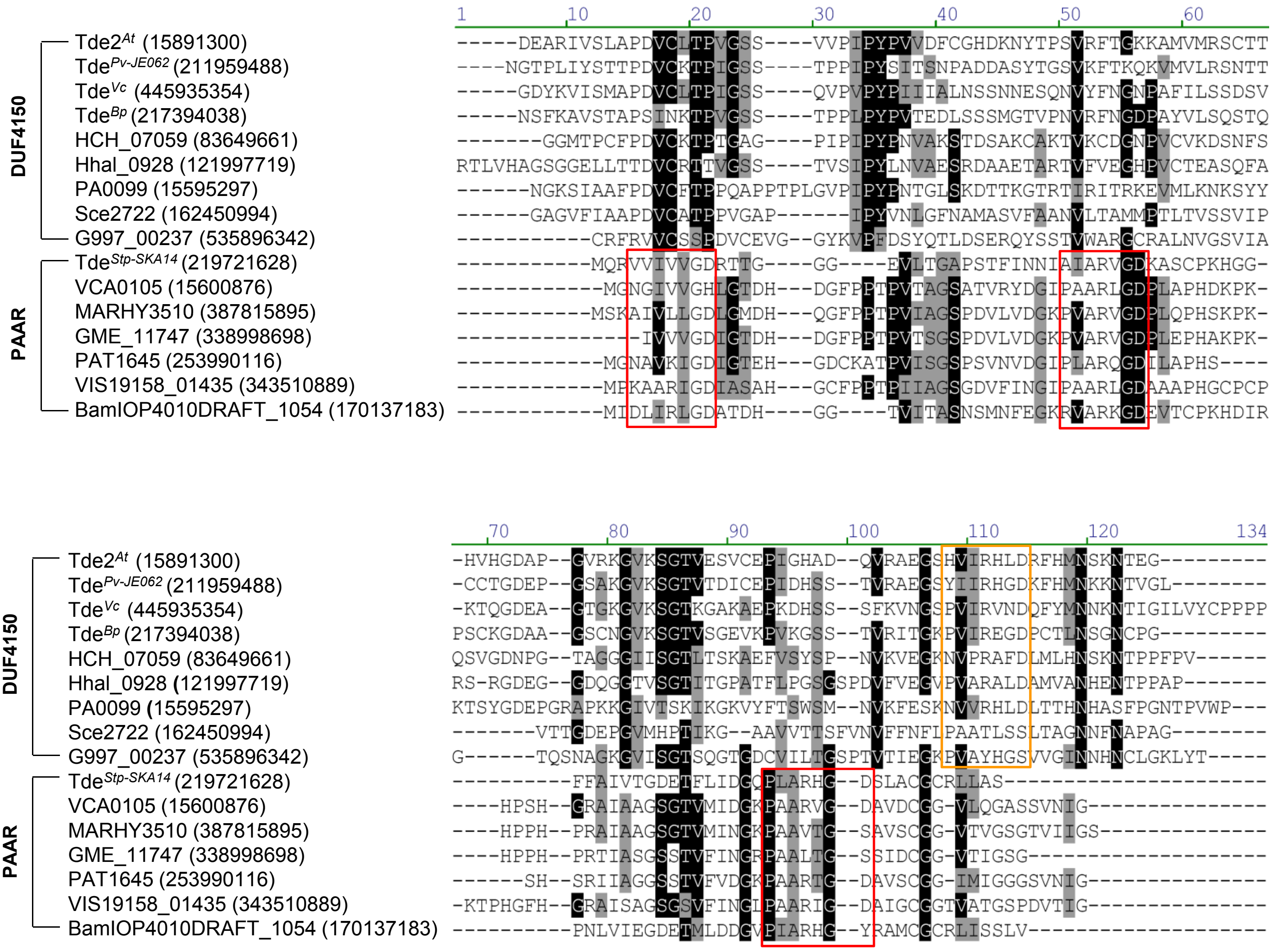

Figure S6. **Sequence alignment of DUF4150 and PAAR domains, related to Figure 7B.** Sequence alignment of DUF4150 domain and PAAR domain from selected Pfam family proteins was generated by using ClustalW. The locus tag and GI number are on the left and the amino acid position of residues is on the right of the sequences. The conserved amino acid residues are shaded in black for identity and in grey for similarity. PAARxGD motif is shown in a red box for PAAR-domain proteins and an orange box for DUF4150 domain proteins.

Supplementary Figure 7

```
                                         *         20         *         40         *         60
Tde^At (15890633 )            ---------KQVEEFRRQLKEQQDEINRMEPDDLVRNIDKYRQQGRPTDDAANRRQSRED
Tde^Bp (217394038)            --------KGDPKEYDRQLLEQEKGLNDLSVKEYLEGRARYQEIGRAGTGAA-QEQARAK
Tde^Vc (445935354)            --------KIDDYINNQSLLEDKKAQHGLTSEKAVQK--KLKEFG---------MEAEAK
Tde^Pp (48546051)             --------EYKIGEFKRQLNGQEDGLNWMTVEEFLE---KVENPNQRNKGLA-QR-ARKK
RhsA (307129607)              ----------------AELRTGSGTNQ--SSRDYARS--------------------LGN
RhsB (307131672)              -----------------KIGDRQKGMIKDKLSTVKER--------------------SKA
Cdi-CT_o11^Ec (446167868)     ----TATATVNASEVQGMQWGQGNMKQGMPWEDYVGKSLPADARLPKNFKIFDYYDGATK
Colicin E7 (510385)           KRNKPGKATGKGKPVNNKWLNNAGKDLGSPVPDRIAN---------------KLRDKEFK
Pyocin S3 (854363)            -------VKPLEVDIYGNFAGRPRDGN--HLDHMPDQ---------------------GA
CdiA -CT^Dda (307131201)      -------VKPLDVGSYKELKDRAVVGDGLEHDHIPSF---------------------AA

                                         *         80         *        100         *        120
Tde^At (15890633 )            YRTDRTRELEEKYLS---KGRNDYKEQAANDVAEEMKKLA------ATHTLDLVAGGDGS
Tde^Bp (217394038)            YSRELANQFKDALNE-QGIFGKAAKEQAEAMAADRMKTLA------ALHNPDMIAGGKDV
Tde^Vc (445935354)            KRGWSKETIIRNV-T------------AMADSQHEMRSLA------ALHNPDMVAGGVDK
Tde^Pp (48546051)             EQEFIKEKIEKELRKTMGPF--EAQRVAIEKARNRMSITA------ALHNPDLVAGGRDV
RhsA (307129607)              QTDDAGHILGNVLGG-------------QGGKGNVFPQLP------AINRGQYRDFEKVV
RhsB (307131672)              LNTKMREHFNANEQK----------------IISEWEKQT------GMNWPTLSSGSRAT
Cdi-CT_o11^Ec (446167868)     TATSVKSIDTQTMAK-------------LANPNQVYSSIKGNIDAAAKFKEYALSGRELT
Colicin E7 (510385)           SFDDFRKKFWEEVSK-------------DPELSKQFSRNN-------NDRMKVGKAPKTR
Pyocin S3 (854363)            LATSLR-AIYPDIPY--------------GEIRKLMKKGG------SVAIPARVHQR--F
CdiA -CT^Dda (307131201)      LRTAKENELGRKLTP--------------AEEKTLYQNAT------AVEVPKDVHR---A

                                         *        140         *        160         *
Tde^At (15890633 )            ISGLGDKSINSSLGSQWKG----RRSEQLRSHAKKAAEQK----KKMNAKLE--
Tde^Bp (217394038)            VTMMGDRGVNSSIGSQWKD-----RVAELDDAAKKVPESE-RGGTKMNAKLK--
Tde^Vc (445935354)            ITGFGDKNTNSMIGASWSSGKENSRVSLLDKQACKEANEKGNGSKKMNVEL---
Tde^Pp (48546051)             ISDFGDRQVNSVIGAQWKT-----RIPNLKKAAERVSLES-RGSIRMNVKLH--
RhsA (307129607)              KDYIGQHGS---VDIEWAF--------KYGNGGTRPTEI---------------
RhsB (307131672)              PHHVIPIKNGG--SNEWWN---------IIPVQHPHTGTI--------------
Cdi-CT_o11^Ec (446167868)     SSMISNREIQLAIPADTTK--------TQWAEINRAIEYGKSQGVKVTVTQVK-
Colicin E7 (510385)           TQDVSGKRTSFELHHEKPIS---------QNGGVYDMDNISVVTPKRHIDIHRG
Pyocin S3 (854363)            SETYGGRNTKEKQQKDASD---------LRAAVDSNFDAVKKGLLEEGF-----
CdiA -CT^Dda (307131201)      GPTYGGKNTAAQVQQDALD---------LCGAVCRDTDALRTNMIERGY-----
```

Figure S7. The Tde family is distinct from known DNase toxins, related to Figure 2A. The unique toxin domains from *A. tumefaciens* Tde1^{At}, *B. pseudomallei* Tde^{Bp}, *V. cholerae* Tde^{Vc}, *P. putida* Tde^{Pp}, *D. dadantii* 3937 RhsA and RhsB, *E. coli* colicin E7, *P. aeruginosa* Pyocin S3, *D. dadantii* 3937 CdiA-CT^{Dda}, and *E. coli* 869 CdiA-CT$_{o11}^{Ec}$ were aligned by use of ClustalW. The conserved amino acid residues identified among Tde family proteins are shaded in black. The locus tag and GI number are on the left of each sequence.

SUPPLEMENTAL EXPERIMENTAL PROCEDURES

Protein purification

C-terminal His-tagged Tde1 (Tde1-His) and Atu4349 proteins were co-expressed in *E. coli* DH10B cells with the plasmids pJN105 and pTrc200, respectively. *E. coli* cells were grown to OD_{600} 0.7 in the presence of 0.5% glucose and 1 mM IPTG. Cells were harvested and resuspended in fresh LB medium with 0.2% L-arabinose and 1 mM IPTG. Growth was continued for another 2 hr to induce production of Tde1-His. The proteins were purified to homogeneity by nickel chromatography as previously described (Ma et al., 2012). Briefly, cells were lysed in Buffer A (20 mM Tris-Cl, 0.3 M NaCl, 0.5 mM DTT, 20 mM imidazole, and 20% glycerol, pH 7.5) and proteins were finally eluted from the nickel column in Buffer B (20 mM Tris-Cl, 50 mM NaCl, 250 mM imidazole, and 20% glycerol, pH 7.5).

In vitro RNase activity assay

Total RNA was extracted from *E. coli* DH10B (Lin et al., 2013) and 2 µg of total RNA was incubated with 0.2 µg of bovine serum albumin (BSA), Tde1, or RNase A in 10 µl of 50 mM Tris/HCl, 100 mM NaCl, and 10 mM $MgCl_2$ for 15 min at 37°C. The integrity of RNA was analyzed by RNA-formaldehyde gel.

TUNEL (terminal deoxynucleotidyl transferase dUTP nick-end labelling) and FACS (fluorescence-activated cell sorting) analysis

Overnight culture of *E. coli* DH10B strains harboring the pJN105 vector or derivatives expressing Tde toxins were harvested and adjusted to OD_{600} 0.3 with LB agar containing 0.2% L-arabinose. After 2-hr induction, cells (OD_{600} ~0.5) were washed with PBS buffer, fixed, and stained by use of the Apo-Direct Kit (BD Bioscience). The 3'-OH end of fragmented DNA was labelled with FITC-dUTP by terminal deoxynucleotidyl transferase. Propidium iodide (PI) labelling both intact and fragmented DNA was used as counterstaining. The PI-labelled and FITC-unlabelled *E. coli* cells were first gated at 10^2 with an FSC trigger with a threshold of 0.1%. The

FITC fluorescence was detected with a 529/28-nm filter in FL1 channel. The FL1-H signal < 10^2 was set as negative and > 10^2 was FITC-positive. At least 100,000 cells were collected for each sample. The intensity of fluorescence was determined by FACS analysis with the MoFlo XDP Cell Sorter (Beckman Coulter) and Summit V 5.2 software.

Interbacterial competition on agar plates

For interbacterial competition assay between *P. aeruginosa* and *A. tumefaciens,* overnight cultures of *P. aeruginosa* and *A. tumefaciens* containing pRL662 derivative conferring gentamicin resistance were grown in LB or 523 medium at 37°C and 28°C, respectively. The bacteria were sub-cultured for further growth for 4 to 5 hr under the same conditions. The bacterial cells were adjusted to OD_{600} 0.01, mixed at a 1:1 ratio, and 10 µl was spotted on LB (pH7.0) agar and incubated for 16 hr at 28°C. Cells were harvested, serially diluted, and plated in triplicates on LB agar with or without gentamicin for colony forming units (CFU) counting. Because of the higher replication rate of *P. aeruginosa*, the *P. aeruginosa* cell number was scored after 16-hr incubation at 37°C on LB agar without any antibiotics. *A. tumefaciens* cells were counted on gentamicin-containing LB agar plates after 2- days' incubation at 28°C. Similar procedures were used for *E. coli-A. tumefaciens* and *A. tumefaciens* intra-species competition assay except that the bacterial cells were co-cultured at a ratio of 10 (*A. tumefaciens* attacker cells at OD_{600} 0.1) to 1 (*A. tumefaciens* or *E. coli* DH10B harboring pRL662 target cells at OD_{600} 0.01) and grown on LB (pH7.0) or AB-MES (pH5.5) agar plates at 25°C for 16 hr. Target *E. coli* and *A. tumefaciens* cells were counted on gentamicin-containing LB agar plates for 16 hr at 37°C and 2-days' incubation at 28°C, respectively. At least three independent experiments or minimum of three biological replicates from two independent experiments were performed for all assays. Data represent mean ± standard error (SE) of all biological replicates. Statistics was calculated by Student's t test and the p-value was denoted as ***=P <0.0005, **=P <0.005, and *=P <0.05.

Interbacterial competition assay *in planta*

The intra-species *A. tumefaciens* competition assay was performed with a 10:1 attacker-to-target ratio by leaf infiltration of *Nicotiana benthamiana*. Briefly, 523 overnight-cultured *A. tumefaciens* cells were sub-cultured at 28°C in the same medium for further growth to OD_{600} 1.0-1.5. The harvested cells were resuspended in 1/2 Murashige and Skoog (MS) medium (pH 5.7) to an appropriate OD_{600}. The attacker (OD_{600} 5) and target (OD_{600} 0.5) were mixed equally before infiltration into 2-month-old leaves of *N. benthamiana* with use of a needleless syringe. After 24-hr

incubation at room temperature, the infiltrated spot was punched out, ground in 0.9% NaCl, serially diluted, and plated in triplicates on LB agar containing appropriate antibiotic to select for the target cells. Similar procedures were used for *A. tumefaciens-P. aeruginosa* inter-species competition assay, except the bacterial cells were adjusted to OD_{600} 1 mixed equally for infiltration. All assays were performed with at least two independent experiments and each with two biological replicates; or three independent experiments and each with one or two biological replicates. Data represent mean ± standard error (SE) of all biological replicates. Statistics was calculated by Student's t test and the p-value was denoted as ***=P <0.0005, **=P <0.005, and *=P <0.05.

REFERENCES

Goodman, A.L., Kulasekara, B., Rietsch, A., Boyd, D., Smith, R.S., and Lory, S. (2004). A signaling network reciprocally regulates genes associated with acute infection and chronic persistence in Pseudomonas aeruginosa. Dev. Cell *7*, 745-754.

Hachani, A., Lossi, N.S., and Filloux, A. (2013). A visual assay to monitor T6SS-mediated bacterial competition. J. Vis. Exp. e50103.

Kado, C.I., and Heskett, M.G. (1970). Selective media for isolation of Agrobacterium, Carynebacterium, Erwinia, Pseudomonas, and Xanthomonas. Phytopathology *60*, 969-976.

Lai, E.M., and Kado, C.I. (1998). Processed VirB2 is the major subunit of the promiscuous pilus of Agrobacterium tumefaciens. J. Bacteriol. *180*, 2711-2717.

Lin, J.S., Ma, L.S., and Lai, E.M. (2013). Systematic dissection of the Agrobacterium type VI secretion system reveals machinery and secreted components for subcomplex formation. PLoS One *8*, e67647.

Ma, L.S., Lin, J.S., and Lai, E.M. (2009). An IcmF family protein, $ImpL_M$, is an integral inner membrane protein interacting with $ImpK_L$, and its walker a motif is required for type VI secretion system-mediated Hcp secretion in Agrobacterium tumefaciens. J. Bacteriol. *191*, 4316-4329.

Ma, L.S., Narberhaus, F., and Lai, E.M. (2012). IcmF family protein TssM exhibits ATPase activity and energizes type VI secretion. J. Biol. Chem. *287*, 15610-15621.

Newman, J.R., and Fuqua, C. (1999). Broad-host-range expression vectors that carry the L-arabinose-inducible Escherichia coli araBAD promoter and the araC regulator.

Gene *227*, 197-203.

Quandt, J., and Hynes, M.F. (1993). Versatile suicide vectors which allow direct selection for gene replacement in gram-negative bacteria. Gene *127*, 15-21.

Schmidt-Eisenlohr, H., Domke, N., and Baron, C. (1999). TraC of IncN plasmid pKM101 associates with membranes and extracellular high-molecular-weight structures in Escherichia coli. J. Bacteriol. *181*, 5563-5571.

Studier, F.W., Rosenberg, A.H., Dunn, J.J., and Dubendorff, J.W. (1990). Use of T7 RNA polymerase to direct expression of cloned genes. Methods Enzymol. *185*, 60-89.

Vergunst, A.C., Schrammeijer, B., den Dulk-Ras, A., de Vlaam, C.M., Regensburg-Tuink, T.J., and Hooykaas, P.J. (2000). VirB/D4-dependent protein translocation from Agrobacterium into plant cells. Science *290*, 979-982.
